# Supplementary material for: Drug-Induced Myoclonus: A Systematic Review
Source: Medicina (Kaunas). 2025 Jan 15;61(1):131. doi: 10.3390/medicina61010131 (PMC11767161; doi:10.3390/medicina61010131)
Supplement: Supplementary file 1 [file medicina-61-00131-s001.zip › medicina-3403095-supplementary.pdf]

Supplementary Material 1

We searched the Medline/PubMed database to locate existing reports on myoclonus secondary to medications published until June 2024 in electronic form. The search term was “myoclonus”. The query was (“myoclonus"[MeSH Terms] OR "myoclonus"[All Fields]), which yielded 12097 results. All the results titles and those providing abstracts were assessed. Only studies describing myoclonus associated with a drug were included in the following study. For a complete list of all the publications of DIM in PubMed, consider reading **Table**.

Table S1. Articles Of Myoclonus Associated With Drugs In PubMed.

Number	PMID	Main cause of myoclonus	Year	Reference
1	37558	DDT	1979	Hwang EC, Van Woert MH. DDT-induced myoclonus: serotonin and alpha noradrenergic interaction. Res Commun Chem Pathol Pharmacol. 1979 Feb;23(2):257-66. PMID: 37558.
2	40657	Picrotoxin	1979	Jenner P, Marsden CD, Pratt J, Reynolds EH. Modulation of picrotoxin-induced forepaw myoclonus in the rat by benzodiazepines [proceedings]. Br J Pharmacol. 1979 Nov;67(3):440P. PMID: 40657; PMCID: PMC2044033.
3	41197	5-hydroxytryptophan	1979	Weiner WJ, Carvey PM, Nausieda PA, Klawans HL. Dopaminergic antagonism of L-5-hydroxytryptophan-induced myoclonic jumping behavior. Neurology. 1979 Dec;29(12):1622-5. doi: 10.1212/wnl.29.12.1622. PMID: 41197.
4	48126	Etomidate	1975	Morgan M, Lumley J, Whitwam JG. Etomidate, a new water-soluble non-barbiturate intravenous induction agent. Lancet. 1975 Apr 26;1(7913):955-6. doi: 10.1016/s0140-6736(75)92011-5. PMID: 48126.
5	55381	Chlorinated hydrocarbon	1975	Gupta PC. Neurotoxicity of chronic chlorinated hydrocarbon insecticide poisoning--a clinical and electroencephalographic study in man. Indian J Med Res. 1975 Apr;63(4):601-6. PMID: 55381.
6	77316	Levodopa, bromocriptine	1978	Vardi J, Glaubman H, Rabey JM, Streifler M. Myoclonic attacks induced by L-dopa and bromocryptin in Parkinson patients: a sleep EEG study. J Neurol. 1978 Apr 14;218(1):35-42. doi: 10.1007/BF00314716. PMID: 77316.
7	85189	Ticarcillin	1979	Kallay MC, Tabechian H, Riley GR, Chessin LN. Neurotoxicity due to ticarcillin in patient with renal failure. Lancet. 1979 Mar 17;1(8116):608-9. doi: 10.1016/s0140-6736(79)91032-8. PMID: 85189.
8	93500	Catechol	1979	Chadwick D, Jenner P, Marsden CD. 5-Hydroxytryptamine and myoclonus induced by 1,2-di-hydroxybenzene (catechol) in the guinea-pig. Br J Pharmacol. 1979 Jul;66(3):358-60. doi: 10.1111/j.1476-5381.1979.tb10837.x. PMID: 93500; PMCID: PMC2043656.
9	109997	Bismuth	1979	Eichler I. Die Wismutenzephalopathie - ein neuartiges Krankheitsbild? [The bismuth-encephalopathy - a new pathological syndrome (author's transl)]. Wien Klin Wochenschr. 1979 Apr 27;91(9):314-20. German. PMID: 109997.
10	116267	Cocaine	1979	Greer CA, Alpern HP. Maturational changes related to dopamine in the effects of d-amphetamine, cocaine, nicotine, and strychnine on seizure susceptibility. Psychopharmacology (Berl). 1979 Sep;64(3):255-60. doi: 10.1007/BF00427507. PMID: 116267.
11	138798	5-hydroxytryptophan	1977	Coleman M. Myoclonus in the young after 5-hydroxytryptophan. N Engl J Med. 1977 Apr 7;296(14):820. doi: 10.1056/NEJM197704072961419. PMID: 138798.
12	149954	Diclofenac	1978	Bandelot JB, Mihout B. Encéphalopathie myoclonique imputable au diclofénac [Myoclonic encephalopathy due to diclofenac]. Nouv Presse Med. 1978 Apr 22;7(16):1406. French. PMID: 149954.
13	198893	Bismuth	1977	Hillemand P, Pallière M, Laquais B, Bouvet P. Traitement bismuthique et bismuthémie [Bismuth treatment and blood bismuth levels]. Sem Hop. 1977 Sep;53(31-32):1663-9. French. PMID: 198893.
14	223724	Morphine	1979	Meldrum BS, Menini C, Stutzmann JM, Naquet R. Effects of opiate-like peptides, morphine, and naloxone in the photosensitive baboon, Papio papio. Brain Res. 1979 Jul 13;170(2):333-48. doi: 10.1016/0006-8993(79)90111-2. PMID: 223724.
15	224483	Diclofenac	1979	Alcalay M, Thomas P, Reboux JF, Bontoux D. Myoclonies au cours d'un traitement par le diclofenac [Myoclonus during a treatment with diclofenac (author's transl)]. Sem Hop. 1979 Apr 8-15;55(13-14):679-80. French. PMID: 224483.
16	302717	5-hydroxytryptophan	1977	Chadwick D, Hallet M, Jenner P, Marsden CD. 5-Hydroxytryptophan-induced behaviour in guinea-pigs: its relevance to human myoclonus [proceedings]. Br J Clin Pharmacol. 1977 Jun;4(3):402P-403P. PMID: 302717.
17	303572	5-hydroxytryptophan	1977	Weiner WJ, Goetz C, Nausieda PA, Klawans HL. Clonazepam and 5-hydroxytryptophan-induced myoclonic stereotypy. Eur J Pharmacol. 1977 Nov 1;46(1):21-4. doi: 10.1016/0014-2999(77)90139-x. PMID: 303572.
18	304885	5-hydroxytryptophan	1978	Chadwick D, Hallett M, Jenner P, Marsden CD. 5-hydroxytryptophan-induced myoclonus in guinea pigs. A physiological and pharmacological investigations. J Neurol Sci. 1978 Jan;35(1):157-65. doi: 10.1016/0022-510x(78)90108-9. PMID: 304885.

19	310084	5-hydroxytryptophan	1978	Volkman PH, Lorens SA, Kindel GH, Ginos JZ. L-5-Hydroxytryptophan-induced myoclonus in guinea pigs: a model for the study of central serotonin-dopamine interactions. Neuropharmacology. 1978 Nov;17(11):947-55. doi: 10.1016/0028-3908(78)90137-5. PMID: 310084.
20	311446	DDT	1978	Hwang EC, Van Woert MH. p,p'-DDT-induced neurotoxic syndrome: experimental myoclonus. Neurology. 1978 Oct;28(10):1020-5. doi: 10.1212/wnl.28.10.1020. PMID: 311446.
21	318906	Etomidate	1977	Famewo CE, Odugbesan CU. Clinical trial of etomidate. Preliminary observations on a new non-barbiturate induction agent. Can Anaesth Soc J. 1977 Jan;24(1):35-8. doi: 10.1007/BF03006810. PMID: 318906.
22	378555	Etomidate	1979	Oudenaarden JJ. Etomidate as an induction agent in minor operative procedures. Curr Med Res Opin. 1979;6(1):30-4. doi: 10.1185/03007997909109395. PMID: 378555.
23	431301	Isoniazid, hydantoin	1979	Destee A, Verrier A, Gelez P, Warot P. Encéphalopathie myoclonique par l'association isoniazide-hydantoïnes [Myoclonic encephalopathy caused by isoniazid-hydantoin combination]. Lille Med. 1979 Jan;24(1):41-5. French. PMID: 431301.
24	434718	Bismuth	1979	Emile J, De Bray JM, Bernat M, Morer T, Allain P. Les ostéopathies scapulaires et vertébrales des encéphalopathies aiguës myocloniques bismuthiques. A propos de 8 cas [Scapular and vertebral osteopathies during acute myoclonic bismuth encephalopathies. Apropos of 8 cases]. Ann Med Interne (Paris). 1979;130(2):75-80. French. PMID: 434718.
25	478382	Bromocriptine	1979	Glaubman H, Rabey I, Vardi J. [Myoclonic attacks induced by bromocriptine in Parkinsonian patients]. Harefuah. 1979 Jan 15;96(2):90-2. Hebrew. PMID: 478382.
26	508151	Penicillin	1979	Sackellares JC, Smith DB. Myoclonus with electrocerebral silence in a patient receiving penicillin. Arch Neurol. 1979 Dec;36(13):857-8. doi: 10.1001/archneur.1979.00500490071013. PMID: 508151.
27	563003	Chloroamphetamine	1977	Growdon JH. Postural changes, tremor, and myoclonus in the rat immediately following injections of p-chloromaphetamine. Neurology. 1977 Nov;27(11):1074-7. doi: 10.1212/wnl.27.11.1074. PMID: 563003.
28	571982	Spinal anesthesia	1978	Fox EJ, Villanueva R, Schutta HS. Myoclonus following spinal anesthesia. Neurology. 1979 Mar;29(3):379-80. doi: 10.1212/wnl.29.3.379. PMID: 571982.
29	579354	Chlorimipramine	1977	Tseng LF, Harris RA, Loh NH. Blockade of para-methoxyamphetamine- induced serotonergic effects by chlorimipramine. J Pharmacol Exp Ther. 1978 Jan;204(1):27-38. PMID: 579354.
30	591544	Contrast agent	1977	Morrey BF, O'Brien ET. Femoral neck fractures following water-soluble myelography induced spinal seizures. J Bone Joint Surg Am. 1977 Dec;59(8):1099-100. PMID: 591544.
31	594454	Bismuth	1977	Viterbo VS, Sicard C, Cathala HP. Altérations E.E.G. observées chez 15 malades atteints d'encéphalopathie par les sels de bismuth [E.E.G. changes in 15 patients with bismuth encephalopathy (author's transl)]. Rev Electroencephalogr Neurophysiol Clin. 1977 Apr-Jun;7(2):139-46. French. doi: 10.1016/s0370-4475(77)80070-1. PMID: 594454.
32	611593	Naftidrofuryl	1977	Samson-Dollfus D, Testart J. Observation électroclinique de manifestations comitiales provoquées par l'injection intra-carotidienne de naftidrofuryl [Electrophysiological observations on seizure activity provoked by an intracarotid injection of Naftidrofuryl (author's transl)]. Rev Electroencephalogr Neurophysiol Clin. 1977 Oct-Dec;7(4):459-61. French. doi: 10.1016/s0370-4475(77)80052-x. PMID: 611593.
33	637430	Aluminum	1978	Dunea G, Mahurkar SD, Mamdani B, Smith EC. Role of aluminum in dialysis dementia. Ann Intern Med. 1978 Apr;88(4):502-4. doi: 10.7326/0003-4819-88-4-502. PMID: 637430.
34	638825	Etomidate	1978	Famewo CE, Odugbesan CO. Further experience with etomidate. Can Anaesth Soc J. 1978 Mar;25(2):130-2. doi: 10.1007/BF03005070. PMID: 638825.
35	682330	Gasoline, lead	1978	Hansen KS, Sharp FR. Gasoline sniffing, lead poisoning, and myoclonus. JAMA. 1978 Sep 22;240(13):1375-6. PMID: 682330.
36	716834	Bismuth	1978	Liessens JL, Monstrey J, Vanden Eeckhout E, Djudzman R, Martin JJ. Bismuth encephalopathy. A clinical and anatomo-pathological report of one case. Acta Neurol Belg. 1978 Sep-Oct;78(5):301-9. PMID: 716834.
37	831550	Tricyclic antidepressant	1977	Lippmann S, Moskovitz R, O'Tuama L. Tricyclic-induced myoclonus. Am J Psychiatry. 1977 Jan;134(1):90-1. doi: 10.1176/ajp.134.1.90. PMID: 831550.
38	834718	Bismuth	1977	Grandjean EM. Encéphalopathie associée au traitement par le bismuth: prévention possible? [Bismuth associated myoclonic encephalopathy: is a prevention possible? (author's transl)]. Schweiz Rundsch Med Prax. 1977 Jan 18;66(3):59-61. French. PMID: 834718.
39	848598	Tricyclic antidepressant	1977	Lippmann S, Tucker D, Wagemaker H, Schulte T. A second report of tricyclic-induced mycolonus. Am J Psychiatry. 1977 May;134(5):585-6. PMID: 848598.
40	929035	Bismuth	1977	Buge A, Rancurel G, Dechy H. Encéphalopathies myocloniques bismuthiques. Formes évolutives, complications tardives durables ou définitives. A propos de 41 cas [Bismuth myoclonic encephalopathies. Their course and lasting or definitive late complications]. Rev Neurol (Paris). 1977 Jun-Jul;133(6-7):401-15. French. PMID: 929035.
41	943993	Etomidate	1976	Gooding JM, Corssen G. Etomidate: an ultrashort-acting nonbarbiturate agent for anesthesia induction. Anesth Analg. 1976 Mar-Apr;55(2):286-9. doi: 10.1213/00000539-197603000-00035. PMID: 943993.

42	956618	Bismuth	1976	Quéreux C, Morice J, Level G, Ezes H, Wahl P. L'encéphalopathie bismuthique chez la femme enceinte A propos d'une observation [Bismuth encephalopathy in pregnant women. Apropos of a case]. J Gynecol Obstet Biol Reprod (Paris). 1976 Jan-Feb;5(1):97-103. French. PMID: 956618.
43	961921	Tricyclic antidepressant	1976	Holinger PC, Klawans HL. Reversal of tricyclic-overdosage-induced central anticholinergic syndrome by physostigmine. Am J Psychiatry. 1976 Sep;133(9):1018-23. doi: 10.1176/ajp.133.9.1018. PMID: 961921.
44	976332	Bismuth	1976	Boiteau HL, Cler JM, Mathé JF, Delobel R, Fève J. Relations entre l'évolution des encéphalopathies bismuthiques et les taux de bismuth dans le sang et dans les urines [The relationship between the course of bismuth encephalopathy and the amount of bismuth in blood and urine]. Eur J Toxicol Environ Hyg. 1976 Jul-Aug;9(4):233-9. French. PMID: 976332.
45	980750	Bismuth	1976	Lagueny A, Vallat JM, Julien J. Enregistrement électromyographique au cours des encéphalopathies par les sels de bismuth [Electromyographic recording during encephalopathy caused by bismuth salts]. Nouv Presse Med. 1976 Oct 16;5(34):2252-3. French. PMID: 980750.
46	982067	Bismuth	1976	Mabin D, Bedou G, Tuset MC. Aspects cliniques et électroencéphalographiques de l'encéphalopathie par les sels insolubles de bismuth. A propos de 9 observations [Clinical and electroencephalographic aspects of encephalopathy caused by insoluble bismuth salts. Apropos of 9 cases]. Sem Hop Ther. 1976 Feb;52(2):109-16. French. PMID: 982067.
47	988634	Bismuth	1976	Michaud PA, Regli F. Encéphalopathie au cours des traitements oraux par les sels de bismuth [Encephalopathy during oral treatment with bismuth salts]. Schweiz Med Wochenschr. 1976 Apr 17;106(16):550-5. French. PMID: 988634.
48	996522	Bismuth	1976	Grandjean EM, Ducommun E, Gauthier G, Courvoisier B. L'encéphalopathie myoclonique associée aux sels de bismuth recherche négative d'un élément toxique associé [Myoclonic encephalopathy due to bismuth salts. Negative search for an associated toxic element]. Schweiz Med Wochenschr. 1976 Jul 24;106(30):1006-11. French. PMID: 996522.
49	1007897	Bismuth	1976	Monseu G, Struelens M, Roland M. Bismuth encephalopathy. Acta Neurol Belg. 1976;76(5-6):301-8. PMID: 1007897.
50	1015218	Etomidate	1976	Doom A, Mundeleer P. Etomidate and tonsillectomy. Acta Anaesthesiol Belg. 1976;27 suppl:181-6. PMID: 1015218.
51	1015219	Etomidate	1976	Dubois-Primo J, Bastenier-Geens J, Genicot C, Rucquoi M. A comparative study of etomidate and methohexital as induction agents for analgesic anesthesia. Acta Anaesthesiol Belg. 1976;27 suppl:187-95. PMID: 1015219.
52	1015239	Etomidate	1976	Kay B. Some experience of the use of etomidate in children. Acta Anaesthesiol Belg. 1976;27 suppl:86-92. PMID: 1015239.
53	1079721	Levodopa	1975	Klawans HL, Goetz C, Bergen D. Levodopa-induced myoclonus. Arch Neurol. 1975 May;32(5):330-4. doi: 10.1001/archneur.1975.00490470075011. PMID: 1079721.
54	1082146	5-hydroxytryptophan	1975	Klawans HL, D'Amico DJ, Patel BC. Behavioral supersensitivity to 5-hydroxytryptophan induced by chronic methysergide pretreatment. Psychopharmacologia. 1975 Nov 21;44(3):297-300. doi: 10.1007/BF00428910. PMID: 1082146.
55	1084496	5-hydroxytryptophan	1976	Stewart RM, Growdon JH, Cancian D, Baldessarini RJ. Myoclonus after 5-hydroxytryptophan in rats with lesions of indoleamine neurons in the central nervous system. Neurology. 1976 Jul;26(7):690-2. doi: 10.1212/wnl.26.7.690. PMID: 1084496.
56	1086442	5-hydroxytryptophan	1976	Stewart RM, Growdon JH, Cancian D, Baldessarini RJ. 5-hydroxytryptophan-induced myoclonus: increased sensitivity to serotonin after intracranial 5,7-dihydroxytryptamine in the adult rat. Neuropharmacology. 1976 Aug;15(8):449-55. doi: 10.1016/0028-3908(76)90054-x. PMID: 1086442.
57	1086988	5-hydroxytryptophan	1976	Growdon JH, Young RR, Shahani BT. L-5-hydroxytryptophan in treatment of several different syndromes in which myoclonus is prominent. Neurology. 1976 Dec;26(12):1135-40. doi: 10.1212/wnl.26.12.1135. PMID: 1086988.
58	1088302	5-hydroxytryptophan	1976	van Praag HM, Korf J, Lequin RM. An unexpected effect of L-5 hydroxytryptophan-ethyl-ester combined with a peripheral decarboxylase inhibitor on human serum prolactin. Psychopharmacol Commun. 1976;2(5-6):369-78. PMID: 1088302.
59	1129094	Bismuth	1975	Lhermitte F, Degos CF, Signoret JL. Encéphalopathies réversibles par les sels insolubles de bismuth. Cinq nouveaux cas [Reversible encephalopathies caused by bismuth salts. 5 further cases]. Nouv Presse Med. 1975 Feb 8;4(6):419-20. French. PMID: 1129094.
60	1129100	Bismuth	1975	Tournilhac M, Flori B, Dordain G, Roye JM. Encéphalopathies myocloniques par les sels de bismuth [Letter: Myoclonic encephalopathies caused by bismuth salts]. Nouv Presse Med. 1975 Feb 8;4(6):430. French. PMID: 1129100.
61	1136684	Bismuth	1975	Fressinaud L, Emile J, Charbonnel A, Féve JR. Encéphalopathies myocloniques aiguës résolutives après ingestion de sels de bismuth [Letter: Reversible acute myoclonic encephalopathies after bismuth salt ingestion]. Nouv Presse Med. 1975 Mar 22;4(12):885. French. PMID: 1136684.
62	1142296	Picrotoxin	1975	Marsden CD, Meldrum BS, Pycock C, Tarsy D. Focal myoclonus produced by injection of picrotoxin into the caudate nucleus of the rat. J Physiol. 1975 Mar;246(2):96P. PMID: 1142296.
63	1153289	Bismuth	1975	Barbizet J, Degos JD, Bouchareine A. Le traitement des hypercalcémies Intérêt de la mithramycine [Letter: Encephalopathies caused by bismuth]. Nouv Presse Med. 1975 Apr 26;4(17):1290-1. French. PMID: 1153289.
64	1153303	Bismuth	1975	Goule JP, Husson A, Fondimare A, Rapoport F, Lebreton M, Lajarige V. Encéphalopathie aux sels insolubles de bismuth [Letter: Encephalopathies due to insoluble salts of bismuth]. Nouv Presse Med. 1975 May 3;4(18):1366. French. PMID: 1153303.

65	1164144	Bismuth	1975	Cambier J, Dairou R. L'encéphalopathie myoclonique due a l'ingestion de sels insolubles de bismuth [Myoclonic encephalopathy due to ingestion of insoluble salts of bismuth]. Arch Fr Mal App Dig. 1975 Jan-Feb;64(1):5-9. French. PMID: 1164144.
67	1178476	Bismuth	1975	Boudouresques J, Khalil R, Ali Chérif A, Boudouresques G. Encéphalopathies au bismuth. Un cas avec réaction lymphocytaire du liquide céphalo-rachidien et fiévre prolongée [Letter: Encephalopathies due to bismuth. A case with lymphocytic reaction of cerebrospinal fluid and prolonged fever]. Nouv Presse Med. 1975 Sep 13;4(29):2118 (2). French. PMID: 1178476.
68	1178499	Bismuth	1975	Cambier J, Le Bigot P, Thoyer-Rozat, Irondelle D, Levardon M. Encéphalopathie bismuthique chez une femme enceinte. Naissance d'un enfant normal [Letter: Bismuth induced encephalopathy in a pregnant woman. Birth of a normal child]. Nouv Presse Med. 1975 Sep 27;4(31):2275. French. PMID: 1178499.
69	1178500	Bismuth	1975	Quereux C, Ezes H, Wahl P, Morice J, Level G. Encéphalopathie bismuthique chez la femme enceinte [Letter: Bismuth induced encephalopathy in a pregnant woman]. Nouv Presse Med. 1975 Sep 27;4(31):2275. French. PMID: 1178500.
70	1197982	Bromomethane (methyl bromide)	1975	Goulon M, Nouailhat F, Escourolle R, Zarranz-Imirizaldu JJ, Grosbuis S, Lévy-Alcover MA. Intoxication par le bromure de méthyle Trois observations, dont une mortelle Etude neuro-pathologique d'un cas de stupeur avec myoclonies, suivi pendant cinq ans [Methyl bromide poisoning. 3 cases, 1 fatal. Neruopathological study of one case of coma with myoclonus followed for 5 years]. Rev Neurol (Paris). 1975 Jul;131(7):445-68. French. PMID: 1197982.
71	1220646	Dichloroethane	1975	Dorndorf W, Kresse M, Christain W, Katritzki G. Dichloräthan-Vergiftung mit myoklonischem Syndrom, epileptischen Anfällen und bleibenden cerebralen Defekten [Dichloroethane poisoning with myoclonic syndrome, seizures and irreversible cerebral defects (author's transl)]. Arch Psychiatr Nervenkr (1970). 1975 Dec 22;220(4):373-9. German. doi: 10.1007/BF00342066. PMID: 1220646.
72	1221485	Bismuth	1975	Emile J, Allain P, Truelle JL, Bastard J, Allaert J. Encéphalopathie myoclonique bismuthique après colectomie. Apropos d'un cas [Myoclonic encephalopathy due to bismuth following colectomy. Apropos of a case]. Rev Neurol (Paris). 1975 Nov;131(11):767-74. French. PMID: 1221485.
73	1230924	Bismuth	1975	Hazemann P, Rebelo F, Landau J. Encéphalopathie par le bismuth [Bismuth encephalopathy]. Rev Electroencephalogr Neurophysiol Clin. 1975 Jul-Sep;5(3):291-4. French. doi: 10.1016/s0370-4475(75)80080-3. PMID: 1230924.
74	1230925	Bismuth	1975	Gastaut JL, Tassinari CA, Terzano G, Picornell I. Etude polygraphique de l'encéphalopathie myoclonique bismuthique [Polygraphic study of bismuth-induced myoclonic encephalopathy]. Rev Electroencephalogr Neurophysiol Clin. 1975 Jul-Sep;5(3):295-302. French. doi: 10.1016/s0370-4475(75)80081-5. PMID: 1230925.
75	1232663	Bismuth	1975	Loutre JC, Viguié R, Kunstler E, Nehlil J. Encéphalopathie bismuthique. A propos de deux cas [Bismuth encephalopathy. A propos of 2 cases]. Rev Neurol (Paris). 1975 Dec;131(12):883-6. French. PMID: 1232663.
76	1233892	Bismuth	1975	Besle A, Bussel B, Chapelle JG, Scherrer P. Contribution à l'étude de l'encéphalopathie myoclonique par intoxication bismuthée [Myoclonic encephalopathy due to bismuth poisoning]. Ann Med Psychol (Paris). 1975 Apr;1(4):493-8. French. PMID: 1233892.
77	1239784	Methaqualone	1975	Alpern HP, Greer CA, Stripling JS, Collins AC, Olson RK. Methaqualone: tolerance and physical dependence in mice. Psychopharmacologia. 1975 Nov 21;44(3):303-5. doi: 10.1007/BF00428912. PMID: 1239784.
78	1249582	Bismuth	1976	Loiseau P, Henry P, Jallon P, Legroux M. Encéphalopathies myocloniques iatrogènes aux sels de bismuth [Iatrogenic myoclonic encephalopathies caused by bismuth salts]. J Neurol Sci. 1976 Feb;27(2):133-43. French. doi: 10.1016/0022-510x(76)90056-3. PMID: 1249582.
79	1283011	Hydromorphone	1992	Babul N, Darke AC. Putative role of hydromorphone metabolites in myoclonus. Pain. 1992 Nov;51(2):260-261. doi: 10.1016/0304-3959(92)90270-L. Erratum in: Pain 1993 Jan;52(1):123. PMID: 1283011.
80	1305630	Tryptophan	1992	Sandyk R. L-tryptophan in neuropsychiatric disorders: a review. Int J Neurosci. 1992 Nov-Dec;67(1-4):127-44. doi: 10.3109/00207459208994781. PMID: 1305630.
81	1317656	Tricyclic antidepressants	1992	Lejoyeux M, Rouillon F, Adès J, Gorwood P. Neural symptoms induced by tricyclic antidepressants: phenomenology and pathophysiology. Acta Psychiatr Scand. 1992 Apr;85(4):249-56. doi: 10.1111/j.1600-0447.1992.tb01465.x. PMID: 1317656.
82	1324090	Lithium, pilocarpine	1992	Hirsch E, Baram TZ, Snead OC 3rd. Ontogenic study of lithium-pilocarpine-induced status epilepticus in rats. Brain Res. 1992 Jun 26;583(1-2):120-6. doi: 10.1016/s0006-8993(10)80015-0. PMID: 1324090.
83	1333826	Tricyclic antidepressant	1992	Federico A, Palmeri S, Malandrini A, Mangano L, Ciacci G, Scarpini C, Tiacci G. Dementia, myoclonus, peripheral neuropathy, and lipid-like material in skin biopsy during psychotropic drug treatment. Biol Psychiatry. 1992 Oct 15;32(8):721-7. doi: 10.1016/0006-3223(92)90303-h. PMID: 1333826.
84	1350058	Levodopa	1992	Luquin MR, Scipioni O, Vaamonde J, Gershanik O, Obeso JA. Levodopa-induced dyskinesias in Parkinson's disease: clinical and pharmacological classification. Mov Disord. 1992;7(2):117-24. doi: 10.1002/mds.870070204. PMID: 1350058.
85	1353123	Antipsychotics	1992	Nishimatsu O, Horiguchi J, Inami Y, Innami T, Sasaki A, Kondo K. Nocturnal myoclonus observed in a patient with neuroleptic-induced akathisia. Jpn J Psychiatry Neurol. 1992 Mar;46(1):121-6. doi: 10.1111/j.1440-1819.1992.tb00826.x. PMID: 1353123.
86	1364061	Sultopride	1992	Montaz L, Varache N, Harry P, Aymes C, Turcant A, Delille F, Simonin D, Hass C. Torsades de pointes lors d'une intoxication par sultopride [Torsades de pointes during sultopride poisoning]. J Toxicol Clin Exp. 1992 Dec;12(8):481-6. French. PMID: 1364061.
87	1365651	Abecarnil	1992	Pearce PC, Halsey MJ, MacLean CJ, Passingham S, Pearson J, Mehta RL, Meldrum BS, Jordan CJ, Ward EM. Interactions of the beta carboline abecarnil with the high pressure neurological syndrome in a primate model. Psychopharmacology (Berl). 1992;109(1-2):163-71. doi: 10.1007/BF02245495. PMID: 1365651.
88	1371653	Heroin (diamorphine)	1992	Turner D. Diamorphine toxicity. Anaesthesia. 1992 Feb;47(2):168-9. doi: 10.1111/j.1365-2044.1992.tb02026.x. PMID: 1371653.

89	1373251	Bromomethane (methyl bromide)	1992	Mazzini L, Galante M, Rezzonico M, Kokodoko A. Methylbromide intoxication: a case report. Schweiz Arch Neurol Psychiatr (1985). 1992;143(1):75-80. PMID: 1373251.
90	1376887	Opioids	1992	Eisele JH Jr, Grigsby EJ, Dea G. Clonazepam treatment of myoclonic contractions associated with high-dose opioids: case report. Pain. 1992 May;49(2):231-232. doi: 10.1016/0304-3959(92)90146-3. PMID: 1376887.
91	1410741	Morphine	1992	Franco ML, Berro MJ, Sáez JM, Seoane A. Mioclonías de miembros inferiores como complicación de la morfina espinal [Myoclonus of the legs as a complication of spinal morphine]. Rev Esp Anestesiol Reanim. 1992 May-Jun;39(3):192-3. Spanish. PMID: 1410741.
92	1436817	Diltiazem	1992	Jeret JS, Somasundaram M, Asaikar S. Diltiazem-induced myoclonus. N Y State J Med. 1992 Oct;92(10):447-8. PMID: 1436817.
93	1474184	Desipramine	1992	Masand P. Desipramine-induced oral-pharyngeal disturbances: stuttering and jaw myoclonus. J Clin Psychopharmacol. 1992 Dec;12(6):444-5. doi: 10.1097/00004714-199212000-00014. PMID: 1474184.
94	1497134	Propofol, alfentanil	1992	Kerz T, Jantzen JP. Motorischer Krampfanfall unter Propofol-Alfentanil-Anästhesie? [A myoclonic seizure during propofol-alfentanil anesthesia?]. Anaesthesist. 1992 Jul;41(7):426-30. German. PMID: 1497134.
95	1504799	Gallamine	1992	Burke W, Ramzan I. Myoclonus in the decerebrate cat produced by gallamine. Brain Res. 1992 May 15;580(1-2):189-96. doi: 10.1016/0006-8993(92)90944-5. PMID: 1504799.
96	1517198	Clozapine	1992	Berman I, Zalma A, DuRand CJ, Green AI. Clozapine-induced myoclonic jerks and drop attacks. J Clin Psychiatry. 1992 Sep;53(9):329-30. PMID: 1517198.
97	1527541	Aluminum	1992	Russo LS, Beale G, Sandroni S, Ballinger WE. Aluminium intoxication in undialysed adults with chronic renal failure. J Neurol Neurosurg Psychiatry. 1992 Aug;55(8):697-700. doi: 10.1136/jnnp.55.8.697. PMID: 1527541; PMCID: PMC489208.
98	1540467	Sevoflurane	1992	Adachi M, Ikemoto Y, Kubo K, Takuma C. Seizure-like movements during induction of anaesthesia with sevoflurane. Br J Anaesth. 1992 Feb;68(2):214-5. doi: 10.1093/bja/68.2.214. PMID: 1540467.
99	1557493	Trazodone, buspirone	1992	Goldberg RJ, Huk M. Serotonin syndrome from trazodone and buspirone. Psychosomatics. 1992 Spring;33(2):235-6. doi: 10.1016/S0033-3182(92)72007-6. PMID: 1557493.
100	1562337	Fentanyl	1992	Sprung J, Schedewie HK. Apparent focal motor seizure with a jacksonian march induced by fentanyl: a case report and review of the literature. J Clin Anesth. 1992 Mar-Apr;4(2):139-43. doi: 10.1016/0952-8180(92)90031-u. PMID: 1562337.
101	1596084	Corticotropin-releasing hormone	1992	Baram TZ, Hirsch E, Snead OC 3rd, Schultz L. Corticotropin-releasing hormone-induced seizures in infant rats originate in the amygdala. Ann Neurol. 1992 May;31(5):488-94. doi: 10.1002/ana.410310505. PMID: 1596084; PMCID: PMC3153947.
102	1606085	Busulfan, cyclophosphamide	1992	Meloni G, Raucci U, Pinto RM, Spalice A, Vignetti M, Iannetti P. Pretransplant conditioning with busulfan and cyclophosphamide in acute leukemia patients: neurological and electroencephalographic prospective study. Ann Oncol. 1992 Feb;3(2):145-8. doi: 10.1093/oxfordjournals.annonc.a058131. PMID: 1606085.
103	1710298	Veratramine	1991	Nagata R, Izumi K, Iwata S, Shimizu T, Fukuda T. Mechanisms of veratramine-induced 5-HT syndrome in mice. Jpn J Pharmacol. 1991 Jan;55(1):139-46. doi: 10.1254/jjp.55.139. PMID: 1710298.
104	1711699	Ro5-4864, lindane, picrotoxin	1991	Drummer HL, Woolley DE. Toxicokinetics of Ro 5-4864, lindane and picrotoxin compared. Pharmacol Biochem Behav. 1991 Feb;38(2):235-42. doi: 10.1016/0091-3057(91)90271-3. PMID: 1711699.
105	1736182	Levodopa	1992	Neufeld MY. Periodic triphasic waves in levodopa-induced encephalopathy. Neurology. 1992 Feb;42(2):444-6. doi: 10.1212/wnl.42.2.444. PMID: 1736182.
106	1768819	Etomidate	1991	Ford SR, Maze M, Gaba DM. A comparison of etomidate and thiopental anesthesia for cardioversion. J Cardiothorac Vasc Anesth. 1991 Dec;5(6):563-5. doi: 10.1016/1053-0770(91)90006-f. PMID: 1768819.
107	1768820	Etomidate	1991	Canessa R, Lema G, Urzúa J, Dagnino J, Concha M. Anesthesia for elective cardioversion: a comparison of four anesthetic agents. J Cardiothorac Vasc Anesth. 1991 Dec;5(6):566-8. doi: 10.1016/1053-0770(91)90007-g. PMID: 1768820.
108	1795756	Mefloquine	1991	Besser R, Krämer G. Verdacht auf anfallfördernde Wirkung von Mefloquin (Lariam) [Suspected convulsive side-effect of mefloquine (Lariam)]. Nervenarzt. 1991 Dec;62(12):760-1. German. PMID: 1795756.
109	1883778	Ganglion block	1991	Watanabe S, Tanaka M, Endo T. Myoclonus of hand and arm after ipsilateral stellate ganglion block. Reg Anesth. 1991 May-Jun;16(3):186-7. PMID: 1883778.
110	1900790	Carbamazepine	1991	Dhuna A, Pascual-Leone A, Talwar D. Exacerbation of partial seizures and onset of nonepileptic myoclonus with carbamazepine. Epilepsia. 1991 Mar-Apr;32(2):275-8. doi: 10.1111/j.1528-1157.1991.tb05255.x. PMID: 1900790.
111	1903661	Psychotropic	1991	Förstl H, Zagorski A, Pohlmann-Eden B. Somatosensory evoked potentials as indicators of altered cerebral excitability during psychotropic drug treatment. Biol Psychiatry. 1991 Feb 15;29(4):397-402. doi: 10.1016/0006-3223(91)90226-c. PMID: 1903661.
112	1904172	Diazepam, ketamine, midazolam	1991	Hellyer PW, Freeman LC, Hubbell JA. Induction of anesthesia with diazepam-ketamine and midazolam-ketamine in greyhounds. Vet Surg. 1991 Mar-Apr;20(2):143-7. doi: 10.1111/j.1532-950x.1991.tb00324.x. PMID: 1904172.

113	1923920	Ceftazidime	1991	Slaker RA, Danielson B. Neurotoxicity associated with ceftazidime therapy in geriatric patients with renal dysfunction. Pharmacotherapy. 1991;11(4):351-2. PMID: 1923920.
114	1958958	Serotonin reuptake inhibitors	1991	Deahl M, Trimble M. Serotonin reuptake inhibitors, epilepsy and myoclonus. Br J Psychiatry. 1991 Sep;159:433-5. doi: 10.1192/bjp.159.3.433. PMID: 1958958.
115	1965204	Epinephrine	1990	Uchihara T, Ichikawa T, Furukawa T, Tsukagoshi H. Myoclonus with burning sensation in legs that remits with sympathetic blockade. J Neurol Sci. 1990 Dec;100(1-2):161-4. doi: 10.1016/0022-510x(90)90028-l. PMID: 1965204.
116	1971187	Antipsychotics	1990	Fukuzako H, Tominaga H, Izumi K, Koja T, Nomoto M, Hokazono Y, Kamei K, Fujii H, Fukuda T, Matsumoto K. Postural myoclonus associated with long-term administration of neuroleptics in schizophrenic patients. Biol Psychiatry. 1990 May 15;27(10):1116-26. doi: 10.1016/0006-3223(90)90048-7. PMID: 1971187.
117	2023778	Epidural anesthesia	1991	Hachisuka K, Ogata H, Kohshi K. Post-operative paraplegia with spinal myoclonus possibly caused by epidural anaesthesia: case report. Paraplegia. 1991 Feb;29(2):131-6. doi: 10.1038/sc.1991.18. PMID: 2023778.
118	2048668	Heroin (diamorphine)	1991	Jayawardena B, Hill DJ. Myoclonic spasms after epidural diamorphine infusion. Anaesthesia. 1991 Jun;46(6):473-4. doi: 10.1111/j.1365-2044.1991.tb11688.x. PMID: 2048668.
119	2087534	Veratramine	1990	Fukuda T. [Veratramine-induced myoclonus]. Seishin Shinkeigaku Zasshi. 1990;92(11):865-9. Japanese. PMID: 2087534.
120	2087659	Pentostatin (deoxycoformycin)	1990	Escoda L, López-Guillermo A, Formigón M, Estrach T, Cervantes F, Montserrat E, Rozman C. Tratamiento de diferentes síndromes linfoproliferativos con deoxicoformicina. Resultados en 6 pacientes [Treatment of various lymphoproliferative syndromes with deoxycoformycin: results in 6 patients]. Sangre (Barc). 1990 Dec;35(6):421-4. Spanish. PMID: 2087659.
121	2090022	DDT	1990	Rabasa M, Tabernero C, Pardo B, García de Yébenes J. Efecto de los antagonistas de los canales de calcio sobre las mioclonías inducidas por DDT [The effect of calcium channel blockers on myoclonus induced by DDT]. Arch Neurobiol (Madr). 1990 Jul-Aug;53(4):176-84. Spanish. Erratum in: Arch Neurobiol (Madr) 1990 Sep-Oct;53(5):following 204. PMID: 2090022.
122	2147669	Picrotoxin	1990	Paul V, Krishnamoorthy MS. A functional interaction between GABA and 5-HT in inhibiting picrotoxin-induced myoclonus in rats. Indian J Physiol Pharmacol. 1990 Apr;34(2):139-42. PMID: 2147669.
123	2181694	Tricyclic antidepressant	1990	Patterson JF. Myoclonus caused by a tricyclic antidepressant. South Med J. 1990 Apr;83(4):463-5. doi: 10.1097/00007611-199004000-00026. PMID: 2181694.
124	2207824	Etomidate	1990	Mitterschiffthaler G, Lechleitner P, Hauptlorenz S, Wencker M, Dienstl F. Anesthésie pour cardioversion. Comparaison du propofol et de l'étomidate [Anesthesia for cardioversion. A comparison of propofol and etomidate]. Cah Anesthesiol. 1990 May-Jun;38(3):159-63. French. PMID: 2207824.
125	2294817	Bismuth	1990	Mendelowitz PC, Hoffman RS, Weber S. Bismuth absorption and myoclonic encephalopathy during bismuth subsalicylate therapy. Ann Intern Med. 1990 Jan 15;112(2):140-1. doi: 10.7326/0003-4819-112-2-140. PMID: 2294817.
126	2309508	Ciprofloxacin	1990	Schwartz MT, Calvert JF. Potential neurologic toxicity related to ciprofloxacin. DICP. 1990 Feb;24(2):138-40. doi: 10.1177/106002809002400204. PMID: 2309508.
127	2327237	Bromomethane (methyl bromide)	1990	Uncini A, Basciani M, Di Muzio A, Antonini D, Onofrj M. Methyl bromide myoclonus: an electrophysiological study. Acta Neurol Scand. 1990 Feb;81(2):159-64. doi: 10.1111/j.1600-0404.1990.tb00953.x. PMID: 2327237.
128	2333138	Contrast agent (meglumine diatrizoate)	1990	Hilz MJ, Huk W, Schellmann B, Sörgel F, Druschky KF. Fatal complications after myelography with meglumine diatrizoate. Neuroradiology. 1990;32(1):70-3. doi: 10.1007/BF00593948. PMID: 2333138.
129	2347607	Picrotoxin	1990	Paul V, Krishnamoorthy MS. The synergistic protective effect of propranolol & aminooxyacetic acid against picrotoxin-induced myoclonus in rats. Indian J Med Res. 1990 Feb;92:17-20. PMID: 2347607.
130	2347858	Fluoxetine, MAOI	1990	Feighner JP, Boyer WF, Tyler DL, Neborsky RJ. Adverse consequences of fluoxetine-MAOI combination therapy. J Clin Psychiatry. 1990 Jun;51(6):222-5. PMID: 2347858.
131	2353331	Propafenone	1990	Jeandel C, Bannwarth B, Trechot P, Barroche G, Penin F, Cuny G. Encéphalopathie myoclonique à la propafénone [Myoclonic encephalopathy caused by propafenone]. Therapie. 1990 Mar-Apr;45(2):161-2. French. PMID: 2353331.
132	2356356	Bismuth	1990	Nogué Xarau S, Sanz Gallén P. Encefalopatía mioclónica por bismuto [Myoclonic encephalopathy caused by bismuth]. Rev Clin Esp. 1990 Feb;186(3):145. Spanish. PMID: 2356356.
133	2412143	Lindane, dieldrin	1985	Albertson TE, Joy RM, Stark LG. Chlorinated hydrocarbon pesticides and amygdaloid kindling. Neurobehav Toxicol Teratol. 1985 May-Jun;7(3):233-7. PMID: 2412143.
134	2418651	DDT	1986	Pratt JA, Rothwell J, Jenner P, Marsden CD. p,p'-DDT-induced myoclonus in the rat and its application as an animal model of 5-HT-sensitive action myoclonus. Adv Neurol. 1986;43:577-88. PMID: 2418651.
135	2475196	Morphine	1989	Potter JM, Reid DB, Shaw RJ, Hackett P, Hickman PE. Myoclonus associated with treatment with high doses of morphine: the role of supplemental drugs. BMJ. 1989 Jul 15;299(6692):150-3. doi: 10.1136/bmj.299.6692.150. PMID: 2475196; PMCID: PMC1837058.
136	2488054	Clozapine, lithium	1989	Lemus CZ, Lieberman JA, Johns CA. Myoclonus during treatment with clozapine and lithium: the role of serotonin. Hillside J Clin Psychiatry. 1989;11(2):127-30. PMID: 2488054.

137	2502002	Contrast agent	1989	Heyman SN, Greenfeld Z, Behar M, Mogle P, Brezis M. Spinal myoclonus induced by contrast material: an alternative explanation. AJNR Am J Neuroradiol. 1989 May-Jun;10(3):653. PMID: 2502002; PMCID: PMC8334515.
138	2507405	Carbamazepine	1989	Zucconi M, Coccagna G, Petronelli R, Gerardi R, Mondini S, Cirignotta F. Nocturnal myoclonus in restless legs syndrome effect of carbamazepine treatment. Funct Neurol. 1989 Jul-Sep;4(3):263-71. PMID: 2507405.
139	2508871	Morphine	1989	Myoclonus associated with high doses of morphine. BMJ. 1989 Sep 9;299(6700):683-4. doi: 10.1136/bmj.299.6700.683-c. PMID: 2508871; PMCID: PMC1837587.
140	2568430	Pentetrazol	1989	Escorihuela RM, Boix F, Corda MG, Tobeña A, Fernández Teruel A. Chronic but not acute antidepressant treatment increases pentetrazol-induced convulsions in mice. J Pharm Pharmacol. 1989 Feb;41(2):143-4. doi: 10.1111/j.2042-7158.1989.tb06416.x. PMID: 2568430.
141	2572009	DDT	1989	Truong DD, Galloway MP, Pezzoli G, Jamrozik Z, Fahn S. Milacemide increases 5-hydroxytryptamine and dopamine levels in rat brain--possible mechanisms of milacemide antimyoclonic property in the p,p'-DDT-induced myoclonus. Pharmacol Biochem Behav. 1989 Apr;32(4):993-1001. doi: 10.1016/0091-3057(89)90072-5. PMID: 2572009.
142	2579521	Morphine	1985	Shohami E, Evron S. Intrathecal morphine induces myoclonic seizures in the rat. Acta Pharmacol Toxicol (Copenh). 1985 Jan;56(1):50-4. doi: 10.1111/j.1600-0773.1985.tb01252.x. PMID: 2579521.
143	2609846	Buflomedil	1989	Lucas C, Soetaert G, Leys D, Petit H. Myoclonies au cours d'un traitement par buflomédil [Myoclonus during a course with buflomedil treatment]. Acta Clin Belg. 1989;44(5):360-1. French. doi: 10.1080/17843286.1989.11718042. PMID: 2609846.
144	2614455	Cocaine	1989	Scharf D. Opsoclonus-myoclonus following the intranasal usage of cocaine. J Neurol Neurosurg Psychiatry. 1989 Dec;52(12):1447-8. doi: 10.1136/jnnp.52.12.1447-a. PMID: 2614455; PMCID: PMC1031619.
145	2614458	Norpethidine	1989	Reutens DC, Stewart-Wynne EG. Norpethidine induced myoclonus in a patient with renal failure. J Neurol Neurosurg Psychiatry. 1989 Dec;52(12):1450-1. doi: 10.1136/jnnp.52.12.1450. PMID: 2614458; PMCID: PMC1031622.
146	2637391	Tricyclic antidepressant	1989	Fukuzako H, Hokazono Y, Tominaga H, Hirakawa K, Matsumoto K. Jerk-locked averaging and somatosensory evoked potential in tricyclic-induced myoclonus: a case report. Jpn J Psychiatry Neurol. 1989 Dec;43(4):645-9. doi: 10.1111/j.1440-1819.1989.tb03100.x. PMID: 2637391.
147	2722635	Etomidate	1989	Muir WW 3rd, Mason DE. Side effects of etomidate in dogs. J Am Vet Med Assoc. 1989 May 15;194(10):1430-4. PMID: 2722635.
148	2740555	Bismuth	1989	Gómez Jiménez J, Alvarez J, Boada Rovira M, Codina-Puiggros A. Encelafopatía mioclónica por bismuto [Myoclonic encephalopathy caused by bismuth]. Rev Clin Esp. 1989 Mar;184(4):212. Spanish. PMID: 2740555.
149	2748860	LSD	1989	Carvey P, Nausieda P, Weertz R, Klawans H. LSD and other related hallucinogens elicit myoclonic jumping behavior in the guinea pig. Prog Neuropsychopharmacol Biol Psychiatry. 1989;13(1-2):199-210. doi: 10.1016/0278-5846(89)90017-1. PMID: 2748860.
150	2764296	Etomidate	1989	Berry JM, Merin RG. Etomidate myoclonus and the open globe. Anesth Analg. 1989 Aug;69(2):256-9. PMID: 2764296.
151	2770373	Bismuth	1989	Molina JA, Calandre L, Bermejo F, Posadas F, Fernández-Ortega JD. Encefalopatía mioclónica por sales de bismuto. Eficacia del tratamiento con dimercaprol [Myoclonic encephalopathy caused by bismuth salts. Efficacy of treatment with dimercaprol]. Med Clin (Barc). 1989 Jun 3;93(1):20-2. Spanish. PMID: 2770373.
152	2787127	Etomidate	1989	Gillies GW, Lees NW. The effects of speed of injection on induction with propofol. A comparison with etomidate. Anaesthesia. 1989 May;44(5):386-8. doi: 10.1111/j.1365-2044.1989.tb11333.x. PMID: 2787127.
153	2896777	Alphaxalone	1988	File SE, Simmonds MA. Myoclonic seizures in the mouse induced by alphaxalone and related steroid anaesthetics. J Pharm Pharmacol. 1988 Jan;40(1):57-9. doi: 10.1111/j.2042-7158.1988.tb05152.x. PMID: 2896777.
154	2913801	Aluminum	1989	Kirschbaum BB, Schoolwerth AC. Acute aluminum toxicity associated with oral citrate and aluminum-containing antacids. Am J Med Sci. 1989 Jan;297(1):9-11. doi: 10.1097/00000441-198901000-00003. PMID: 2913801.
155	2915770	Nifedipine	1989	Pedro-Botet ML, Bonal J, Caralps A. Nifedipine and myoclonic disorders. Nephron. 1989;51(2):281. doi: 10.1159/000185303. PMID: 2915770.
156	2938164	Propafenone	1986	Devoize JL, Flammang D, Marcombes JL. Encéphalopathie myoclonique probablement due à la propafénone [Myoclonic encephalopathy probably due to propafenone]. Presse Med. 1986 Feb 22;15(8):398. French. PMID: 2938164.
157	2943526	Floxuridine	1986	Taylor HG, Wolf CR, Maitland CG. Neurologic toxicity associated with hepatic artery infusion HAI of FUdR. Cancer Chemother Pharmacol. 1986;17(3):292-3. doi: 10.1007/BF00256703. PMID: 2943526.
158	2955328	Bismuth	1987	Vidailhet M, Le TH, Wechsler B, Godeau P. L'encéphalopathie au bismuth n'a pas disparu.. [Encephalopathy caused by bismuth has not disappeared...]. Presse Med. 1987 Jun 6;16(21):1054. French. PMID: 2955328.
159	2959172	Sufentanil	1987	Bowdle TA. Myoclonus following sufentanil without EEG seizure activity. Anesthesiology. 1987 Oct;67(4):593-5. doi: 10.1097/00000542-198710000-00030. PMID: 2959172.
160	2965366	Propafenone	1988	Madigand M, Dien J, Pavin G, Allain H. Encéphalopathie myoclonique probablement imputable à la propafénone [Myoclonic encephalopathy probably attributable to propafenone]. Presse Med. 1988 Mar 26;17(11):538. French. PMID: 2965366.

161	2979066	Etomidate	1987	Stockham RJ, Stanley TH, Pace NL, King K, Groen F, Gillmor ST. Induction of anesthesia with fentanyl or fentanyl plus etomidate in high-risk patients. J Cardiothorac Anesth. 1987 Feb;1(1):19-23. doi: 10.1016/s0888-6296(87)92558-0. PMID: 2979066.
162	2983163	Urea	1985	Chung E, Yocca F, Van Woert MH. Urea-induced myoclonus: medullary glycine antagonism as mechanism of action. Life Sci. 1985 Mar 18;36(11):1051-8. doi: 10.1016/0024-3205(85)90490-4. PMID: 2983163.
163	2985188	5-hydroxytryptophan	1985	O'Connor LH, Feder HH. Estradiol and progesterone influence L-5-hydroxytryptophan-induced myoclonus in male guinea pigs: sex differences in serotonin-steroid interactions. Brain Res. 1985 Mar 18;330(1):121-5. doi: 10.1016/0006-8993(85)90012-5. PMID: 2985188.
164	2986413	DDT	1985	Magnussen I. p,p'-DDT-induced myoclonus in mice: the effect of enhanced 5-HT neurotransmission. Acta Pharmacol Toxicol (Copenh). 1985 Feb;56(2):87-90. doi: 10.1111/j.1600-0773.1985.tb01258.x. PMID: 2986413.
165	3004126	Urea	1986	Chung EY, Van Woert MH. Urea myoclonus: possible involvement of glycine. Adv Neurol. 1986;43:565-8. PMID: 3004126.
166	3052168	Etomidate	1988	Ulsamer B, Raps M. Narkoseeinleitung mit Propofol im Vergleich zu Etomidat [Induction of anesthesia using propofol in comparison with etomidate]. Anaesthesist. 1988 Aug;37(8):517-21. German. PMID: 3052168.
167	3089048	Etomidate	1986	Collin RI, Drummond GB, Spence AA. Alfentanil supplemented anaesthesia for short procedures. A double-blind study of alfentanil used with etomidate and enflurane for day cases. Anaesthesia. 1986 May;41(5):477-81. doi: 10.1111/j.1365-2044.1986.tb13270.x. PMID: 3089048.
168	3110644	Baclofen	1987	Cottrell GA, Robertson HA. Baclofen exacerbates epileptic myoclonus in kindled rats. Neuropharmacology. 1987 Jun;26(6):645-8. doi: 10.1016/0028-3908(87)90161-4. PMID: 3110644.
169	3117123	Lithium	1987	Julius SC, Brenner RP. Myoclonic seizures with lithium. Biol Psychiatry. 1987 Oct;22(10):1184-90. doi: 10.1016/0006-3223(87)90026-6. PMID: 3117123.
170	3148641	Tricyclic antidepressant, lithium	1988	Devanand DP, Sackeim HA, Brown RP. Myoclonus during combined tricyclic antidepressant and lithium treatment. J Clin Psychopharmacol. 1988 Dec;8(6):446-7. doi: 10.1097/00004714-198812000-00024. PMID: 3148641.
171	3157120	Buflomedil	1985	Otmane-Telba M, Gury B, Paulien R, Feret J, Nouailhat F. Toxicité neurologique réversible du surdosage au buflomédil [Reversible neurologic toxicity of buflomedil overdosage]. Presse Med. 1985 Feb 9;14(5):286. French. PMID: 3157120.
172	3173367	DDT	1988	Truong DD, Garcia De Yebenes J, Pezzoli G, Jackson-Lewis V, Fahn S. Glycine involvement in DDT-induced myoclonus. Mov Disord. 1988;3(1):77-87. doi: 10.1002/mds.870030110. PMID: 3173367.
173	3204387	Metoclopramide	1988	Lu CS, Chu NS. Acute dystonic reaction with asterixis and myoclonus following metoclopramide therapy. J Neurol Neurosurg Psychiatry. 1988 Jul;51(7):1002-3. doi: 10.1136/jnnp.51.7.1002-a. PMID: 3204387; PMCID: PMC1033214.
174	3233864	Cefmetazole	1988	Uchihara T, Tsukagoshi H. Myoclonic activity associated with cefmetazole, with a review of neurotoxicity of cephalosporins. Clin Neurol Neurosurg. 1988;90(4):369-71. doi: 10.1016/0303-8467(88)90013-3. PMID: 3233864.
175	3244442	Bismuth	1988	Ross JF, Sahenk Z, Hyser C, Mendell JR, Alden CL. Characterization of a murine model for human bismuth encephalopathy. Neurotoxicology. 1988 Winter;9(4):581-6. PMID: 3244442.
176	3316313	Antipsychotic	1987	Kramer LD, Locke GE. A case of neuroleptic malignant syndrome with myoclonus and triphasic EEG waves. J Clin Psychopharmacol. 1987 Oct;7(5):354-6. PMID: 3316313.
177	3348460	Glutethimide	1988	Bauer MS, Fus AF, Hanich RF, Ross RJ. Glutethimide intoxication and withdrawal. Am J Psychiatry. 1988 Apr;145(4):530-1. doi: 10.1176/ajp.145.4.530b. PMID: 3348460.
178	3354919	Etomidate	1988	Shulman MS, Edelmann R. Use of etomidate for elective cardioversion. Anesthesiology. 1988 Apr;68(4):656. doi: 10.1097/00000542-198804000-00055. PMID: 3354919.
179	3372713	Trazodone	1988	Patel HC, Bruza D, Yeragani V. Myoclonus with trazodone. J Clin Psychopharmacol. 1988 Apr;8(2):152. doi: 10.1097/00004714-198804000-00026. PMID: 3372713.
180	3379031	Buspirone	1988	Ritchie EC, Bridenbaugh RH, Jabbari B. Acute generalized myoclonus following buspirone administration. J Clin Psychiatry. 1988 Jun;49(6):242-3. PMID: 3379031.
181	3379388	MAOI	1988	Askenasy JJ, Yahr MD. Is monoamine oxidase inhibitor induced myoclonus serotoninergically mediated? J Neural Transm. 1988;72(1):67-76. doi: 10.1007/BF01244633. PMID: 3379388.
182	3394909	Etomidate	1988	Stockham RJ, Stanley TH, Pace NL, Gillmor S, Groen F, Hilkens P. Fentanyl pretreatment modifies anaesthetic induction with etomidate. Anaesth Intensive Care. 1988 May;16(2):171-6. doi: 10.1177/0310057X8801600207. PMID: 3394909.
183	3400849	Morphine	1988	Glavina MJ, Robertshaw R. Myoclonic spasms following intrathecal morphine. Anaesthesia. 1988 May;43(5):389-90. doi: 10.1111/j.1365-2044.1988.tb09020.x. PMID: 3400849.
184	3401861	Levodopa	1988	Hirschorn KA, Greenberg HS. Successful treatment of levodopa-induced myoclonus and levodopa withdrawal-induced neuroleptic malignant syndrome. A case report. Clin Neuropharmacol. 1988 Jun;11(3):278-81. doi: 10.1097/00002826-198806000-00011. PMID: 3401861.

185	3405506	Metoclopramide	1988	Eisele G. Neurologic complications of metoclopramide therapy. N Y State J Med. 1988 Jun;88(6):332. PMID: 3405506.
186	3433813	Carbon monoxide	1987	Kim JS, Lee SA, Kim JS. Myoclonus, delayed sequelae of carbon monoxide poisoning, piracetam trial. Yonsei Med J. 1987;28(3):231-3. doi: 10.3349/ymj.1987.28.3.231. PMID: 3433813.
187	3494632	Ketanserin	1987	Bo P, Patrucco M, Savoldi F. Neuropharmacological profile of ketanserin. Farmaco Sci. 1987 Feb;42(2):91-9. PMID: 3494632.
188	3519655	Bromomethane (methyl bromide)	1986	Minami M, Hirata Y. [Methyl bromide poisoning]. Nihon Ika Daigaku Zasshi. 1986 Apr;53(2):129-36. Japanese. doi: 10.1272/jnms1923.53.129. PMID: 3519655.
189	3536383	Alprazolam	1986	Browne JL, Hauge KJ. A review of alprazolam withdrawal. Drug Intell Clin Pharm. 1986 Nov;20(11):837-41. doi: 10.1177/106002808602001102. PMID: 3536383.
190	3574698	Bromocriptine	1987	Buchman AS, Bennett DA, Goetz CG. Bromocriptine-induced myoclonus. Neurology. 1987 May;37(5):885. doi: 10.1212/wnl.37.5.885. PMID: 3574698.
191	3587612	Picrotoxin	1987	Patel S, Slater P. Analysis of the brain regions involved in myoclonus produced by intracerebral picrotoxin. Neuroscience. 1987 Feb;20(2):687-93. doi: 10.1016/0306-4522(87)90119-9. PMID: 3587612.
192	3607450	Cysteamine	1987	Cottrell GA, Robertson HA. Prevention of cysteamine-induced myoclonus blocks the long-term inhibition of kindled seizures. Brain Res. 1987 May 26;412(1):161-4. doi: 10.1016/0006-8993(87)91453-3. PMID: 3607450.
193	3611037	Phenelzine	1987	White PD. Myoclonus and episodic delirium associated with phenelzine: a case report. J Clin Psychiatry. 1987 Aug;48(8):340-1. PMID: 3611037.
194	3621016	Etomidate	1987	Fassoulaki A, Pateras C, Kaniaris P. Le fentanyl dans la prévention des myoclonies dues à l'étomidate [Fentanyl in the prevention of etomidate-induced myoclonus]. Cah Anesthesiol. 1987 May-Jun;35(3):201-2. French. PMID: 3621016.
195	3631565	Spinal anesthesia	1987	Watanabe S, Sakai K, Ono Y, Seino H, Naito H. Alternating periodic leg movement induced by spinal anesthesia in an elderly male. Anesth Analg. 1987 Oct;66(10):1031-2. Erratum in: Anesth Analg 1988 Feb;67(2):204. PMID: 3631565.
196	3653053	Carbamazepine	1987	Aguglia U, Zappia M, Quattrone A. Carbamazepine-induced nonepileptic myoclonus in a child with benign epilepsy. Epilepsia. 1987 Sep-Oct;28(5):515-8. doi: 10.1111/j.1528-1157.1987.tb03680.x. PMID: 3653053.
197	3659502	Etomidate	1987	Castillo Monsegur J, Villalonga Morales A, Nalda Felipe MA. Prevención farmacológica de las mioclonías durante la inducción anestésica con etomidato. Estudio comparativo entre fentanil, flunitracepam y pancuronio [Pharmacological prevention of myoclonias during anesthesia induction with etomidate. Comparative study of fentanyl, flunitrazepam and pancuronium]. Rev Esp Anestesiol Reanim. 1987 Jul-Aug;34(4):270-2. Spanish. PMID: 3659502.
198	3690996	Insect-repellent	1987	Edwards DL, Johnson CE. Insect-repellent-induced toxic encephalopathy in a child. Clin Pharm. 1987 Jun;6(6):496-8. PMID: 3690996.
199	3693873	Clomipramine	1987	Casas M, Garcia-Ribera C, Alvarez E, Udina C, Queralto JM, Grau JM. Myoclonic movements as a side-effect of treatment with therapeutic doses of clomipramine. Int Clin Psychopharmacol. 1987 Oct;2(4):333-6. doi: 10.1097/00004850-198710000-00006. PMID: 3693873.
200	3711372	Levodopa	1986	Sandyk R. L-dopa induced "serotonin syndrome" in a parkinsonian patient on bromocriptine. J Clin Psychopharmacol. 1986 Jun;6(3):194-5. doi: 10.1097/00004714-198606000-00022. PMID: 3711372.
201	3729352	Vidarabine	1986	Vilter RW. Vidarabine-associated encephalopathy and myoclonus. Antimicrob Agents Chemother. 1986 May;29(5):933-5. doi: 10.1128/AAC.29.5.933. PMID: 3729352; PMCID: PMC284185.
202	3738683	Aluminum	1986	Ragni R. L'intossicazione de alluminio nella insufficienza renale cronica [Aluminum poisoning in chronic renal insufficiency]. Minerva Urol Nefrol. 1986 Jan-Mar;38(1):87-95. Italian. PMID: 3738683.
203	3752305	Lithium	1986	Heiman EM, Christie M. Lithium-aggravated nocturnal myoclonus and restless legs syndrome. Am J Psychiatry. 1986 Sep;143(9):1191-2. doi: 10.1176/ajp.143.9.1191b. PMID: 3752305.
204	3752481	Etomidate	1986	Kochs E, Treede RD, Schulte am Esch J. Vergrösserung somatosensorisch evozierter Potentiale während Narkoseeinleitung mit Etomidat [Increase in somatosensory evoked potentials during anesthesia induction with etomidate]. Anaesthesist. 1986 Jun;35(6):359-64. German. PMID: 3752481.
205	3767553	Deferoxamine	1986	Sprague SM, Corwin HL, Wilson RS, Mayor GH, Tanner CM. Encephalopathy in chronic renal failure responsive to deferoxamine therapy. Another manifestation of aluminum neurotoxicity. Arch Intern Med. 1986 Oct;146(10):2063-4. PMID: 3767553.
206	3772334	Bromomethane (methyl bromide)	1986	Prockop LD, Smith AO. Seizures and action myoclonus after occupational exposure to methyl bromide. J Fla Med Assoc. 1986 Sep;73(9):690-2. PMID: 3772334.
207	3777258	Clomipramine	1986	Myers BA, Klerman GL, Hartmann E. Nocturnal cataclysms with myoclonus: a new side effect of clomipramine. Am J Psychiatry. 1986 Nov;143(11):1490-1. doi: 10.1176/ajp.143.11.1490b. PMID: 3777258.
208	3806075	Phenelzine, alprazolam	1987	Naylor MW, Grunhaus L, Cameron O. Myoclonic seizures after abrupt withdrawal from phenelzine and alprazolam. J Nerv Ment Dis. 1987 Feb;175(2):111-4. doi: 10.1097/00005053-198702000-00008. PMID: 3806075.

209	3808321	Physostigmine	1987	Mayeux R, Albert M, Jenike M. Physostigmine-induced myoclonus in Alzheimer's disease. Neurology. 1987 Feb;37(2):345-6. doi: 10.1212/wnl.37.2.345. PMID: 3808321.
210	3827519	Tricyclic antidepressant	1987	Garvey MJ, Tollefson GD. Occurrence of myoclonus in patients treated with cyclic antidepressants. Arch Gen Psychiatry. 1987 Mar;44(3):269-72. doi: 10.1001/archpsyc.1987.01800150081010. PMID: 3827519.
211	3889518	Bismuth	1985	Sanz Gallén P, Nogué Xarau S. Intoxicación por sales de bismuto [Poisoning by bismuth salts]. Med Clin (Barc). 1985 Apr 6;84(13):538-41. Spanish. PMID: 3889518.
212	3946111	Bromomethane (methyl bromide)	1986	Hauw JJ, Escourolle R, Baulac M, Morel-Maroger A, Goulon M, Castaigne P. Postmortem studies on posthypoxic and post-methyl bromide intoxication: case reports. Adv Neurol. 1986;43:201-14. PMID: 3946111.
213	3946117	Urea	1986	Muscatt S, Rothwell J, Obeso J, Leigh N, Jenner P, Marsden CD. Urea-induced stimulus-sensitive myoclonus in the rat. Adv Neurol. 1986;43:553-63. PMID: 3946117.
214	3946118	DDT	1986	Chung EY, Van Woert MH. DDT myoclonus: site of "myoclonus center" in the brain. Adv Neurol. 1986;43:569-75. PMID: 3946118.
215	3948001	Cysteamine	1986	Cottrell GA, Robertson HA. Induction and suppression of seizures by cysteamine in hippocampal kindled rats. Brain Res. 1986 Feb 19;365(2):393-6. doi: 10.1016/0006-8993(86)91657-4. PMID: 3948001.
216	3956381	Moxalactam	1986	Cho I, Bertoni JM, Hopkins L. Moxalactam myoclonus, seizures, and encephalopathy. Drug Intell Clin Pharm. 1986 Mar;20(3):223-4. doi: 10.1177/106002808602000310. PMID: 3956381.
217	3957629	Carbamazepine	1986	Tartara A, Manni R, Maurelli M, Sandrini G, Savoldi F. Carbamazepine poisoning: a case report. Ital J Neurol Sci. 1986 Feb;7(1):165-6. doi: 10.1007/BF02230436. PMID: 3957629.
218	3966657	Etomidate	1985	Laughlin TP, Newberg LA. Prolonged myoclonus after etomidate anesthesia. Anesth Analg. 1985 Jan;64(1):80-2. PMID: 3966657.
219	3976924	Monoamine oxidase inhibitors, tryptophan	1985	Pope HG Jr, Jonas JM, Hudson JI, Kafka MP. Toxic reactions to the combination of monoamine oxidase inhibitors and tryptophan. Am J Psychiatry. 1985 Apr;142(4):491-2. doi: 10.1176/ajp.142.4.491. PMID: 3976924.
220	3979053	Aluminum	1985	Bates D, Parkinson IM, Ward MK, Kerr DN. Aluminium encephalopathy. Contrib Nephrol. 1985;45:29-41. doi: 10.1159/000410446. PMID: 3979053.
221	3999823	Bismuth	1985	Nogué S, Mas A, Parés A, Nadal P, Bertrán A, Torra M, Bachs M, Blesa R. Encefalopatía mioclónica y convulsiva por bismuto. Utilidad del tratamiento con dimercaprol [Myoclonic and convulsive encephalopathy caused by bismuth. Usefulness of dimercaprol treatment]. Med Clin (Barc). 1985 Apr 6;84(13):530-2. Spanish. PMID: 3999823.
222	4003975	Verapamil	1985	Hicks CB, Abraham K. Verapamil and myoclonic dystonia. Ann Intern Med. 1985 Jul;103(1):154. doi: 10.7326/0003-4819-103-1-154_1. PMID: 4003975.
223	4003975	Verapamil	1985	Hicks CB, Abraham K. Verapamil and myoclonic dystonia. Ann Intern Med. 1985 Jul;103(1):154. doi: 10.7326/0003-4819-103-1-154_1. PMID: 4003975.
224	4008453	Antidepressants	1985	DeCastro RM. Antidepressants and myoclonus: case report. J Clin Psychiatry. 1985 Jul;46(7):284-7. PMID: 4008453.
225	4022263	DDT	1985	Pratt JA, Rothwell J, Jenner P, Marsden CD. Myoclonus in the rat induced by p,p'-DDT and the role of altered monoamine function. Neuropharmacology. 1985 May;24(5):361-73. doi: 10.1016/0028-3908(85)90020-6. PMID: 4022263.
226	4025853	Etomidate	1985	Giese JL, Stockham RJ, Stanley TH, Pace NL, Nelissen RH. Etomidate versus thiopental for induction of anesthesia. Anesth Analg. 1985 Sep;64(9):871-6. PMID: 4025853.
227	4025859	Etomidate	1985	Tasch MD. Myoclonus on recovery from etomidate. Anesth Analg. 1985 Sep;64(9):943. doi: 10.1213/00000539-198509000-00019. PMID: 4025859.
228	4037390	Etomidate	1985	Lumley J, Morgan M. Myoclonus after etomidate anesthesia. Anesth Analg. 1985 Oct;64(10):1034. doi: 10.1213/00000539-198510000-00024. PMID: 4037390.
229	4037466	Meperidine	1985	Goetting MG, Thirman MJ. Neurotoxicity of meperidine. Ann Emerg Med. 1985 Oct;14(10):1007-9. doi: 10.1016/s0196-0644(85)80251-1. PMID: 4037466.
230	4041819	Bicuculline	1985	Drugan RC, McIntyre TD, Alpern HP, Maier SF. Coping and seizure susceptibility: control over shock protects against bicuculline-induced seizures. Brain Res. 1985 Sep 2;342(1):9-17. doi: 10.1016/0006-8993(85)91347-2. PMID: 4041819.
231	4041911	Methylmercury	1985	O'Kusky JR, McGeer EG. Methylmercury poisoning of the developing nervous system in the rat: decreased activity of glutamic acid decarboxylase in cerebral cortex and neostriatum. Brain Res. 1985 Aug;353(2):299-306. doi: 10.1016/0165-3806(85)90219-6. PMID: 4041911.
232	4048610	Methyl bromide	1985	Audry D, Soichot P, Giard MH, Mauguière F. Une cause rare de myoclonies avec pes 'géants': l'intoxication au bromure de méthyle. A propos d'une observation à prédominance unilatérale [Rare cause of myoclonus with giant SEP's: methyl bromide poisoning. Apropos of a case with unilateral predominance]. Rev Electroencephalogr Neurophysiol Clin. 1985 Jul;15(1):45-52. French. doi: 10.1016/s0370-4475(85)80034-4. PMID: 4048610.

233	4063940	Phenelzine, tryptophan	1985	Levy AB, Bucher P, Votolato N. Myoclonus, hyperreflexia and diaphoresis in patients on phenelzine-tryptophan combination treatment. Can J Psychiatry. 1985 Oct;30(6):434-6. doi: 10.1177/070674378503000612. PMID: 4063940.
234	4067618	Chlorambucil	1985	LaDelfa I, Bayer N, Myers R, Hoffstein V. Chlorambucil-induced myoclonic seizures in an adult. J Clin Oncol. 1985 Dec;3(12):1691-2. doi: 10.1200/JCO.1985.3.12.1691. PMID: 4067618.
235	4089790	Buflomedil	1985	Leys D, Pavy G, Bourgeois P, Petit H. Myoclonies au cours d'un traitement par buflomedil [Myoclonus during treatment with buflomedil]. Therapie. 1985 Nov-Dec;40(6):481. French. PMID: 4089790.
236	4147723	5-hydroxytryptophan	1973	Klawans HL Jr, Goetz C, Weiner WJ. 5-Hydroxytryptophan-induced myoclonus in guinea pigs and the possible role of serotonin in infantile myoclonus. Neurology. 1973 Nov;23(11):1234-40. doi: 10.1212/wnl.23.11.1234. PMID: 4147723.
237	4209594	Piperazine	1974	Kömpf D, Neundörfer B. Neurotoxische Nebenwirkungen des Piperazins im Erwaxhsenenalter. Epileptischer Dämmerzustand mit Myoklonien [Neurotoxic side effects of piperazine in adults. Epileptic twilight state with myoclonia (author's transl)]. Arch Psychiatr Nervenkr (1970). 1974 Mar 4;218(3):223-33. German. doi: 10.1007/BF02401381. PMID: 4209594.
238	4218313	Bismuth	1974	Buge A, Rancurel G, Poisson M, Dechy H. Encøephalopathies myocloniques par les sels de bismuth. Six cas observés lors de traitements oraux au long cours [Myoclonic encephalopathies induced by bismuth salts. 6 cases observed during long-term oral treatment]. Nouv Presse Med. 1974 Oct 26;3(36):2315-20. French. PMID: 4218313.
239	4238586	Carbamazepine	1968	Wendland KL. Myoklonien nach Gaben von Carbamazepin [Myoclonus following doses of carbamazepin]. Nervenarzt. 1968 May;39(5):231-3. German. PMID: 4238586.
240	4354678	Contrast agent (angiografin)	1973	Löser R, Vogelsang H. Angiografin auch bei der lumbalen Myelographie anwendbar [Can angiografin be used for lumbar myelography?]. Fortschr Geb Rontgenstr Nuklearmed. 1973 Jun;118(6):654-7. German. PMID: 4354678.
241	4459861	Bismuth	1974	Cambier J, Mason M, Dairou R. Lettre: Encéphalopathie myoclonique et intoxication par les sels de bismuth [Letter: Myoclonic encephalopathy and bismuth salt poisoning]. Nouv Presse Med. 1974 Dec 21;3(44):2662. French. PMID: 4459861.
242	4469951	Bismuth	1974	Buge A, Rancurel G, Poisson M, Gazengel J, Dechy H, Fressinaud L, Emile J. 20 observations d'encéphalopathies aiguës avec myoclonies au cours de traitements oraux par les sels de bismuth [20 cases of acute encephalopathy with myoclonus during treatments with orally-administered bismuth salts]. Ann Med Interne (Paris). 1974 Dec;125(12):877-88. French. PMID: 4469951.
243	4479642	Tricyclic antidepressant	1974	Burks JS, Walker JE, Rumack BH, Ott JE. Tricyclic antidepressant poisoning. Reversal of coma, choreoathetosis, and myoclonus by physostigmine. JAMA. 1974 Dec 4;230(10):1405-7. doi: 10.1001/jama.230.10.1405. PMID: 4479642.
244	4536604	Bromomethane (methyl bromide)	1974	Mellerio F, Gaultier M, Bismut C. Electroencephalography during acute poisoning by methyl bromide. Eur J Toxicol Environ Hyg. 1974 Mar-Apr;7(2):119-32. English. PMID: 4536604.
245	4562133	Chloralose	1972	Lees P. Pharmacology and toxicology of alpha chloralose: a review. Vet Rec. 1972 Sep 30;91(14):330-3. doi: 10.1136/vr.91.14.330. PMID: 4562133.
246	4610013	Penicillin	1974	Fossieck B Jr, Parker RH. Neurotoxicity during intravenous infusion of penicillin. A review. J Clin Pharmacol. 1974 Oct;14(10):504-12. doi: 10.1002/j.1552-4604.1974.tb01364.x. PMID: 4610013.
247	4628883	Isoniazid	1972	Aach R, Kissane J. Generalized seizures following isoniazid therapy for tuberculosis in a patient with uremia. Am J Med. 1972 Dec;53(6):765-74. doi: 10.1016/0002-9343(72)90195-7. PMID: 4628883.
248	4649978	Penicillin	1972	Vital-Durand F, Gerin P, Pernier J, Revol M. Fuseaux de sommeil et pointes-ondes provoquées par la pénicilline chez le chat [Sleep spindles and wave spikes caused by penicillin in cats]. J Physiol (Paris). 1972 Oct;65:Suppl:313A-314. French. PMID: 4649978.
249	4657915	Lithium, dopa	1972	Setiey A, Moene Y, Chazot G, Trillet M, Courjon J. A propos d'un aspect clinique et électro-encéphalographique particulier provoqué par l'association lithium-dopa [Unusual clinical and electroencephalographic aspects induced by lithium-dopa association]. J Med Lyon. 1972 Oct 20;53(235):1401-3. French. PMID: 4657915.
250	4810682	Methaqualone	1974	Abboud RT, Freedman MT, Rogers RM, Daniele RP. Methaqualone poisoning with muscular hyperactivity necessitating the use of curare. Chest. 1974 Feb;65(2):204-5. doi: 10.1378/chest.65.2.204. PMID: 4810682.
251	4818163	Bismuth	1974	Burns R, Thomas DW, Barron VJ. Reversible encephalopathy possibly associated with bismuth subgallate ingestion. Br Med J. 1974 Feb 9;1(5901):220-3. doi: 10.1136/bmj.1.5901.220. PMID: 4818163; PMCID: PMC1633100.
252	4818793	Metrazol	1974	Jami L. Activity of alpha and gamma extensor motor axons during metrazol-induced seizures in cat. Exp Neurol. 1974 Apr;43(1):75-87. doi: 10.1016/0014-4886(74)90134-4. PMID: 4818793.
253	4990666	Chloralose	1970	Stoupel N, Monseu G, Cnockaert P. A propos de l'intoxication humaine par le chloralose. Etude clinique et électroencéphalographique [Human intoxication by chloralose. Clinical study and electroencephalography]. Acta Neurol Belg. 1970 May-Jun;70(3):352-8. French. PMID: 4990666.
254	4990955	Penicillin, carbenicillin	1970	Kurtzman NA, Rogers PW, Harter HR. Neurotoxic reaction to penicillin and carbenicillin. JAMA. 1970 Nov 16;214(7):1320-1. PMID: 4990955.
255	5008320	Penicillin	1972	Kao LI, Crill WE. Penicillin-induced segmental myoclonus. II. Membrane properties of cat spinal motoneurons. Arch Neurol. 1972 Feb;26(2):162-8. doi: 10.1001/archneur.1972.00490080080009. PMID: 5008320.
256	5015598	Mercury	1972	Snyder RD. The involuntary movements of chronic mercury poisoning. Arch Neurol. 1972 Apr;26(4):379-81. doi: 10.1001/archneur.1972.00490100109013. PMID: 5015598.

257	5049678	Urea	1972	Zuckermann EG, Glaser GH. Urea-induced myoclonic seizures. An experimental study of site of action and mechanism. Arch Neurol. 1972 Jul;27(1):14-28. doi: 10.1001/archneur.1972.00490130016003. PMID: 5049678.
258	5051253	Lithium	1972	Favarel-Garrigues B, Favarel-Garrigues JC, Bourgeois M. Deux cas d'intoxication grave par le carbonate de lithium [2 cases of severe poisoning by lithium carbonate]. Ann Med Psychol (Paris). 1972 Feb;1(2):253-7. French. PMID: 5051253.
259	5052305	Tricyclic antidepressant	1972	Schulze B. Zur Frage medikamentös induzierter cerebraler Reaktionen: Ein Fall von myoklonischen Status unter Behandlung mit tricyclishen Antidepressiva [Problem of drug-induced cerebral reactions: a case of myoclonic state under treatment with tricyclic antidepressive agents]. Nervenarzt. 1972 Jun;43(6):332-6. German. PMID: 5052305.
260	5090765	Lithium	1971	Wilson JH, Donker AJ, van der Hem GK, Wientjes J. Peritoneal dialysis for lithium poisoning. Br Med J. 1971 Jun 26;2(5764):749-50. doi: 10.1136/bmj.2.5764.749. PMID: 5090765; PMCID: PMC1796339.
261	5150097	Lithium	1971	Castaing R, Bony D, Favarel-Garrigues JC, Cardinaud JP. Intoxication aiguë par le carbonate de lithium [Acute intoxication by lithium carbonate]. Eur J Toxicol. 1971 Sep-Oct;4(5):412-5. French. PMID: 5150097.
262	5159060	Urea	1971	Zuckermann EC, Glaser GH. Experimental urea-induced myoclonic seizures: mechanism and site of action. Trans Am Neurol Assoc. 1971;96:101-5. PMID: 5159060.
263	5382528	Chloralose	1969	Moene Y, Cuche M, Trillet M, Motin J, Michel D. Problèmes diagnostiques posés par i'intoxication aiguë au chloralose (à propos de 6 cas) [Diagnostic problems posed by acute chloralose poisoning (6 cases)]. J Med Lyon. 1969 Nov 5;50(174):1483-93. French. PMID: 5382528.
264	5517898	Imipramine, amitriptyline	1970	Darcourt G, Fadeuilhe A, Lavagna J, Cazac A. Trois cas de myoclonies d'action au cours de traitements par l'imipramine et l'amitriptyline [Three cases of action myoclonus during treatment with imipramine and amitriptyline]. Rev Neurol (Paris). 1970 Feb;122(2):141-2. French. PMID: 5517898.
265	5518429	Chloralose	1970	Cornette M, Franck G. Aspects clinique et électroencéphalographique de l'intoxication médicamenteuse aiguë au chloralose. A propos de 11 observations récentes [Clinical and electroencephalographic aspects of acute drug poisoning with chloralose. Apropos of 11 recent cases]. Rev Neurol (Paris). 1970 Oct;123(4):268-72. French. PMID: 5518429.
266	6115101	Bicuculline	1981	Buckett WR. Intravenous bicuculline test in mice: characterization with GABAergic drugs. J Pharmacol Methods. 1981 Jan;5(1):35-41. doi: 10.1016/0160-5402(81)90100-5. PMID: 6115101.
267	6125000	Benzodiazepines	1982	Cepeda C, Valin A, Calderazzo L, Stutzmann JM, Naquet R. Myoclonies induites par certaines benzodiazepines chez le Papio papio. Comparaison avec les myoclonies induites par la stimulation lumineuse intermittente [Myoclonus induced by some benzodiazepines in the Papio papio. Comparison with myoclonus induced by intermittent light stimulation (author's transl)]. Rev Electroencephalogr Neurophysiol Clin. 1982 Apr;12(1):32-7. French. doi: 10.1016/s0370-4475(82)80006-3. PMID: 6125000.
268	6130489	Tryptamine	1982	Luscombe G, Jenner P, Marsden CD. Tryptamine-induced myoclonus in guinea-pigs pretreated with a monoamine oxidase inhibitor indicates pre- and post-synaptic actions of tryptamine upon central indoleamine systems. Neuropharmacology. 1982 Dec;21(12):1257-65. doi: 10.1016/0028-3908(82)90130-7. PMID: 6130489.
269	6146918	Neuroleptic	1984	Knezevic W, Mastaglia FL, Lefroy RB, Fisher A. Neuroleptic malignant syndrome. Med J Aust. 1984 Jan 7;140(1):28-30. doi: 10.5694/j.1326-5377.1984.tb103843.x. PMID: 6146918.
270	6156735	Chloralose	1980	Chadwick D, Hallett M, Jenner P, Marsden CD. Observations on chloralose-induced myoclonus in guinea-pigs. Br J Pharmacol. 1980 Jul;69(3):535-40. doi: 10.1111/j.1476-5381.1980.tb07045.x. PMID: 6156735; PMCID: PMC2044266.
271	6172264	Lorazepam	1981	Valin A, Cepeda C, Rey E, Naquet R. Opposite effects of lorazepam on two kinds of myoclonus in the photosensitive Papio papio. Electroencephalogr Clin Neurophysiol. 1981 Dec;52(6):647-51. doi: 10.1016/0013-4694(81)91439-5. PMID: 6172264.
272	6176920	DDT	1981	Chung Hwang E, Van Woert MH. p,p'-DDT-induced alterations in brain serotonin metabolism. Neurotoxicology. 1981 Dec;2(4):649-57. PMID: 6176920.
273	6187275	Meperidine	1983	Kaiko RF, Foley KM, Grabinski PY, Heidrich G, Rogers AG, Inturrisi CE, Reidenberg MM. Central nervous system excitatory effects of meperidine in cancer patients. Ann Neurol. 1983 Feb;13(2):180-5. doi: 10.1002/ana.410130213. PMID: 6187275.
274	6192824	Indoleamine	1983	Luscombe G, Jenner P, Marsden CD. Alterations in brain 5HT and tryptamine content during indoleamine-induced myoclonus in guinea pigs. Biochem Pharmacol. 1983 Jun 15;32(12):1857-64. doi: 10.1016/0006-2952(83)90050-3. PMID: 6192824.
275	6220294	Buflomedil	1983	Treves R, Desproges-Gotteron R. Encéphalopathie myoclonique chez une malade traitée par une dose excessive de buflomédil [Myoclonic encephalopathy in a patient treated with an excessive dose of buflomedil]. Presse Med. 1983 Mar 5;12(10):645. French. PMID: 6220294.
276	6222098	Isocarboxazid	1983	Davidson J, Turnbull C. Isocarboxazid. Efficacy and tolerance. J Affect Disord. 1983 May;5(2):183-9. doi: 10.1016/0165-0327(83)90012-5. PMID: 6222098.
277	6222351	Buflomedil	1983	Encéphalopathie myoclonique chez une malade traitée par une dose excessive de buflomédil [Myoclonic encephalopathy in a patient treated with an excessive dose of buflomedil]. Presse Med. 1983 May 28;12(23):1492. French. PMID: 6222351.
278	6274662	Pentylenetetrazol	1981	Lal H, Mann PA Jr, Shearman GT, Lippa AS. Effect of acute and chronic pentylenetetrazol treatment on benzodiazepine and cholinergic receptor binding in rat brain. Eur J Pharmacol. 1981 Oct 22;75(2-3):115-9. doi: 10.1016/0014-2999(81)90069-8. PMID: 6274662.
279	6297075	Dopamine	1982	Boudouresques G, Tafani B, Benichou M, Sarlon R. Encéphalopathie myoclonique à la dopamine [Myoclonal encephalopathy due to dopamine]. Sem Hop. 1982 Dec 16;58(46):2729-30. French. PMID: 6297075.

280	6322557	Etomidate	1983	Famewo CE. The safety of etomidate: a new intravenous anaesthetic induction agent. Afr J Med Med Sci. 1983 Jun;12(2):95-9. PMID: 6322557.
281	6378651	DDT	1984	Chung E, Van Woert MH. DDT myoclonus: sites and mechanism of action. Exp Neurol. 1984 Aug;85(2):273-82. doi: 10.1016/0014-4886(84)90140-7. PMID: 6378651.
282	6414652	Penicillin	1983	Greenwood RS, Godar SE, Winstead KK. Focal penicillin seizures--motor activity and cellular physiology and morphology. Brain Res Bull. 1983 Jul;11(1):91-101. doi: 10.1016/0361-9230(83)90057-6. PMID: 6414652.
283	6418061	Meperidine	1983	Hochman MS. Meperidine-associated myoclonus and seizures in long-term hemodialysis patients. Ann Neurol. 1983 Nov;14(5):593. doi: 10.1002/ana.410140520. PMID: 6418061.
284	6498517	Pilocarpine	1984	Turski WA, Cavalheiro EA, Bortolotto ZA, Mello LM, Schwarz M, Turski L. Seizures produced by pilocarpine in mice: a behavioral, electroencephalographic and morphological analysis. Brain Res. 1984 Nov 12;321(2):237-53. doi: 10.1016/0006-8993(84)90177-x. PMID: 6498517.
285	6521518	Metoclopramide	1984	Arias C, Roquer J, Herraiz J, Escudero D, Masó E. Mioclonías inducidas por metoclopramide [Myoclonus induced by metoclopramide]. Med Clin (Barc). 1984 Nov 10;83(15):648. Spanish. PMID: 6521518.
286	6522696	Beta-lactams	1984	Morán García V, Galbán Rodríguez C. Encefalopatía por beta-lactámicos [Encephalopathy caused by beta-lactamates]. Rev Clin Esp. 1984 Sep 15;174(5):183-6. Spanish. PMID: 6522696.
287	6527840	Benzodiazepine	1984	Rektor I, Bryere P, Valin A, Silva-Barrat C, Naquet R, Menini C. Physostigmine antagonizes benzodiazepine-induced myoclonus in the baboon, Papio papio. Neurosci Lett. 1984 Nov 23;52(1-2):91-6. doi: 10.1016/0304-3940(84)90356-2. PMID: 6527840.
288	6542228	Toluene	1984	Himnan DJ. Tolerance and reverse tolerance to toluene inhalation: effects on open-field behavior. Pharmacol Biochem Behav. 1984 Oct;21(4):625-31. doi: 10.1016/s0091-3057(84)80048-9. PMID: 6542228.
289	6639245	Metoclopramide	1983	Hyser CL, Drake ME Jr. Myoclonus induced by metoclopramide therapy. Arch Intern Med. 1983 Nov;143(11):2201-2. PMID: 6639245.
290	6656404	Penicillin	1983	Munar Qués M, Mulet Gutiérrez M, Cerdá Bibiloni JJ, Payeras Coli P, Vich Martorell CL. Encefalopatía penicilínica: estudio de dos casos [Penicillin encephalopathy: study of 2 cases]. Med Clin (Barc). 1983 Nov 5;81(14):632-3. Spanish. PMID: 6656404.
291	6696228	Etomidate	1984	Lowe SS. The prevention of etomidate-induced myoclonus. Anaesthesia. 1984 Jan;39(1):70-1. doi: 10.1111/j.1365-2044.1984.tb09471.x. PMID: 6696228.
292	6720247	Piperazine	1984	Neau JP, Rogez R, Boissonnot L, Simmat G, Gil R, Lefevre JP. Accidents neurologiques de la Pipérazine [Neurologic adverse effects of piperazine]. Acta Neurol Belg. 1984 Jan-Feb;84(1):26-34. French. PMID: 6720247.
293	6784438	Contrast agent (metrizamide)	1981	Bergvall U, Brismar T, Lying-Tunell U, Valdimarsson E. Confusion, myoclonus and speech arrest: epileptic manifestations after metrizamide myelography. Acta Neurol Scand. 1981 May;63(5):315-22. doi: 10.1111/j.1600-0404.1981.tb00785.x. PMID: 6784438.
294	6830193	Lithium	1983	Rosen PB, Stevens R. Action myoclonus in lithium toxicity. Ann Neurol. 1983 Feb;13(2):221-2. doi: 10.1002/ana.410130232. PMID: 6830193.
295	6838077	Meperidine	1983	Hershey LA. Meperidine and central neurotoxicity. Ann Intern Med. 1983 Apr;98(4):548-9. doi: 10.7326/0003-4819-98-4-548. PMID: 6838077.
296	6865851	Etomidate	1983	Etomidate for induction of anesthesia. Med Lett Drugs Ther. 1983 Jul 22;25(640):71-2. PMID: 6865851.
297	6869759	Etomidate	1983	Scorgie B. Etomidate infusion. Its use in anaesthesia for general surgery. Anaesthesia. 1983 Jul;38 Suppl:63-5. doi: 10.1111/j.1365-2044.1983.tb15182.x. PMID: 6869759.
298	6879270	Alcohol	1983	Drake ME Jr. Recurrent spontaneous myoclonus in alcohol withdrawal. South Med J. 1983 Aug;76(8):1040-2. doi: 10.1097/00007611-198308000-00028. PMID: 6879270.
299	6886043	Maprotiline	1983	Kettl P, DePaulo JR Jr. Maprotiline-induced myoclonus. J Clin Psychopharmacol. 1983 Aug;3(4):264-5. doi: 10.1097/00004714-198308000-00034. PMID: 6886043.
300	6890156	Penicillin	1982	Loeb C, Benassi E, Besio G, Maffini M, Tanganelli P. Liposome-entrapped GABA modifies behavioral and electrographic changes of penicillin-induced epileptic activity. Neurology. 1982 Nov;32(11):1234-8. doi: 10.1212/wnl.32.11.1237. PMID: 6890156.
301	6947139	Methaqualone	1981	Mack RB. Methaqualone intoxication. N C Med J. 1981 Nov;42(11):796. PMID: 6947139.
302	6973711	5-hydroxytryptophan	1981	Thal LJ, Wolfson LI. Functional anatomy of L-5-hydroxytryptophan-induced myoclonus in the guinea pig. Neurology. 1981 Aug;31(8):955-60. doi: 10.1212/wnl.31.8.955. PMID: 6973711.
303	7027723	Etomidate	1981	Helmers JH, Adam AA, Giezen J. Pain and myoclonus during induction with etomidate. A double-blind, controlled evaluation of the influence of droperidol and fentanyl. Acta Anaesthesiol Belg. 1981;32(2):141-7. PMID: 7027723.

304	7056707	Lead	1982	Goldings AS, Stewart RM. Organic lead encephalopathy: behavioral change and movement disorder following gasoline inhalation. J Clin Psychiatry. 1982 Feb;43(2):70-2. PMID: 7056707.
305	7059133	L-tryptophan	1982	Baloh RW, Dietz J, Spooner JW. Myoclonus and ocular oscillations induced by L-tryptophan. Ann Neurol. 1982 Jan;11(1):95-7. doi: 10.1002/ana.410110117. PMID: 7059133.
306	7078834	Picrotoxin	1982	Slater P, Dickinson SL. Role of acetylcholine and dopamine in myoclonus induced by intrastriatal picrotoxin. Neurosci Lett. 1982 Mar 5;28(3):253-7. doi: 10.1016/0304-3940(82)90066-0. PMID: 7078834.
307	7087678	Indole	1982	Luscombe G, Jenner P, Marsden CD. Myoclonus in guniea pigs is induced by indole-containing but not piperazine-containing 5HT agonists. Life Sci. 1982 Apr 26;30(17):1487-94. doi: 10.1016/0024-3205(82)90563-x. PMID: 7087678.
308	7109770	Bismuth	1982	Cervelló MA, Alfaro A, Antolín MA, Peñarrocha M. Encefalopatía mioclónica bismútica: a propósito de dos nuevas observaciones clínicas [Bismuth myoclonic encephalopathy. Report of two cases (author's transl)]. Med Clin (Barc). 1982 Jul 1-31;79(3):137-40. Spanish. PMID: 7109770.
309	7110997	Triprolidine, pseudoephedrine, paracetamol	1982	Jacquesson M, Saudeau D, Pantin B, Girard JJ, Groussin P. Myoclonies imputables à une association de triprolidine, de pseudo-éphédrine et de paracétamol [Myoclonia caused by a combination of triprolidine, pseudoephedrine and paracetamol]. Nouv Presse Med. 1982 Jun 26;11(30):2298-9. French. PMID: 7110997.
310	7139632	Levodopa	1982	Nausieda PA, Weiner WJ, Kaplan LR, Weber S, Klawans HL. Sleep disruption in the course of chronic levodopa therapy: an early feature of the levodopa psychosis. Clin Neuropharmacol. 1982;5(2):183-94. doi: 10.1097/00002826-198205020-00003. PMID: 7139632.
311	7156442	Strychnine, chloralose, isoniazid, tricyclic antidepressant agent, lithium, bromomethane (methyl bromide)	1982	Mellerio F, Levy-Alcover MA. Myoclonies d'origine toxique [Myoclonus of toxic origin]. Rev Electroencephalogr Neurophysiol Clin. 1982 Nov;12(3):210-8. French. doi: 10.1016/s0370-4475(82)80046-4. PMID: 7156442.
312	7168056	Piperazine	1982	Lynggaard F, Jensen HA. Svaer piperazinforgiftning behandlet med haemodialyse hos patient med nedsat nyrefunktion [Severe piperazine poisoning treated with hemodialysis in a patient with impaired kidney function]. Ugeskr Laeger. 1982 Nov 29;144(48):3582-3. Danish. PMID: 7168056.
313	7170003	Rattlesnake venom	1982	Brick JF, Gutmann L. Rattlesnake venom-induced myokymia. Muscle Nerve. 1982;5(9S):S98-100. PMID: 7170003.
314	7194979	Carbamazepine	1981	Sullivan JB Jr, Rumack BH, Peterson RG. Acute carbamazepine toxicity resulting from overdose. Neurology. 1981 May;31(5):621-4. doi: 10.1212/wnl.31.5.621. PMID: 7194979.
315	7200212	Levodopa	1982	Glantz R, Weiner WJ, Goetz CG, Nausieda PA, Klawans HL. Drug-induced asterixis in Parkinson disease. Neurology. 1982 May;32(5):553-5. doi: 10.1212/wnl.32.5.553. PMID: 7200212.
316	7247781	Contrast agent (metrizamide)	1981	Gimenez M, Martinez F, Feijoo M. Asterixis of myoclonus after metrizamide myelography. Arch Neurol. 1981 Jul;38(7):472. doi: 10.1001/archneur.1981.00510070106034. PMID: 7247781.
317	7266934	DDT	1981	Hwang EC, Plaitakis A, Magnussen I, Van Woert MH. Relationship of inferior olive-climbing fibers to p,p'-DDT-induced myoclonus in rats. Neurosci Lett. 1981 Jun 12;24(1):103-8. doi: 10.1016/0304-3940(81)90367-0. PMID: 7266934.
318	7304118	Etomidate	1981	Gepts E, Camu F. Total intravenous anesthesia with etomidate for microlaryngoscopy. Applicability and shortcomings. Acta Anaesthesiol Belg. 1981;32(3):177-84. PMID: 7304118.
319	7329800	Bismuth	1981	Goas JY, Borsotti JP, Missoum A, Allain P, Chaleil D. Encéphalopathie myoclinique par le sous-nitrate de bismuth. Une observation récente [Myoclonic encephalopathy due to bismuth subnitrate. A recent case]. Nouv Presse Med. 1981 Dec 26;10(47):3855. French. PMID: 7329800.
320	7352541	MAO-inhibitor	1980	Cohen RM, Pickar D, Murphy DL. Myoclonus-associated hypomania during MAO-inhibitor treatment. Am J Psychiatry. 1980 Jan;137(1):105-6. doi: 10.1176/ajp.137.1.105. PMID: 7352541.
321	7383279	L-Tryptophan	1980	Brown DR, Growdon JH. L-Tryptophan administration potentiates serotonin-dependent myoclonic behavior in the rat. Neuropharmacology. 1980 Apr;19(4):343-7. doi: 10.1016/0028-3908(80)90185-9. PMID: 7383279.
322	7400961	Pentylenetetrazol	1980	Yonekawa WD, Kupferberg HJ, Woodbury DM. Relationship between pentylenetetrazol-induced seizures and brain pentylenetetrazol levels in mice. J Pharmacol Exp Ther. 1980 Sep;214(3):589-93. PMID: 7400961.
323	7410114	Chlorambucil	1980	Ammenti A, Reitter B, Müller-Wiefel DE. Chlorambucil neurotoxicity: report of two cases. Helv Paediatr Acta. 1980 Jul;35(3):281-7. PMID: 7410114.
324	7417051	Penicillin	1980	Lothman EW. Intravenous penicillin and myoclonus. Arch Neurol. 1980 Aug;37(8):531. doi: 10.1001/archneur.1980.00500570079021. PMID: 7417051.
325	7423414	Bismuth	1980	Gastaut JL, Jouglard J. Confrontation clinique et électro=encéphalographique au cours de l'encéphalopathie myoclonique bismuthique [Clinical and electroencephalographic correlates in bismuth-induced myoclonic encephalopathy]. Therapie. 1980 May-Jun;35(3):293-6. French. PMID: 7423414.

326	7448613	Enflurane	1980	Ng AT. Prolonged myoclonic contractions after enflurane anaesthesia - a case report. Can Anaesth Soc J. 1980 Sep;27(5):502-3. doi: 10.1007/BF03007053. PMID: 7448613.
327	7476110	Contrast agent	1991	Micheli F, Gatto E, Lehkuniec E. Mioclonus espinal secundario a la administración endovenosa de contraste iodado [Spinal myoclonus secondary to the intravenous administration of iodine contrast media]. Medicina (B Aires). 1991;51(6):548-50. Spanish. PMID: 7476110.
328	7485224	Thyrotropin-releasing hormone	1995	Ishii K, Hayashi A, Tamaoka A, Usuki S, Mizusawa H, Shoji S. Case report: thyrotropin-releasing hormone-induced myoclonus and tremor in a patient with Hashimoto's encephalopathy. Am J Med Sci. 1995 Nov;310(5):202-5. doi: 10.1097/00000441-199511000-00005. PMID: 7485224.
329	7485652	Fluoxetine, buspirone	1995	Lauterbach EC. Fluoxetine, buspirone, myoclonus, and dystonia. Am J Psychiatry. 1995 Nov;152(11):1697. doi: 10.1176/ajp.152.11.1697a. PMID: 7485652.
330	7486124	Opioid	1995	Mercadante S. Dantrolene treatment of opioid-induced myoclonus. Anesth Analg. 1995 Dec;81(6):1307-8. doi: 10.1097/00000539-199512000-00034. PMID: 7486124.
331	7501141	Cyclosporine	1995	Wijdicks EF, Wiesner RH, Krom RA. Neurotoxicity in liver transplant recipients with cyclosporine immunosuppression. Neurology. 1995 Nov;45(11):1962-4. doi: 10.1212/wnl.45.11.1962. PMID: 7501141.
332	7513374	Opioids	1994	Hammack JE, Loprinzi CL. Use of orally administered opioids for cancer-related pain. Mayo Clin Proc. 1994 Apr;69(4):384-90. doi: 10.1016/s0025-6196(12)62226-5. PMID: 7513374.
333	7522149	Clozapine	1994	Malow BA, Reese KB, Sato S, Bogard PJ, Malhotra AK, Su TP, Pickar D. Spectrum of EEG abnormalities during clozapine treatment. Electroencephalogr Clin Neurophysiol. 1994 Sep;91(3):205-11. doi: 10.1016/0013-4694(94)90070-1. PMID: 7522149.
334	7525780	Heroin (diamorphine)	1993	Cartwright PD, Hesse C, Jackson AO. Myoclonic spasms following intrathecal diamorphine. J Pain Symptom Manage. 1993 Oct;8(7):492-5. doi: 10.1016/0885-3924(93)90192-x. PMID: 7525780.
335	7526455	Flecainide	1994	Ghika J, Goy JJ, Naegeli C, Regli F. Acute reversible ataxo-myoclonic encephalopathy with flecainide therapy. Schweiz Arch Neurol Psychiatr (1985). 1994;145(1):4-6. PMID: 7526455.
336	7532570	Valproate	1995	Aguglia U, Gambardella A, Zappia M, Valentino P, Quattrone A. Negative myoclonus during valproate-related stupor. Neurophysiological evidence of a cortical non-epileptic origin. Electroencephalogr Clin Neurophysiol. 1995 Feb;94(2):103-8. doi: 10.1016/0013-4694(94)00268-p. PMID: 7532570.
337	7543228	Morphine	1995	Jacobsen LS, Olsen AK, Sjøgren P, Jensen NH. Morfininduceret hyperalgesi, allodyni og myoklonus--nye morfinbivirkninger? [Morphine-induced hyperalgesia, allodynia and myoclonus--new side-effects of morphine?]. Ugeskr Laeger. 1995 Jun 5;157(23):3307-10. Danish. PMID: 7543228.
338	7574072	Chloralose	1995	Quinio P, Bouche O, Rossignol B, de Tinteniac A. Propofol in the management of myoclonus syndrome induced by chloralose poisoning. Anesthesiology. 1995 Oct;83(4):875. doi: 10.1097/00000542-199510000-00032. PMID: 7574072.
339	7586117	Propofol	1995	Hughes NJ, Lyons JB. Prolonged myoclonus and meningism following propofol. Can J Anaesth. 1995 Aug;42(8):744-6. doi: 10.1007/BF03012676. PMID: 7586117.
340	7644248	Morphine	1995	Tiseo PJ, Thaler HT, Lapin J, Inturrisi CE, Portenoy RK, Foley KM. Morphine-6-glucuronide concentrations and opioid-related side effects: a survey in cancer patients. Pain. 1995 Apr;61(1):47-54. doi: 10.1016/0304-3959(94)00148-8. PMID: 7644248.
341	7665541	Clozapine	1995	Bak TH, Bauer M, Schaub RT, Hellweg R, Reischies FM. Myoclonus in patients treated with clozapine: a case series. J Clin Psychiatry. 1995 Sep;56(9):418-22. PMID: 7665541.
342	7673770	Opioid	1995	de Stoutz ND, Bruera E, Suarez-Almazor M. Opioid rotation for toxicity reduction in terminal cancer patients. J Pain Symptom Manage. 1995 Jul;10(5):378-84. doi: 10.1016/0885-3924(95)90924-c. PMID: 7673770.
343	7711342	Morphine	1995	Holdsworth MT, Adams VR, Chavez CM, Vaughan LJ, Duncan MH. Continuous midazolam infusion for the management of morphine-induced myoclonus. Ann Pharmacother. 1995 Jan;29(1):25-9. doi: 10.1177/106002809502900105. PMID: 7711342.
344	7727612	Lorazepam	1994	Lee DS, Wong HA, Knoppert DC. Myoclonus associated with lorazepam therapy in very-low-birth-weight infants. Biol Neonate. 1994;66(6):311-5. doi: 10.1159/000244123. PMID: 7727612.
345	7732767	Vigabatrin	1995	Marciani MG, Maschio M, Spanedda F, Iani C, Gigli GL, Bernardi G. Development of myoclonus in patients with partial epilepsy during treatment with vigabatrin: an electroencephalographic study. Acta Neurol Scand. 1995 Jan;91(1):1-5. doi: 10.1111/j.1600-0404.1995.tb05834.x. PMID: 7732767.
346	7737496	Ciprofloxacin	1995	Farrington J, Stoudemire A, Tierney J. The role of ciprofloxacin in a patient with delirium due to multiple etiologies. Gen Hosp Psychiatry. 1995 Jan;17(1):47-53. doi: 10.1016/0163-8343(94)00065-l. PMID: 7737496.
347	7753066	Bismuth	1995	Gordon MF, Abrams RI, Rubin DB, Barr WB, Correa DD. Bismuth subsalicylate toxicity as a cause of prolonged encephalopathy with myoclonus. Mov Disord. 1995 Mar;10(2):220-2. doi: 10.1002/mds.870100215. PMID: 7753066.
348	7761544	Psychotropic	1995	Brogmus KE, Lesch A. Psychopharmakainduzierte Myoklonien [Psychotropic drug-induced myoclonus]. Psychiatr Prax. 1995 Mar;22(2):77-9. German. PMID: 7761544.
349	7776924	Moclobemide, pethidine	1995	Gillman PK. Possible serotonin syndrome with moclobemide and pethidine. Med J Aust. 1995 May 15;162(10):554. doi: 10.5694/j.1326-5377.1995.tb138527.x. PMID: 7776924.

350	7786955	Clozapine	1995	Denney D, Stevens JR. Clozapine and seizures. Biol Psychiatry. 1995 Apr 1;37(7):427-33. doi: 10.1016/0006-3223(94)00237-W. PMID: 7786955.
351	7806697	Fluoxetine	1994	D'Souza DC, Bennett A, Abi-Dargham A, Krystal JH. Precipitation of a psychoneuromotor syndrome by fluoxetine in a haloperidol-treated schizophrenic patient. J Clin Psychopharmacol. 1994 Oct;14(5):361-3. PMID: 7806697.
352	7818134	Postanesthesia	1995	Barbaccia JJ. Myoclonus in the postanesthesia care unit after an intraoperative myelogram. Anesth Analg. 1995 Feb;80(2):413-4. doi: 10.1097/00000539-199502000-00036. PMID: 7818134.
353	7892029	Morphine	1994	Sjøgren P, Jensen NH, Jensen TS. Disappearance of morphine-induced hyperalgesia after discontinuing or substituting morphine with other opioid agonists. Pain. 1994 Nov;59(2):313-316. doi: 10.1016/0304-3959(94)90084-1. PMID: 7892029.
354	7907921	Lithium	1994	Prettyman R. Lithium neurotoxicity at subtherapeutic serum levels. Br J Psychiatry. 1994 Jan;164(1):123. doi: 10.1192/bjp.164.1.123a. PMID: 7907921.
355	7949522	Acyclovir	1994	Mataix AL, Duarte J, Revuelta K, Fuentes MJ, Ruiz MD, Gavira R, López MC. Oral acyclovir and neurologic adverse effects in endstage renal disease. Ann Pharmacother. 1994 Jul-Aug;28(7-8):961-2. doi: 10.1177/106002809402800724. PMID: 7949522.
356	7951645	Imipramine	1994	Black KJ, Kilzieh N. Severe imipramine-induced myoclonus in a patient with psychotic bipolar depression, catatonia, and schizencephaly. Ann Clin Psychiatry. 1994 Mar;6(1):45-9. doi: 10.3109/10401239409148839. PMID: 7951645.
357	7963623	Cyclosporin	1994	Ellis ME, Spence D, Ernst P, Meunier F. Is cyclosporin neurotoxicity enhanced in the presence of liposomal amphotericin B? J Infect. 1994 Jul;29(1):106-7. doi: 10.1016/s0163-4453(94)95303-1. PMID: 7963623.
358	8007042	Meperidine	1994	Adair JC, Gilmore RL. Meperidine neurotoxicity after organ transplantation. J Toxicol Clin Toxicol. 1994;32(3):325-8. doi: 10.3109/15563659409017968. PMID: 8007042.
359	8010436	Sertraline	1994	Jahr JS, Pisto JD, Gitlin MC, O'Neill PT. The serotonin syndrome in a patient receiving sertraline after an ankle block. Anesth Analg. 1994 Jul;79(1):189-91. doi: 10.1213/00000539-199407000-00037. PMID: 8010436.
360	8033934	Midazolam	1994	Magny JF, d'Allest AM, Nedelcoux H, Zupan V, Dehan M. Midazolam and myoclonus in neonate. Eur J Pediatr. 1994 May;153(5):389-90. doi: 10.1007/BF01956430. PMID: 8033934.
361	8041377	Fluoxetine	1994	Lauterbach EC. Reversible intermittent rhythmic myoclonus with fluoxetine in presumed Pick's disease. Mov Disord. 1994 May;9(3):343-6. doi: 10.1002/mds.870090314. PMID: 8041377.
362	8054587	Picrotoxin	1993	Iakimovskiĭ AF. Mioklonicheskiĭ giperkinez, vyzyvaemyĭ povtornym vvedeniem v neostriatum krys pikrotoksina [Myoclonic hyperkinesis induced by repeated administration of picrotoxin into rat neostriatum]. Biull Eksp Biol Med. 1993 Jan;115(1):7-9. Russian. PMID: 8054587.
363	8059167	Piperazine	1994	Drouet A, Valance J. Myoclonies de repos et d'action induites par la pipérazine [Myoclonus in rest and exertion induced by piperazine]. Rev Med Interne. 1994 May;15(5):364-5. French. doi: 10.1016/s0248-8663(05)81448-1. PMID: 8059167.
364	8080553	Clozapine	1994	Meltzer HY, Ranjan R. Valproic acid treatment of clozapine-induced myoclonus. Am J Psychiatry. 1994 Aug;151(8):1246-7. doi: 10.1176/ajp.151.8.1246b. PMID: 8080553.
365	8122952	Ceftazidime	1994	Klion AD, Kallsen J, Cowl CT, Nauseef WM. Ceftazidime-related nonconvulsive status epilepticus. Arch Intern Med. 1994 Mar 14;154(5):586-9. PMID: 8122952.
366	8135401	Opioids	1994	Bowdle TA, Rooke GA. Postoperative myoclonus and rigidity after anesthesia with opioids. Anesth Analg. 1994 Apr;78(4):783-6. doi: 10.1213/00000539-199404000-00030. PMID: 8135401.
367	8139601	Levodopa	1994	Marconi R, Lefebvre-Caparros D, Bonnet AM, Vidailhet M, Dubois B, Agid Y. Levodopa-induced dyskinesias in Parkinson's disease phenomenology and pathophysiology. Mov Disord. 1994 Jan;9(1):2-12. doi: 10.1002/mds.870090103. PMID: 8139601.
368	8141521	Pefloxacin	1993	Durand JM, Telle H, Quilès N, Taramasco V, Kaplanski G, Soubeyrand J. Syndrome confusionnel, myoclonies et traitement par la péfloxacine [Confusion syndrome, myoclonus and treatment with pefloxacin]. Ann Med Interne (Paris). 1993;144(7):495-6. French. PMID: 8141521.
369	8142134	Prednimustine	1994	Martin M, Diaz-Rubio E, Casado A, Valverde JJ, Garcia Urra D, López-Martin JA, Rodriguez-Lescure A. Prednimustine-induced myoclonus--a report of three cases. Acta Oncol. 1994;33(1):81-2. doi: 10.3109/02841869409098387. PMID: 8142134.
370	8208718	Tricyclic antidepressive	1994	Lejoyeux M, Ades J, Rouillon F. Myoclonies chez les patients traités par antidépresseurs tricycliques. Evaluation prospective [Myoclonia in patients treated with tricyclic antidepressive agents. Prospective evaluation]. Presse Med. 1994 Mar 5;23(9):447. French. PMID: 8208718.
371	8214650	Etomidate	1993	Hullander RM, Leivers D, Wingler K. A comparison of propofol and etomidate for cardioversion. Anesth Analg. 1993 Oct;77(4):690-4. doi: 10.1213/00000539-199310000-00006. PMID: 8214650.
372	8214699	Etomidate	1993	Reddy RV, Moorthy SS, Dierdorf SF, Deitch RD Jr, Link L. Excitatory effects and electroencephalographic correlation of etomidate, thiopental, methohexital, and propofol. Anesth Analg. 1993 Nov;77(5):1008-11. doi: 10.1213/00000539-199311000-00023. PMID: 8214699.
373	8215972	Ifosfamide	1993	Wengs WJ, Talwar D, Bernard J. Ifosfamide-induced nonconvulsive status epilepticus. Arch Neurol. 1993 Oct;50(10):1104-5. doi: 10.1001/archneur.1993.00540100089024. PMID: 8215972.

374	8278214	Morphine	1993	Sjøgren P, Jonsson T, Jensen NH, Drenck NE, Jensen TS. Hyperalgesia and myoclonus in terminal cancer patients treated with continuous intravenous morphine. Pain. 1993 Oct;55(1):93-97. doi: 10.1016/0304-3959(93)90188-U. PMID: 8278214.
375	8298383	Propafenone	1994	Chua TP, Farrell T, Lipkin DP. Myoclonus associated with propafenone. BMJ. 1994 Jan 8;308(6921):113. doi: 10.1136/bmj.308.6921.113b. PMID: 8298383; PMCID: PMC2539204.
376	8301877	Levodopa	1993	Yoshida K, Moriwaka F, Matsuura T, Hamada T, Tashiro K. Myoclonus and seizures in a patient with parkinsonism: induction by levodopa and its confirmation on SEPs. Jpn J Psychiatry Neurol. 1993 Sep;47(3):621-5. doi: 10.1111/j.1440-1819.1993.tb01808.x. PMID: 8301877.
377	8309648	Fluoxetine	1993	Messiha FS. Fluoxetine: a spectrum of clinical applications and postulates of underlying mechanisms. Neurosci Biobehav Rev. 1993 Winter;17(4):385-96. doi: 10.1016/s0149-7634(05)80115-0. PMID: 8309648.
378	8317732	Morphine	1993	de Armendi AJ, Fahey M, Ryan JF. Morphine-induced myoclonic movements in a pediatric pain patient. Anesth Analg. 1993 Jul;77(1):191-2. doi: 10.1213/00000539-199307000-00038. PMID: 8317732.
379	8407142	Catechol, urea	1993	Touge T, Takeuchi H, Yamada A, Miki H, Nishioka M. Electrophysiological comparison between catechol- and urea-induced myoclonus models in the rat. Int J Neurosci. 1993 Jul-Aug;71(1-4):159-71. doi: 10.3109/00207459309000601. PMID: 8407142.
380	8503423	Opiates	1993	Mattana J, Ahn J, Desroches L, Fitzmaurice S, Singhal PC. Naloxone-responsive encephalopathy in end-stage renal disease. Am J Kidney Dis. 1993 Jun;21(6):669-72. doi: 10.1016/s0272-6386(12)80042-4. PMID: 8503423.
381	8530337	Fluvoxamine	1995	Bauer M. Severe myoclonus produced by fluvoxamine. J Clin Psychiatry. 1995 Dec;56(12):589-90. PMID: 8530337.
382	8552126	Fentanyl	1995	Petzinger G, Mayer SA, Przedborski S. Fentanyl-induced dyskinesias. Mov Disord. 1995 Sep;10(5):679-80. doi: 10.1002/mds.870100526. PMID: 8552126.
383	8559401	Methyl ethyl ketone	1996	Ortí-Pareja M, Jiménez-Jiménez FJ, Miquel J, Montero E, Cabrera-Valdivia F, Benito A, García-Albea E. Reversible myoclonus, tremor, and ataxia in a patient exposed to methyl ethyl ketone. Neurology. 1996 Jan;46(1):272. doi: 10.1212/wnl.46.1.272. PMID: 8559401.
384	8577386	Gallamine	1995	Burke W, Sears TA, Seers C. Spontaneous synchronized neural activity in decerebrate gallamine-paralysed cats. Neuroscience. 1995 Oct;68(3):943-53. doi: 10.1016/0306-4522(95)00166-g. PMID: 8577386.
385	8594125	Propofol	1995	Mercadante S, De Conno F, Ripamonti C. Propofol in terminal care. J Pain Symptom Manage. 1995 Nov;10(8):639-42. doi: 10.1016/0885-3924(95)00131-x. PMID: 8594125.
386	8630910	Opioid	1995	Cherny NJ, Chang V, Frager G, Ingham JM, Tiseo PJ, Popp B, Portenoy RK, Foley KM. Opioid pharmacotherapy in the management of cancer pain: a survey of strategies used by pain physicians for the selection of analgesic drugs and routes of administration. Cancer. 1995 Oct 1;76(7):1283-93. doi: 10.1002/1097-0142(19951001)76:7<1283::aid-cncr2820760728>3.0.co;2-0. PMID: 8630910.
387	8635188	Vigabatrin	1995	Neufeld MY, Vishnevska S. Vigabatrin and multifocal myoclonus in adults with partial seizures. Clin Neuropharmacol. 1995 Jun;18(3):280-3. doi: 10.1097/00002826-199506000-00010. PMID: 8635188.
388	8644960	Flumazenil	1996	Gueye PN, Hoffman JR, Taboulet P, Vicaut E, Baud FJ. Empiric use of flumazenil in comatose patients: limited applicability of criteria to define low risk. Ann Emerg Med. 1996 Jun;27(6):730-5. doi: 10.1016/s0196-0644(96)70191-9. PMID: 8644960.
389	8644975	Fentanyl	1996	Adair JC, el-Nachef A, Cutler P. Fentanyl neurotoxicity. Ann Emerg Med. 1996 Jun;27(6):791-2. doi: 10.1016/s0196-0644(96)70205-6. PMID: 8644975.
390	8684512	NA	1996	Poersch M, Hufnagel A, Smolenski C. Medikamentös induzierte Asterixis verstärkt durch relative Hypoglykämie [Drug-induced asterixis amplified by relative hypoglycemia]. Nervenarzt. 1996 Apr;67(4):323-6. German. PMID: 8684512.
391	8704069	Clozapine	1996	Sajatovic M, Meltzer HY. Clozapine-induced myoclonus and generalized seizures. Biol Psychiatry. 1996 Mar 1;39(5):367-70. doi: 10.1016/0006-3223(95)00499-8. PMID: 8704069.
392	8723864	Propofol	1996	Strachan AN, Raithatha HH. Propofol myoclonus. Can J Anaesth. 1996 May;43(5 Pt 1):536-7. doi: 10.1007/BF03018121. PMID: 8723864.
393	8734484	Pilocarpine	1996	Alam AM, Starr MS. Regional changes in brain dopamine utilization during status epilepticus in the rat induced by systemic pilocarpine and intrahippocampal carbachol. Neuropharmacology. 1996 Feb;35(2):159-67. doi: 10.1016/0028-3908(95)00154-9. PMID: 8734484.
394	8739122	Flurothyl	1996	Baraban SC, Schwartzkroin PA. Flurothyl seizure susceptibility in rats following prenatal methylazoxymethanol treatment. Epilepsy Res. 1996 Apr;23(3):189-94. doi: 10.1016/0920-1211(95)00094-1. PMID: 8739122.
395	8771080	Amantadine	1996	Pfeiffer RF. Amantadine-induced "vocal" myoclonus. Mov Disord. 1996 Jan;11(1):104-6. doi: 10.1002/mds.870110123. PMID: 8771080.
396	8794509	Clozapine	1996	Vrtunski PB, Konicki PE, Kwon KY, Jurjus G, Jaskiw GE. Effect of clozapine on motor function in schizophrenic patients. Schizophr Res. 1996 May;20(1-2):187-98. doi: 10.1016/0920-9964(95)00085-2. Erratum in: Schizophr Res 1996 Dec 15;22(3):269. Jaskiw GJ [corrected to Jurjus G]. PMID: 8794509.
397	8822698	Gabapentin	1996	Reeves AL, So EL, Sharbrough FW, Krahn LE. Movement disorders associated with the use of gabapentin. Epilepsia. 1996 Oct;37(10):988-90. doi: 10.1111/j.1528-1157.1996.tb00537.x. PMID: 8822698.
398	8835715	Clozapine	1996	Barak Y, Levine J, Weisz R. Clozapine-induced myoclonus: two case reports. J Clin Psychopharmacol. 1996 Aug;16(4):339-40. doi: 10.1097/00004714-199608000-00016. PMID: 8835715.
399	8866441	Piperacillin	1996	Park-Matsumoto YC, Tazawa T. Piperacillin-induced encephalopathy. J Neurol Sci. 1996 Sep 1;140(1-2):141-2. doi:

				10.1016/0022-510x(96)00119-0. PMID: 8866441.
400	8869767	NA	1996	Mathew NT, Tietjen GE, Lucker C. Serotonin syndrome complicating migraine pharmacotherapy. Cephalalgia. 1996 Aug;16(5):323-7. doi: 10.1046/j.1468-2982.1996.1605323.x. PMID: 8869767.
401	8876879	Opioid	1996	Pereira JL, Bruera ED. Comment: opioid-induced muscle activity. Ann Pharmacother. 1996 Sep;30(9):1042-3. doi: 10.1177/106002809603000931. PMID: 8876879.
402	8903083	Diatrizoate meglumine (contrast agent)	1996	Sam MC, Gutmann L. Spinal myoclonus following intrathecal administration of diatrizoate meglumine. J Neuroimaging. 1996 Oct;6(4):256-8. doi: 10.1111/jon199664256. PMID: 8903083.
403	8908234	Sevoflurane	1996	Terasako K, Ishii S. Postoperative seizure-like activity following sevoflurane anesthesia. Acta Anaesthesiol Scand. 1996 Sep;40(8 Pt 1):953-4. doi: 10.1111/j.1399-6576.1996.tb04566.x. PMID: 8908234.
404	8937794	Phenytoin	1996	Duarte J, Sempere AP, Cabezas MC, Marcos J, Clavería LE. Postural myoclonus induced by phenytoin. Clin Neuropharmacol. 1996 Dec;19(6):536-8. doi: 10.1097/00002826-199619060-00009. PMID: 8937794.
405	8938312	Gadopentetate dimeglumine	1996	Ray DE, Cavanagh JB, Nolan CC, Williams SC. Neurotoxic effects of gadopentetate dimeglumine: behavioral disturbance and morphology after intracerebroventricular injection in rats. AJNR Am J Neuroradiol. 1996 Feb;17(2):365-73. PMID: 8938312; PMCID: PMC8338359.
406	8998190	Prednimustine	1997	Monnerat C, Gander M, Leyvraz S. A rare case of prednimustine-induced myoclonus. J Natl Cancer Inst. 1997 Jan 15;89(2):173-4. doi: 10.1093/jnci/89.2.173. PMID: 8998190.
407	9031998	Trazodone, amitriptyline, lithium	1996	Nisijima K, Shimizu M, Abe T, Ishiguro T. A case of serotonin syndrome induced by concomitant treatment with low-dose trazodone and amitriptyline and lithium. Int Clin Psychopharmacol. 1996 Dec;11(4):289-90. doi: 10.1097/00004850-199612000-00013. PMID: 9031998.
408	9034417	Chlorambucil	1997	Wyllie AR, Bayliff CD, Kovacs MJ. Myoclonus due to chlorambucil in two adults with lymphoma. Ann Pharmacother. 1997 Feb;31(2):171-4. doi: 10.1177/106002809703100207. PMID: 9034417.
409	9060031	Fentanyl	1997	Bruera E, Pereira J. Acute neuropsychiatric findings in a patient receiving fentanyl for cancer pain. Pain. 1997 Jan;69(1-2):199-201. doi: 10.1016/s0304-3959(96)03238-1. PMID: 9060031.
410	9061094	Morphine	1997	Christrup LL. Morphine metabolites. Acta Anaesthesiol Scand. 1997 Jan;41(1 Pt 2):116-22. doi: 10.1111/j.1399-6576.1997.tb04625.x. PMID: 9061094.
411	9061098	Opioid	1997	Bruera E, Lawlor P. Cancer pain management. Acta Anaesthesiol Scand. 1997 Jan;41(1 Pt 2):146-53. doi: 10.1111/j.1399-6576.1997.tb04629.x. PMID: 9061098.
412	9077578	Morphine	1997	Kolesnikov Y, Jain S, Wilson R, Pasternak GW. Blockade of morphine-induced hindlimb myoclonic seizures in mice by ketamine. Pharmacol Biochem Behav. 1997 Mar;56(3):423-5. doi: 10.1016/s0091-3057(96)00221-3. PMID: 9077578.
413	9113965	Methohexital	1997	Freyer DR, Schwanda AE, Sanfilippo DJ, Hackbarth RM, Hassan NE, Kopec JS, Neirotti MT. Intravenous methohexital for brief sedation of pediatric oncology outpatients: physiologic and behavioral responses. Pediatrics. 1997 May;99(5):E8. doi: 10.1542/peds.99.5.e8. PMID: 9113965.
414	9169967	SSRI	1997	Lane R, Baldwin D. Selective serotonin reuptake inhibitor-induced serotonin syndrome: review. J Clin Psychopharmacol. 1997 Jun;17(3):208-21. doi: 10.1097/00004714-199706000-00012. PMID: 9169967.
415	9184618	Clozapine	1997	Knoll JL 4th. Clozapine-related speech disturbance. J Clin Psychiatry. 1997 May;58(5):219-20. doi: 10.4088/jcp.v58n0507c. PMID: 9184618.
416	9199024	Morphine	1997	Andersen G, Christrup LL, Sjøgren P. Morfinmetabolisme--farmakokinetik og-dynamik [Morphine metabolism--pharmacokinetics and pharmacodynamics]. Ugeskr Laeger. 1997 May 26;159(22):3383-6. Danish. PMID: 9199024.
417	9213080	Tricyclic antidepressant	1997	Vandel P, Bonin B, Leveque E, Sechter D, Bizouard P. Tricyclic antidepressant-induced extrapyramidal side effects. Eur Neuropsychopharmacol. 1997 Aug;7(3):207-12. doi: 10.1016/s0924-977x(97)00405-7. PMID: 9213080.
418	9223843	Strychnine	1997	Hagen N, Swanson R. Strychnine-like multifocal myoclonus and seizures in extremely high-dose opioid administration: treatment strategies. J Pain Symptom Manage. 1997 Jul;14(1):51-8. doi: 10.1016/S0885-3924(97)00001-8. PMID: 9223843.
419	9244837	Morphine	1997	Radbruch L, Zech D, Grond S. Myoklonien als Folge von hochdosierter epiduraler und intravenöser Morphininfusion [Myoclonus resulting from high-dose epidural and intravenous morphine infusion]. Med Klin (Munich). 1997 May 15;92(5):296-9. German. doi: 10.1007/BF03045086. PMID: 9244837.
420	9248346	Lithium	1997	Takahashi M, Hashimoto S, Suenaga T, Nakamura M, Takahashi K. [Creutzfeldt-Jakob like syndrome due to lithium intoxication--a case report]. Rinsho Shinkeigaku. 1997 Apr;37(4):338-40. Japanese. PMID: 9248346.
421	9251092	Imipenem	1997	Frucht S, Eidelberg D. Imipenem-induced myoclonus. Mov Disord. 1997 Jul;12(4):621-2. doi: 10.1002/mds.870120430. PMID: 9251092.
422	9251093	Fluoxetine	1997	Ghika-Schmid F, Ghika J, Vuadens P, Bogousslavsky J, Regli F, Despland PA. Acute reversible myoclonic encephalopathy associated with fluoxetine therapy. Mov Disord. 1997 Jul;12(4):622-3. doi: 10.1002/mds.870120431. PMID: 9251093.
423	9324507	Propofol	1997	Pablo LS, Bailey JE, Ko JC. Median effective dose of propofol required for induction of anaesthesia in goats. J Am Vet Med Assoc. 1997 Jul 1;211(1):86-8. PMID: 9324507.
424	9404234	Opioid	1997	Ahmedzai S. New approaches to pain control in patients with cancer. Eur J Cancer. 1997 Jul;33 Suppl 6:S8-14. doi: 10.1016/s0959-8049(97)00205-0. PMID: 9404234.
425	9477147	Lamotrigine	1998	Wallace SJ. Myoclonus and epilepsy in childhood: a review of treatment with valproate, ethosuximide, lamotrigine and zonisamide. Epilepsy Res. 1998 Jan;29(2):147-54. doi: 10.1016/s0920-1211(97)00080-6. PMID: 9477147.

426	9489553	Tacrine	1998	Abilleira S, Viguera ML, Miquel F. Myoclonus induced by tacrine. J Neurol Neurosurg Psychiatry. 1998 Feb;64(2):281. doi: 10.1136/jnnp.64.2.281. PMID: 9489553; PMCID: PMC2169942.
427	9514554	Opioid	1998	Mercadante S. Pathophysiology and treatment of opioid-related myoclonus in cancer patients. Pain. 1998 Jan;74(1):5-9. PMID: 9514554.
428	9562139	Venlafaxine, fluoxetine	1998	Bhatara VS, Magnus RD, Paul KL, Preskorn SH. Serotonin syndrome induced by venlafaxine and fluoxetine: a case study in polypharmacy and potential pharmacodynamic and pharmacokinetic mechanisms. Ann Pharmacother. 1998 Apr;32(4):432-6. doi: 10.1345/aph.17041. PMID: 9562139.
429	9564111	Opioid	1998	Twycross R. Re: Opioid-induced myoclonus and seizures. J Pain Symptom Manage. 1998 Mar;15(3):143-4. doi: 10.1016/s0885-3924(97)00359-x. PMID: 9564111.
430	9579275	Propofol, fentanyl	1998	Kiyama S, Yoshikawa T. Persistent intraoperative myoclonus during propofol-fentanyl anaesthesia. Can J Anaesth. 1998 Mar;45(3):283-4. doi: 10.1007/BF03012925. PMID: 9579275.
431	9579909	Carbamazepine, phenobarbital, clobazam, valproate	1997	Gambardella A, Aguglia U, Oliveri RL, Russo C, Zappia M, Quattrone A. Negative myoclonic status due to antiepileptic drug tapering: report of three cases. Epilepsia. 1997 Jul;38(7):819-23. doi: 10.1111/j.1528-1157.1997.tb01469.x. PMID: 9579909.
432	9608225	Prednimustine	1998	Ezpeleta Echávarri D, Muñoz-Blanco JL, Diaz-Otero F, Giménez Roldán S. Mioclonias inducidas por prednimustina [Myoclonus induced by prednimustine]. Neurologia. 1998 Mar;13(3):154-5. Spanish. PMID: 9608225.
433	9613750	Levodopa	1998	Caviness JN, Adler CH, Newman S, Caselli RJ, Muenter MD. Cortical myoclonus in levodopa-responsive parkinsonism. Mov Disord. 1998 May;13(3):540-4. doi: 10.1002/mds.870130327. PMID: 9613750.
434	9631427	Gallamine	1998	Ramzan I, Goldsmith R, Burke W. A novel substance associated with gallamine-induced myoclonus. Neuroreport. 1998 May 11;9(7):1349-52. doi: 10.1097/00001756-199805110-00018. PMID: 9631427.
435	9633748	Cefuroxime	1998	Herishanu YO, Zlotnik M, Mostoslavsky M, Podgaietski M, Frisher S, Wirguin I. Cefuroxime-induced encephalopathy. Neurology. 1998 Jun;50(6):1873-5. doi: 10.1212/wnl.50.6.1873. PMID: 9633748.
436	9640838	Traditional medicine	1998	Adamolekun B, Hakim JG. Opsoclonus-myoclonus associated with traditional medicine ingestion: case report. East Afr Med J. 1998 Feb;75(2):120-1. PMID: 9640838.
437	9650847	5-hydroxytryptophan	1998	Pappert EJ, Goetz CG, Stebbins GT, Belden M, Carvey PM. 5-Hydroxytryptophan-induced myoclonus in guinea pigs: mediation through 5-HT1/2 receptor subtypes. Eur J Pharmacol. 1998 Apr 17;347(1):51-6. doi: 10.1016/s0014-2999(98)00086-7. PMID: 9650847.
438	9696181	Moclobemide, paroxetine, sertraline, venlafaxine	1998	Graudins A, Stearman A, Chan B. Treatment of the serotonin syndrome with cyproheptadine. J Emerg Med. 1998 Jul-Aug;16(4):615-9. doi: 10.1016/s0736-4679(98)00057-2. PMID: 9696181.
439	9714427	Hydromorphone	1998	Wright AW, Nocente ML, Smith MT. Hydromorphone-3-glucuronide: biochemical synthesis and preliminary pharmacological evaluation. Life Sci. 1998;63(5):401-11. doi: 10.1016/s0024-3205(98)00288-4. PMID: 9714427.
440	9763378	Gadodiamide	1998	Ray DE, Holton JL, Nolan CC, Cavanagh JB, Harpur ES. Neurotoxic potential of gadodiamide after injection into the lateral cerebral ventricle of rats. AJNR Am J Neuroradiol. 1998 Sep;19(8):1455-62. PMID: 9763378; PMCID: PMC8338687.
441	9776334	Carbamazepine	1998	Genton P, Nguyen VH, Mesdjian E. Carbamazepine intoxication with negative myoclonus after the addition of clobazam. Epilepsia. 1998 Oct;39(10):1115-8. doi: 10.1111/j.1528-1157.1998.tb01299.x. PMID: 9776334.
442	9776967	Carisoprodol	1998	Roth BA, Vinson DR, Kim S. Carisoprodol-induced myoclonic encephalopathy. J Toxicol Clin Toxicol. 1998;36(6):609-12. doi: 10.3109/15563659809028058. PMID: 9776967.
443	9793810	Methohexital	1998	Khalil SN, Howard G, Mankarious R, Campos C, Kellaway J, Chuang AZ, Ruiz R. Alfentanil decreases the excitatory phenomena of sodium methohexital. J Clin Anesth. 1998 Sep;10(6):469-73. doi: 10.1016/s0952-8180(98)00072-5. PMID: 9793810.
444	9809090	Morphine	1998	Sjøgren P, Thunedborg LP, Christrup L, Hansen SH, Franks J. Is development of hyperalgesia, allodynia and myoclonus related to morphine metabolism during long-term administration? Six case histories. Acta Anaesthesiol Scand. 1998 Oct;42(9):1070-5. doi: 10.1111/j.1399-6576.1998.tb05378.x. PMID: 9809090.
445	9815948	Pyrazoloacridine	1995	LoRusso P, Foster BJ, Poplin E, McCormick J, Kraut M, Flaherty L, Heilbrun LK, Valdivieso M, Baker L. Phase I clinical trial of pyrazoloacridine NSC366140 (PD115934). Clin Cancer Res. 1995 Dec;1(12):1487-93. PMID: 9815948.
446	9915320	Etomidate	1999	Doenicke AW, Roizen MF, Kugler J, Kroll H, Foss J, Ostwald P. Reducing myoclonus after etomidate. Anesthesiology. 1999 Jan;90(1):113-9. doi: 10.1097/00000542-199901000-00017. PMID: 9915320.
447	9921884	Lithium, TCA	1999	Evidente VG, Caviness JN. Focal cortical transient preceding myoclonus during lithium and tricyclic antidepressant therapy. Neurology. 1999 Jan 1;52(1):211-3. doi: 10.1212/wnl.52.1.211. PMID: 9921884.
448	10026686	Opioid	1999	Ashby MA, Martin P, Jackson KA. Opioid substitution to reduce adverse effects in cancer pain management. Med J Aust. 1999 Jan 18;170(2):68-71. doi: 10.5694/j.1326-5377.1999.tb126885.x. PMID: 10026686.
449	10049986	Propofol	1999	Hertzog JH, Campbell JK, Dalton HJ, Hauser GJ. Propofol anesthesia for invasive procedures in ambulatory and hospitalized children: experience in the pediatric intensive care unit. Pediatrics. 1999 Mar;103(3):E30. doi: 10.1542/peds.103.3.e30. PMID: 10049986.
450	10052540	Imipenem/cilastatin	1999	Rivera M, Crespo M, Teruel JL, Marcén R, Ortuño J. Neurotoxicity due to imipenem/cilastatin in patients on continuous ambulatory peritoneal dialysis. Nephrol Dial Transplant. 1999 Jan;14(1):258-9. doi: 10.1093/ndt/14.1.258. PMID: 10052540.
451	10095385	SSRIs	1999	Apelland T, Gedde-Dahl T, Dietrichson T. Serotonergt syndrom med dødelig utgang utløst av selektiv serotoninreopptakshemmer [Serotonin syndrome with fatal outcome caused by selective serotonin reuptake inhibitors]. Tidsskr Nor Laegeforen. 1999 Feb 20;119(5):647-50. Norwegian. PMID: 10095385.
452	10202603	Hydrocodone	1999	Lauterbach EC. Hiccup and apparent myoclonus after hydrocodone: review of the opiate-related hiccup and myoclonus literature.

				Clin Neuropharmacol. 1999 Mar-Apr;22(2):87-92. doi: 10.1097/00002826-199903000-00004. PMID: 10202603.
453	10222608	Clozapine, lithium	1999	Lee SH, Yang YY. Reversible neurotoxicity induced by a combination of clozapine and lithium: a case report. Zhonghua Yi Xue Za Zhi (Taipei). 1999 Mar;62(3):184-7. PMID: 10222608.
454	10365727	Morphine	1999	Ferris DJ. Controlling myoclonus after high-dosage morphine infusions. Am J Health Syst Pharm. 1999 May 15;56(10):1009-10. doi: 10.1093/ajhp/56.10.1009. PMID: 10365727.
455	10388331	Levofloxacin	1999	Yasuda H, Yoshida A, Masuda Y, Fukayama M, Kita Y, Inamatsu T. [Levofloxacin-induced neurological adverse effects such as convulsion, involuntary movement (tremor, myoclonus and chorea like), visual hallucination in two elderly patients]. Nihon Ronen Igakkai Zasshi. 1999 Mar;36(3):213-7. Japanese. doi: 10.3143/geriatrics.36.213. PMID: 10388331.
456	10391073	Interferon-alpha	1999	Kano S, Kamoshita H, Kawaguchi N, Asahina M, Hirayama K. [Spinal myoclonus which appeared during the administration of alpha interferon]. Rinsho Shinkeigaku. 1999 Feb-Mar;39(2-3):316-20. Japanese. PMID: 10391073.
457	10429488	Carbamazepine	1999	Go T. [Carbamazepine-induced involuntary movements in a girl with localization-related epilepsy]. No To Hattatsu. 1999 Jul;31(4):366-9. Japanese. PMID: 10429488.
458	10435516	Carvedilol	1999	Fernandez HH, Friedman JH. Carvedilol-induced myoclonus. Mov Disord. 1999 Jul;14(4):703. doi: 10.1002/1531-8257(199907)14:4<703::aid-mds1029>3.0.co;2-g. PMID: 10435516.
459	10472509	Morphine	1999	Advokat C, Duke M. Comparison of morphine-induced effects on thermal nociception, mechanoreception, and hind limb flexion in chronic spinal rats. Exp Clin Psychopharmacol. 1999 Aug;7(3):219-25. doi: 10.1037//1064-1297.7.3.219. PMID: 10472509.
460	10478357	Isoniazid	1999	Ikeda K, Iwasaki Y, Ichikawa Y, Kinoshita M. [A case of transient isoniazid-induced myoclonic seizures]. No To Shinkei. 1999 Aug;51(8):717-21. Japanese. PMID: 10478357.
461	10487478	Lamotrigine	1999	Guerrini R, Belmonte A, Parmeggiani L, Perucca E. Myoclonic status epilepticus following high-dosage lamotrigine therapy. Brain Dev. 1999 Sep;21(6):420-4. doi: 10.1016/s0387-7604(99)00048-0. PMID: 10487478.
462	10502993	Epidural block	1999	Ogata K, Yamada T, Yoshimura T, Taniwaki T, Kira J. [A case of spinal myoclonus associated with epidural block for lumbago]. Rinsho Shinkeigaku. 1999 Jun;39(6):658-60. Japanese. PMID: 10502993.
463	10513376	Etomidate	1999	Khalil SN, Lawson KS, Hanis CL, Lemak NA, Ruiz RS. Alfentanil decreases myoclonus caused by etomidate. Middle East J Anaesthesiol. 1999 Jun;15(2):185-92. PMID: 10513376.
464	10513696	Carbamazepine	1999	Nanba Y, Maegaki Y. Epileptic negative myoclonus induced by carbamazepine in a child with BECTS. Benign childhood epilepsy with centrotemporal spikes. Pediatr Neurol. 1999 Sep;21(3):664-7. doi: 10.1016/s0887-8994(99)00054-5. PMID: 10513696.
465	10522740	Opioid	1999	Daeninck PJ, Bruera E. Opioid use in cancer pain. Is a more liberal approach enhancing toxicity? Acta Anaesthesiol Scand. 1999 Oct;43(9):924-38. doi: 10.1034/j.1399-6576.1999.430910.x. PMID: 10522740.
466	10529539	Carbon monoxide	1999	Choi IS, Cheon HY. Delayed movement disorders after carbon monoxide poisoning. Eur Neurol. 1999;42(3):141-4. doi: 10.1159/000008088. PMID: 10529539.
467	10547561	Opioid	1999	Mercadante S. Opioid rotation for cancer pain: rationale and clinical aspects. Cancer. 1999 Nov 1;86(9):1856-66. doi: 10.1002/(sici)1097-0142(19991101)86:9<1856::aid-cncr30>3.0.co;2-g. PMID: 10547561.
468	10586563	Epidural anesthesia	1999	Nakatani K, Nakafusa J, Katekawa Y, Chinen K. [Spinal myoclonus possibly caused by epidural anesthesia]. Masui. 1999 Nov;48(11):1248-9. Japanese. PMID: 10586563.
469	10709455	Tramadol, iproniazid	1999	de Larquier A, Vial T, Bréjoux G, Descotes J. Syndrome sérotoninergique lors de l'association tramadol et iproniazide [Serotoninergic syndrome after combining tramadol and iproniazid]. Therapie. 1999 Nov-Dec;54(6):767-8. French. PMID: 10709455.
470	10744432	Clomipramine	1999	Bloem BR, Lammers GJ, Roofthooft DW, De Beaufort AJ, Brouwer OF. Clomipramine withdrawal in newborns. Arch Dis Child Fetal Neonatal Ed. 1999 Jul;81(1):F77. doi: 10.1136/fn.81.1.f77a. PMID: 10744432; PMCID: PMC1720967.
471	10751931	Paroxetine	1999	Cavallazzi LO, Grezesiuk AK. Síndrome serotoninérgica associada ao uso de paroxetina. Relato de caso [Serotonin syndrome associated to the use of paroxetine. Case report]. Arq Neuropsiquiatr. 1999 Sep;57(3B):886-9. Portuguese. doi: 10.1590/s0004-282x1999000500027. PMID: 10751931.
472	10756416	Gabapentin	2000	Asconapé J, Diedrich A, DellaBadia J. Myoclonus associated with the use of gabapentin. Epilepsia. 2000 Apr;41(4):479-81. doi: 10.1111/j.1528-1157.2000.tb00192.x. PMID: 10756416.
473	10762127	Levodopa	2000	Fahn S. The spectrum of levodopa-induced dyskinesias. Ann Neurol. 2000 Apr;47(4 Suppl 1):S2-9; discussion S9-11. PMID: 10762127.
474	10764909	Ricinine	2000	Ferraz AC, Pereira LF, Ribeiro RL, Wolfman C, Medina JH, Scorza FA, Santos NF, Cavalheiro EA, Da Cunha C. Ricinine-elicited seizures. A novel chemical model of convulsive seizures. Pharmacol Biochem Behav. 2000 Apr;65(4):577-83. doi: 10.1016/s0091-3057(99)00250-6. PMID: 10764909.
475	10767872	Carbamazepine	2000	Rösel T, Schneider J, Fischer JT, Druschky KF. "Carbamazepin-Intoxikation" durch Verwechslung von Medikamenten [Carbamazepine poisoning caused by confusing the drugs]. Dtsch Med Wochenschr. 2000 Mar 24;125(12):352-6. German. doi: 10.1055/s-2007-1024179. PMID: 10767872.
476	10803798	Lamotrigine	2000	Janszky J, Rásonyi G, Halász P, Olajos S, Perényi J, Szûcs A, Debreczeni T. Disabling erratic myoclonus during lamotrigine therapy with high serum level--report of two cases. Clin Neuropharmacol. 2000 Mar-Apr;23(2):86-9. doi: 10.1097/00002826-200003000-00005. PMID: 10803798.
477	10818650	Antidepressants	1999	Mackay FJ, Dunn NR, Mann RD. Antidepressants and the serotonin syndrome in general practice. Br J Gen Pract. 1999 Nov;49(448):871-4. PMID: 10818650; PMCID: PMC1313555.
478	10822452	Albuterol	2000	Mícheli F, Cersósimo MG, Scorticati MC, Velez M, Gonzalez S. Myoclonus secondary to albuterol (salbutamol) instillation. Neurology. 2000 May 23;54(10):2022-3. doi: 10.1212/wnl.54.10.2022. PMID: 10822452.

479	10845207	Toluene	2000	Sugiyama-Oishi A, Arakawa K, Araki E, Yamada T, Tobimatsu S, Kira J. [A case of chronic toluene intoxication presenting stimulus-sensitive segmental spinal myoclonus]. No To Shinkei. 2000 May;52(5):399-403. Japanese. PMID: 10845207.
480	10874511	Morphine, hydromorphone	2000	Smith MT. Neuroexcitatory effects of morphine and hydromorphone: evidence implicating the 3-glucuronide metabolites. Clin Exp Pharmacol Physiol. 2000 Jul;27(7):524-8. doi: 10.1046/j.1440-1681.2000.03290.x. PMID: 10874511.
481	10888415	Opioid	2000	Mancini I, Lossignol DA, Body JJ. Opioid switch to oral methadone in cancer pain. Curr Opin Oncol. 2000 Jul;12(4):308-13. doi: 10.1097/00001622-200007000-00006. PMID: 10888415.
482	10917413	Olanzapine	2000	Nayudu SK, Scheftner WA. Case report of withdrawal syndrome after olanzapine discontinuation. J Clin Psychopharmacol. 2000 Aug;20(4):489-90. doi: 10.1097/00004714-200008000-00016. PMID: 10917413.
483	10928399	Buspirone, fluoxetine	2000	Manos GH. Possible serotonin syndrome associated with buspirone added to fluoxetine. Ann Pharmacother. 2000 Jul-Aug;34(7-8):871-4. doi: 10.1345/aph.19341. PMID: 10928399.
484	10941349	Clomipramine, moclobemide, imipramine, phenelzine, tranylcypromine, nortriptyline, desipramine, olanzapine, paroxetine, sertraline, citalopram, venlafazine, nefazodone, trazodone, lithium, valproate, dextromethorphan, sumatriptan, ayahuasca	2000	Mason PJ, Morris VA, Balcezak TJ. Serotonin syndrome. Presentation of 2 cases and review of the literature. Medicine (Baltimore). 2000 Jul;79(4):201-9. doi: 10.1097/00005792-200007000-00001. PMID: 10941349.
485	10960295	Fentanyl	2000	Stuerenburg HJ, Claassen J, Eggers C, Hansen HC. Acute adverse reaction to fentanyl in a 55 year old man. J Neurol Neurosurg Psychiatry. 2000 Aug;69(2):281-2. doi: 10.1136/jnnp.69.2.281. PMID: 10960295; PMCID: PMC1737049.
486	11030457	Phenytoin	2000	Chi WM, Chua KS, Kong KH. Phenytoin-induced asterixis--uncommon or under-diagnozed? Brain Inj. 2000 Sep;14(9):847-50. doi: 10.1080/026990500421949. PMID: 11030457.
487	11054208	Sertraline	2000	Brendel DH, Bodkin JA, Yang JM. Massive sertraline overdose. Ann Emerg Med. 2000 Nov;36(5):524-6. doi: 10.1067/mem.2000.111575. PMID: 11054208.
488	11062861	Urea	2000	Nanri M, Yamamoto A, Matsuura N. [Effect of piracetam on urea-induced myoclonus in rats]. Nihon Shinkei Seishin Yakurigaku Zasshi. 2000 May;20(2):45-50. Japanese. PMID: 11062861.
489	11136141	Etomidate	2001	Ruth WJ, Burton JH, Bock AJ. Intravenous etomidate for procedural sedation in emergency department patients. Acad Emerg Med. 2001 Jan;8(1):13-8. doi: 10.1111/j.1553-2712.2001.tb00539.x. PMID: 11136141.
490	11143506	Vigabatrin	2000	García Pastor A, García-Zarza E, Peraita Adrados R. Encefalopatía aguda y estado de mal mioclónico inducidos por vigabatrina en monoterapia [[Acute encephalopathy and myoclonic status induced by vigabatrin monotherapy]]. Neurologia. 2000 Oct;15(8):370-4. Spanish. PMID: 11143506.
491	11160978	Amantadine	2001	Matsunaga K, Uozumi T, Qingrui L, Hashimoto T, Tsuji S. Amantadine-induced cortical myoclonus. Neurology. 2001 Jan 23;56(2):279-80. doi: 10.1212/wnl.56.2.279. PMID: 11160978.
492	11191931	Cefepime	2000	Jallon P, Fankhauser L, Du Pasquier R, Coeytaux A, Picard F, Hefft S, Assal F. Severe but reversible encephalopathy associated with cefepime. Neurophysiol Clin. 2000 Dec;30(6):383-6. doi: 10.1016/s0987-7053(00)00234-3. PMID: 11191931.
493	11198052	Clozapine	2000	Aitchison KJ, Jann MW, Zhao JH, Sakai T, Zaher H, Wolff K, Collier DA, Kerwin RW, Gonzalez FJ. Clozapine pharmacokinetics and pharmacodynamics studied with Cyp1A2-null mice. J Psychopharmacol. 2000;14(4):353-9. doi: 10.1177/026988110001400403. PMID: 11198052.
494	11217651	Cefepime	2000	Saurina A, Vera M, Pou M, López Pedret J, Darnell A, Campistol JM, Cases A. Estatus epiléptico no convulsivo secundario a cefepima a dosis ajustadas en enfermos con insuficiencia renal crónica [Non-convulsive status epilepticus secondary to adjusted cefepime doses in patients with chronic renal failure]. Nefrologia. 2000 Nov-Dec;20(6):554-8. Spanish. PMID: 11217651.
495	11271983	Opioid	2001	O'Mahony S, Coyle N, Payne R. Current management of opioid-related side effects. Oncology (Williston Park). 2001 Jan;15(1):61-73, 77; discussion 77-8, 80-2. PMID: 11271983.
496	11310291	Phenytoin	2001	Miralles A, Vivancos F, Iváñez V, Arpa J, Barreiro P. Encefalopatía aguda con mioclonías por fenitoína. A propósito de un caso [Acute encephalopathy with myoclonus caused by phenytoin. Apropos of a case]. Rev Neurol. 2001 Feb 1-15;32(3):298-9. Spanish. PMID: 11310291.
497	11325416	Pentylenetetrazol	2001	Medina AE, Manhães AC, Schmidt SL. Sex differences in sensitivity to seizures elicited by pentylenetetrazol in mice. Pharmacol Biochem Behav. 2001 Mar;68(3):591-6. doi: 10.1016/s0091-3057(01)00466-x. PMID: 11325416.
498	11331334	Morphine	2001	Cherny N, Ripamonti C, Pereira J, Davis C, Fallon M, McQuay H, Mercadante S, Pasternak G, Ventafridda V; Expert Working Group of the European Association of Palliative Care Network. Strategies to manage the adverse effects of oral morphine: an evidence-based report. J Clin Oncol. 2001 May 1;19(9):2542-54. doi: 10.1200/JCO.2001.19.9.2542. PMID: 11331334.
499	11349753	Midazolam	2001	Zaw W, Knoppert DC, da Silva O. Flumazenil's reversal of myoclonic-like movements associated with midazolam in term newborns. Pharmacotherapy. 2001 May;21(5):642-6. doi: 10.1592/phco.21.6.642.34545. PMID: 11349753.
500	11401105	Opiod	2001	Mercadante S, Villari P, Fulfaro F. Gabapentin for opiod-related myoclonus in cancer patients. Support Care Cancer. 2001 May;9(3):205-6. doi: 10.1007/s005200000230. PMID: 11401105.
501	11406880	Methadone	2001	Sarhill N, Davis MP, Walsh D, Nouneh C. Methadone-induced myoclonus in advanced cancer. Am J Hosp Palliat Care. 2001 Jan-Feb;18(1):51-3. doi: 10.1177/104990910101800113. PMID: 11406880.

502	11419238	Sevoflurane	2001	Conreux F, Best O, Preckel MP, Lhopitault C, Beydon L, Pouplard F, Granry JC. Effets électroencéphalographiques du sévoflurane à l'induction chez le jeune enfant: étude prospective sur 20 cas [Electroencephalographic effects of sevoflurane in pediatric anesthesia: a prospective study of 20 cases]. Ann Fr Anesth Reanim. 2001 May;20(5):438-45. French. doi: 10.1016/s0750-7658(01)00393-8. PMID: 11419238.
503	11422338	Pregabalin	2001	Huppertz HJ, Feuerstein TJ, Schulze-Bonhage A. Myoclonus in epilepsy patients with anticonvulsive add-on therapy with pregabalin. Epilepsia. 2001 Jun;42(6):790-2. doi: 10.1046/j.1528-1157.2001.44000.x. PMID: 11422338.
504	11433583	Bismuth	2001	Bouchon JP, Cottin-Bouchon D. Une mamie pas si folle que cela.... intoxication au bismuth [A dear old lady not so crazy as this....bismuth poisoning]. Rev Med Interne. 2001 Jun;22 Suppl 2:245s-246s. French. doi: 10.1016/s0248-8663(01)83661-4. PMID: 11433583.
505	11435808	Carbamazepine	2001	De Rubeis DA, Young GB. Continuous EEG monitoring in a patient with massive carbamazepine overdose. J Clin Neurophysiol. 2001 Mar;18(2):166-8. doi: 10.1097/00004691-200103000-00008. PMID: 11435808.
506	11436961	Sertraline	2001	Ghaziuddin N, Iqbal A, Khetarpal S. Myoclonus during prolonged treatment with sertraline in an adolescent patient. J Child Adolesc Psychopharmacol. 2001 Summer;11(2):199-202. doi: 10.1089/104454601750284126. PMID: 11436961.
507	11455177	Entacapone	2001	Onofrj M, Thomas A, Iacono D, Di Iorio A, Bonanni L. Switch-over from tolcapone to entacapone in severe Parkinson's disease patients. Eur Neurol. 2001;46(1):11-6. doi: 10.1159/000050749. PMID: 11455177.
508	11459432	Hydromorphone	2001	Wright AW, Mather LE, Smith MT. Hydromorphone-3-glucuronide: a more potent neuro-excitant than its structural analogue, morphine-3-glucuronide. Life Sci. 2001 Jun 15;69(4):409-20. doi: 10.1016/s0024-3205(01)01133-x. PMID: 11459432.
509	11471277	Chloralose	2001	Hamouda C, Amamou M, Jédidi S, Hédhili A, Salah NB, Thabet H. Classification en grades des intoxications aiguës par le chloralose. 509 cas [Graded classification of acute chloralose poisoning. 509 cases]. Presse Med. 2001 Jun 16-23;30(21):1055-8. French. PMID: 11471277.
510	11474735	Trazodone, fluoxetine	2001	Darko W, Guharoy R, Rose F, Lehman D, Pappas V. Myoclonus secondary to the concurrent use of trazodone and fluoxetine. Vet Hum Toxicol. 2001 Aug;43(4):214-5. PMID: 11474735.
511	11483404	Cobalamin	2001	Ozer EA, Turker M, Bakiler AR, Yaprak I, Ozturk C. Involuntary movements in infantile cobalamin deficiency appearing after treatment. Pediatr Neurol. 2001 Jul;25(1):81-3. doi: 10.1016/s0887-8994(01)00289-2. PMID: 11483404.
512	11498064	Cephalosporins	2001	Martínez-Rodríguez JE, Barriga FJ, Santamaria J, Iranzo A, Pareja JA, Revilla M, dela Rosa CR. Nonconvulsive status epilepticus associated with cephalosporins in patients with renal failure. Am J Med. 2001 Aug;111(2):115-9. doi: 10.1016/s0002-9343(01)00767-7. PMID: 11498064.
513	11506132	Opioid	2001	McClain BC, Probst LA, Pinter E, Hartmannsgruber M. Intravenous clonidine use in a neonate experiencing opioid-induced myoclonus. Anesthesiology. 2001 Aug;95(2):549-50. doi: 10.1097/00000542-200108000-00042. PMID: 11506132.
514	11575986	Gatifloxacin	2001	Marinella MA. Myoclonus and generalized seizures associated with gatifloxacin treatment. Arch Intern Med. 2001 Oct 8;161(18):2261-2. doi: 10.1001/archinte.161.18.2261. PMID: 11575986.
515	11579867	Verapamil	2001	Maiteh M, Daoud AS. Myoclonic seizure following intravenous verapamil injection: case report and review of the literature. Ann Trop Paediatr. 2001 Sep;21(3):271-2. doi: 10.1080/02724930120077862. PMID: 11579867.
516	11743974	Nickel	2001	Cooper RM, Legare CE, Campbell Teskey G. Changes in (14)C-labeled 2-deoxyglucose brain uptake from nickel-induced epileptic activity. Brain Res. 2001 Dec 27;923(1-2):71-81. doi: 10.1016/s0006-8993(01)03034-7. PMID: 11743974.
517	11758001	Antidepressant	2001	Whyte IM, Isbister GK. Misdiagnosis of myoclonus in antidepressant induced serotonin excess. Vet Hum Toxicol. 2001 Dec;43(6):375-6. PMID: 11758001.
518	11779671	Fentanyl	2002	Han PK, Arnold R, Bond G, Janson D, Abu-Elmagd K. Myoclonus secondary to withdrawal from transdermal fentanyl: case report and literature review. J Pain Symptom Manage. 2002 Jan;23(1):66-72. doi: 10.1016/s0885-3924(01)00370-0. PMID: 11779671.
519	11879291	Opioid	2001	Stanley KJ. A patient with renal cell carcinoma, myoclonus, and unrelieved pain. Cancer Pract. 2001 May-Jun;9(3):114-8. doi: 10.1046/j.1523-5394.2001.009003114.x. PMID: 11879291.
520	11895057	Risperidone, fluvoxamine	2002	Reeves RR, Mack JE, Beddingfield JJ. Neurotoxic syndrome associated with risperidone and fluvoxamine. Ann Pharmacother. 2002 Mar;36(3):440-3. doi: 10.1345/aph.1A241. PMID: 11895057.
521	11914425	Verapamil	2002	Vadlamudi L, Wijdicks EF. Multifocal myoclonus due to verapamil overdose. Neurology. 2002 Mar 26;58(6):984. doi: 10.1212/wnl.58.6.984. PMID: 11914425.
522	11918514	Mirtazapine	2002	Hernández JL, Ramos FJ, Infante J, Rebollo M, González-Macías J. Severe serotonin syndrome induced by mirtazapine monotherapy. Ann Pharmacother. 2002 Apr;36(4):641-3. doi: 10.1345/aph.1A302. PMID: 11918514.
523	11949276	Lorazepam	2000	Noerr B. Lorazepam. Neonatal Netw. 2000 Dec;19(8):65-7. doi: 10.1891/0730-0832.19.8.65. PMID: 11949276.
524	12084448	Ifosfamide	2002	Meyer T, Ludolph AC, Münch C. Ifosfamide encephalopathy presenting with asterixis. J Neurol Sci. 2002 Jul 15;199(1-2):85-8. doi: 10.1016/s0022-510x(02)00077-1. PMID: 12084448.
525	12084450	Manganese	2002	Ono K, Komai K, Yamada M. Myoclonic involuntary movement associated with chronic manganese poisoning. J Neurol Sci. 2002 Jul 15;199(1-2):93-6. doi: 10.1016/s0022-510x(02)00111-9. PMID: 12084450.
526	12087322	Etomidate	2002	Bozeman WP, Young S. Etomidate as a sole agent for endotracheal intubation in the prehospital air medical setting. Air Med J. 2002 Jul-Aug;21(4):32-5; discussion 35-7. doi: 10.1067/mmj.2002.125935. PMID: 12087322.
527	12180424	Propofol	2002	Dearlove JC, Dearlove OR. Cortical reflex myoclonus after propofol anaesthesia. Anaesthesia. 2002 Aug;57(8):834-5. doi: 10.1046/j.1365-2044.2002.02752_20.x. PMID: 12180424.
528	12214012	Bismuth	1998	Summers WK. Bismuth Toxicity Masquerading as Alzheimer's Dementia. J Alzheimers Dis. 1998 Mar;1(1):57-59. doi: 10.3233/jad-1998-1104. PMID: 12214012.

529	12243607	Nefopam	2002	Villier C, Mallaret MP. Nefopam abuse. Ann Pharmacother. 2002 Oct;36(10):1564-6. doi: 10.1345/aph.1C017. PMID: 12243607.
530	12297601	Diltiazem	2002	Jeret JS. Diltiazem-induced myoclonus. Neurology. 2002 Sep 24;59(6):962; author reply 962. doi: 10.1212/wnl.59.6.962. PMID: 12297601.
531	12380661	Morphine, fentanyl	2002	McNamara P. Opioid switching from morphine to transdermal fentanyl for toxicity reduction in palliative care. Palliat Med. 2002 Sep;16(5):425-34. doi: 10.1191/0269216302pm536oa. PMID: 12380661.
532	12410053	Mefloquine	2002	Jiménez-Huete A, Gil-Nagel A, Franch O. Multifocal myoclonus associated with mefloquine chemoprophylaxis. Clin Neuropharmacol. 2002 Sep-Oct;25(5):243. doi: 10.1097/00002826-200209000-00002. PMID: 12410053.
533	12465085	Clozapine	2002	Zesiewicz TA, Borra S, Hauser RA. Clozapine withdrawal symptoms in a Parkinson's disease patient. Mov Disord. 2002 Nov;17(6):1365-7. doi: 10.1002/mds.10282. PMID: 12465085.
534	12475194	Bromomethane (methyl bromide)	2002	Hoizey G, Souchon PF, Trenque T, Frances C, Lamiable D, Nicolas A, Grossenbacher F, Sabouraud P, Bednarek N, Motte J, Millart H. An unusual case of methyl bromide poisoning. J Toxicol Clin Toxicol. 2002;40(6):817-21. doi: 10.1081/clt-120015841. PMID: 12475194.
535	12522612	Contrast agent	2002	van der Leede H, Jorens PG, Parizel P, Cras P. Inadvertent intrathecal use of ionic contrast agent. Eur Radiol. 2002 Dec;12 Suppl 3:S86-93. doi: 10.1007/s00330-002-1417-z. Epub 2002 Jun 4. PMID: 12522612.
536	12549949	Venlafaxine	2003	Pan JJ, Shen WW. Serotonin syndrome induced by low-dose venlafaxine. Ann Pharmacother. 2003 Feb;37(2):209-11. doi: 10.1177/106002800303700209. PMID: 12549949.
537	12565191	Morphine	2003	Andersen G, Christrup L, Sjøgren P. Relationships among morphine metabolism, pain and side effects during long-term treatment: an update. J Pain Symptom Manage. 2003 Jan;25(1):74-91. doi: 10.1016/s0885-3924(02)00531-6. PMID: 12565191.
538	12574010	Etomidate	2003	Guldner G, Schultz J, Sexton P, Fortner C, Richmond M. Etomidate for rapid-sequence intubation in young children: hemodynamic effects and adverse events. Acad Emerg Med. 2003 Feb;10(2):134-9. doi: 10.1111/j.1553-2712.2003.tb00030.x. PMID: 12574010.
539	12577166	Ecstasy	2003	Hinkelbein J, Gabel A, Volz M, Ellinger K. Suizidale hochdosierte Ecstasy-Einnahme [Suicide attempt with high-dose ecstasy]. Anaesthesist. 2003 Jan;52(1):51-4. German. doi: 10.1007/s00101-002-0433-3. PMID: 12577166.
540	12600808	Bismuth	2002	Teepker M, Hamer HM, Knake S, Bandmann O, Oertel WH, Rosenow F. Myoclonic encephalopathy caused by chronic bismuth abuse. Epileptic Disord. 2002 Dec;4(4):229-33. PMID: 12600808.
541	12627936	Cefepime, ceftazidime	2003	Chow KM, Szeto CC, Hui AC, Wong TY, Li PK. Retrospective review of neurotoxicity induced by cefepime and ceftazidime. Pharmacotherapy. 2003 Mar;23(3):369-73. doi: 10.1592/phco.23.3.369.32100. PMID: 12627936.
542	12633152	Lithium	2003	Caviness JN, Evidente VG. Cortical myoclonus during lithium exposure. Arch Neurol. 2003 Mar;60(3):401-4. doi: 10.1001/archneur.60.3.401. PMID: 12633152.
543	12635389	Etomidate	2003	Schwarzkopf KR, Hueter L, Simon M, Fritz HG. Midazolam pretreatment reduces etomidate-induced myoclonic movements. Anaesth Intensive Care. 2003 Feb;31(1):18-20. doi: 10.1177/0310057X0303100103. PMID: 12635389.
544	12640092	Tranexamic acid	2003	Hui AC, Wong TY, Chow KM, Szeto CC. Multifocal myoclonus secondary to tranexamic acid. J Neurol Neurosurg Psychiatry. 2003 Apr;74(4):547. doi: 10.1136/jnnp.74.4.547. PMID: 12640092; PMCID: PMC1738362.
545	12694150	Etomidate	2003	Hueter L, Schwarzkopf K, Simon M, Bredle D, Fritz H. Pretreatment with sufentanil reduces myoclonus after etomidate. Acta Anaesthesiol Scand. 2003 Apr;47(4):482-4. doi: 10.1034/j.1399-6576.2003.00081.x. PMID: 12694150.
546	12710093	Tandospirone	2002	Tamura N, Nakazato Y, Yamamoto T, Iwasaki S, Shimazu K. [Serotonin syndrome caused by tandospirone citrate alone]. Rinsho Shinkeigaku. 2002 Sep;42(9):892-4. Japanese. PMID: 12710093.
547	12736874	Baclofen	2003	Meythaler JM, Roper JF, Brunner RC. Cyproheptadine for intrathecal baclofen withdrawal. Arch Phys Med Rehabil. 2003 May;84(5):638-42. doi: 10.1016/s0003-9993(03)00105-9. PMID: 12736874.
548	12755202	Chlorambucil	2003	Benitah N, de Lorimier LP, Gaspar M, Kitchell BE. Chlorambucil-induced myoclonus in a cat with lymphoma. J Am Anim Hosp Assoc. 2003 May-Jun;39(3):283-7. doi: 10.5326/0390283. PMID: 12755202.
549	12873944	Morphine	2003	Hemstapat K, Monteith GR, Smith D, Smith MT. Morphine-3-glucuronide's neuro-excitatory effects are mediated via indirect activation of N-methyl-D-aspartic acid receptors: mechanistic studies in embryonic cultured hippocampal neurones. Anesth Analg. 2003 Aug;97(2):494-505. doi: 10.1213/01.ANE.0000059225.40049.99. PMID: 12873944.
550	12925183	Morphine	2003	Kona-Boun JJ, Pibarot P, Quesnel A. Myoclonus and urinary retention following subarachnoid morphine injection in a dog. Vet Anaesth Analg. 2003 Oct;30(4):257-64. doi: 10.1046/j.1467-2995.2003.00076.x. PMID: 12925183.
551	13246341	Chlorpromazine	1955	SIGWALD J, RAYMONDEAU C, PIOT C. Myoclonies rythmées, bilatérales et symétriques, à cadence rapide, de la moitié supérieure du corps, d'évolution subaiguë; action suspensive de la chlorpromazine et du chlorhydrate de chloro-3-(diéthylamino-3'-propyl)-10-phénothiazine [Symmetric, bilateral, rapid-rhythm myoclonus of the upper body with sub-acute course; arresting effect of chlorpromazine and of chloro-3-(diéthylamino-3'-propyl)-10-phénothiazine chlorhydrate]. Rev Neurol (Paris). 1955;92(2):89-95. French. PMID: 13246341.
552	13384320	Chlorpromazine	1956	LEROY C, SIGWALD J. Les myoclonies et épilepsies apparaissant chez certains malades au cours de l'administration de la chlorpromazine [Myoclonus and epilepsy appearing in various patients during the administration of chlorpromazine]. Encephale. 1956;45(4):904-9. French. PMID: 13384320.
553	13420533	Chloralose	1956	Bellini S. Sindrome mioclonica sperimentale da alfa-cloralosio [Experimental myoclonic syndrome induced with alpha-chloralose]. Rass Studi Psichiatr. 1956;45(5):1048-53. Italian. PMID: 13420533.
554	13439312	Chlorpromazine	1957	Blanc M, Loiseau P, Des Termes M, Staeffen J. Myoclonies au cours d'un traitement par la chlorpromazine; etude critique [Myoclonias during chlorpromazine treatment; analysis]. J Med Bord. 1957 Apr;134(4):465-7. French. PMID: 13439312.

555	14020200	Piperazine	1963	Chaptal J, Jean R, Labauge R, Bonnet H, Aghai E. [Oppositional myoclonus caused by piperazine poisoning]. Arch Fr Pediatr. 1963 Jan;20:17-23. French. PMID: 14020200.
556	14063996	Phenothiazine	1963	Eckhardt Wf Jr. Cervical-Lingual-Masticator Myoclonus; A Reaction To A Non-Halogenated Phenothiazine. Conn Med. 1963 Sep;27:561. PMID: 14063996.
557	14597801	Bupivacaine	2003	Celik Y, Bekir Demirel C, Karaca S, Kose Y. Transient segmental spinal myoclonus due to spinal anaesthesia with bupivacaine. J Postgrad Med. 2003 Jul-Sep;49(3):286. PMID: 14597801.
558	14622694	Opioid	2003	McNicol E, Horowicz-Mehler N, Fisk RA, Bennett K, Gialeli-Goudas M, Chew PW, Lau J, Carr D; Americal Pain Society. Management of opioid side effects in cancer-related and chronic noncancer pain: a systematic review. J Pain. 2003 Jun;4(5):231-56. doi: 10.1016/s1526-5900(03)00556-x. PMID: 14622694.
559	14633768	Tramadol	2003	Barrett NA, Sundaraj SR. Inadvertent intrathecal injection of tramadol. Br J Anaesth. 2003 Dec;91(6):918-20. doi: 10.1093/bja/aeg265. PMID: 14633768.
560	14652121	Etomidate	2003	Greenberg M, Hilty C. Myoclonus after prolonged infusion of etomidate treated with dantrolene. J Clin Anesth. 2003 Sep;15(6):489-90. doi: 10.1016/j.jclinane.2003.04.001. PMID: 14652121.
561	14655236	Etomidate	2003	Van Keulen SG, Burton JH. Myoclonus associated with etomidate for ED procedural sedation and analgesia. Am J Emerg Med. 2003 Nov;21(7):556-8. doi: 10.1016/j.ajem.2003.08.004. PMID: 14655236.
562	14673903	Interferon-alpha	2003	Benatru I, Thobois S, Andre-Obadia N, Gonnaud PM, Beaugendre Y, Berger C, Gonce M, Broussolle E. Atypical propriospinal myoclonus with possible relationship to alpha interferon therapy. Mov Disord. 2003 Dec;18(12):1564-8. doi: 10.1002/mds.10614. PMID: 14673903.
563	14692893	Acyclovir, valacyclovir	2004	Strumia S, De Mitri P, Bionda E. Neurotoxicity of acyclovir and valacyclovir in a hemodialyzed patient. Eur J Neurol. 2004 Jan;11(1):68-9. doi: 10.1046/j.1351-5101.2003.00719.x. PMID: 14692893.
564	14694925	Gabapentin	2003	Scullin P, Sheahan P, Sheila K. Myoclonic jerks associated with gabapentin. Palliat Med. 2003 Dec;17(8):717-8. doi: 10.1191/0269216303Pm772cr. PMID: 14694925.
565	14702438	Trimethoprim-sulfamethoxazole	2004	Dib EG, Bernstein S, Benesch C. Multifocal myoclonus induced by trimethoprim-sulfamethoxazole therapy in a patient with nocardia infection. N Engl J Med. 2004 Jan 1;350(1):88-9. doi: 10.1056/NEJM200401013500121. PMID: 14702438.
566	14706468	Carbamazepine	2004	Parmeggiani L, Seri S, Bonanni P, Guerrini R. Electrophysiological characterization of spontaneous and carbamazepine-induced epileptic negative myoclonus in benign childhood epilepsy with centro-temporal spikes. Clin Neurophysiol. 2004 Jan;115(1):50-8. doi: 10.1016/s1388-2457(03)00327-4. PMID: 14706468.
567	14731311	Clozapine, lithium	2004	Bender S, Linka T, Wolstein J, Gehendges S, Paulus HJ, Schall U, Gastpar M. Safety and efficacy of combined clozapine-lithium pharmacotherapy. Int J Neuropsychopharmacol. 2004 Mar;7(1):59-63. doi: 10.1017/S1461145703003870. Epub 2004 Jan 20. PMID: 14731311.
568	14964581	Licorice	2004	Ishiguchi T, Mikita N, Iwata T, Nakata H, Sato H, Higashimoto Y, Fujimoto H, Yoshida S, Itoh H. Myoclonus and metabolic alkalosis from licorice in antacid. Intern Med. 2004 Jan;43(1):59-62. doi: 10.2169/internalmedicine.43.59. PMID: 14964581.
569	14970364	Tramadol, venlafaxine, and mirtazapine	2004	Houlihan DJ. Serotonin syndrome resulting from coadministration of tramadol, venlafaxine, and mirtazapine. Ann Pharmacother. 2004 Mar;38(3):411-3. doi: 10.1345/aph.1D344. Epub 2004 Jan 23. PMID: 14970364.
570	14998440	Gabapentin	2004	Moretti R, Torre P, Antonello RM, Ukmar M, Cazzato G, Bava A. Gabapentin as a drug therapy of intractable hiccup because of vascular lesion: a three-year follow up. Neurologist. 2004 Mar;10(2):102-6. doi: 10.1097/01.nrl.0000117824.29975.e7. PMID: 14998440.
571	15027311	Paroxetine	2003	Ochiai Y, Katsu H, Okino S, Wakutsu N, Nakayama K. [Case of prolonged recovery from serotonin syndrome caused by paroxetine]. Seishin Shinkeigaku Zasshi. 2003;105(12):1532-8. Japanese. PMID: 15027311.
572	15037850	Lansoprazole	2004	Boulliat J, Polard E, Colin F, Bentué-Ferrer D, Allain H. Encéphalopathie myoclonique: rôle des inhibiteurs de la pompe à protons [Myoclonic encephalopathy associated with proton pump inhibitors]. Rev Neurol (Paris). 2004 Mar;160(3):350-1. French. doi: 10.1016/s0035-3787(04)70912-1. PMID: 15037850.
573	15038335	Opioid	2004	Slatkin N, Rhiner M. Treatment of opioid-induced delirium with acetylcholinesterase inhibitors: a case report. J Pain Symptom Manage. 2004 Mar;27(3):268-73. doi: 10.1016/j.jpainsymman.2003.07.002. PMID: 15038335.
574	15052466	Imipenem	2004	Lau KK, Kink RJ, Jones DP. Myoclonus associated with intraperitoneal imipenem. Pediatr Nephrol. 2004 Jun;19(6):700-1. doi: 10.1007/s00467-004-1467-3. Epub 2004 Mar 30. PMID: 15052466.
575	15102987	Dobutamine	2004	Wierre L, Decaudin B, Barsumau J, Vairon MX, Horrent S, Odou P, Azar R. Dobutamine-induced myoclonia in severe renal failure. Nephrol Dial Transplant. 2004 May;19(5):1336-7. doi: 10.1093/ndt/gfh132. PMID: 15102987.
576	15106753	Lithium	2004	Marque N, Mansencal N, Morisson-Castagnet JF, Dubourg O. Intoxication au lithium. A propos d'une observation [Acute lithium intoxication. Apropos of a case]. Arch Mal Coeur Vaiss. 2004 Mar;97(3):271-4. French. PMID: 15106753.
578	15120781	Chloralose	2004	Boyez E, Malherbe P. Curarisation en traitement symptomatique d'appoint des myoclonies induites par une intoxication par le chloralose [Curarization for contributing symptomatic treatment of myoclonic jerks induced by chloralose poisoning]. Ann Fr Anesth Reanim. 2004 Apr;23(4):361-3. French. doi: 10.1016/j.annfar.2003.11.020. PMID: 15120781.
579	15133830	Ciprofloxacin	2004	Post B, Koelman JH, Tijssen MA. Propriospinal myoclonus after treatment with ciprofloxacin. Mov Disord. 2004 May;19(5):595-7. doi: 10.1002/mds.10717. PMID: 15133830.
580	15167427	Etomidate	2004	van den Broek WW, Groenland TH, Kusuma A, Mulder PG, Bruijn JA. Double-blind placebo controlled study of the effects of etomidate-alfentanil anesthesia in electroconvulsive therapy. J ECT. 2004 Jun;20(2):107-11. doi: 10.1097/00124509-200406000-00006. PMID: 15167427.
581	15173551	Etomidate	2004	Falk J, Zed PJ. Etomidate for procedural sedation in the emergency department. Ann Pharmacother. 2004 Jul-Aug;38(7-8):1272-7. doi: 10.1345/aph.1E008. Epub 2004 Jun 1. PMID: 15173551.

582	15188079	Trimethoprim-sulfamethoxazole	2004	Jundt F, Lempert T, Dörken B, Pezzutto A. Trimethoprim-sulfamethoxazole exacerbates posthypoxic action myoclonus in a patient with suspicion of Pneumocystis jiroveci infection. Infection. 2004 Jun;32(3):176-8. doi: 10.1007/s15010-004-3011-6. PMID: 15188079.
583	15189666	Cyclonite	2003	Küçükardali Y, Acar HV, Ozkan S, Nalbant S, Yazgan Y, Atasoyu EM, Keskin O, Naz A, Akyatan N, Gökben M, Danaci M. Accidental oral poisoning caused by RDX (cyclonite): a report of 5 cases. J Intensive Care Med. 2003 Jan-Feb;18(1):42-6. doi: 10.1177/0885066602239123. PMID: 15189666.
584	15197714	Cannabis	2004	Lozsadi DA, Forster A, Fletcher NA. Cannabis-induced propriospinal myoclonus. Mov Disord. 2004 Jun;19(6):708-9. doi: 10.1002/mds.10696. PMID: 15197714.
585	15221158	Cefotiam	2004	Brössner G, Engelhardt K, Beer R, Pfausler B, Georgopoulos A, Schmutzhard E. Accidental intrathecal infusion of cefotiam: clinical presentation and management. Eur J Clin Pharmacol. 2004 Jul;60(5):373-5. doi: 10.1007/s00228-004-0787-3. Epub 2004 Jun 23. PMID: 15221158.
586	15277643	Lamotrigine	2004	Cerminara C, Montanaro ML, Curatolo P, Seri S. Lamotrigine-induced seizure aggravation and negative myoclonus in idiopathic rolandic epilepsy. Neurology. 2004 Jul 27;63(2):373-5. doi: 10.1212/01.wnl.0000130195.62670.a6. PMID: 15277643.
587	15353098	Hydromorphone	2004	Thwaites D, McCann S, Broderick P. Hydromorphone neuroexcitation. J Palliat Med. 2004 Aug;7(4):545-50. doi: 10.1089/jpm.2004.7.545. PMID: 15353098.
588	15380868	Valproic acid	2004	Reif A, Leonhard C, Mössner R, Lesch KP, Fallgatter AJ. Encephalopathy and myoclonus triggered by valproic acid. Prog Neuropsychopharmacol Biol Psychiatry. 2004 Sep;28(6):1061-3. doi: 10.1016/j.pnpbp.2004.05.041. PMID: 15380868.
589	15504714	Antiseizure medications	2004	Zaccara G, Cincotta M, Borgheresi A, Balestrieri F. Adverse motor effects induced by antiepileptic drugs. Epileptic Disord. 2004 Sep;6(3):153-68. PMID: 15504714.
590	15529307	Fentanyl, methadone	2004	Benítez-Rosario MA, Feria M, Salinas-Martín A, Martínez-Castillo LP, Martín-Ortega JJ. Opioid switching from transdermal fentanyl to oral methadone in patients with cancer pain. Cancer. 2004 Dec 15;101(12):2866-73. doi: 10.1002/cncr.20712. PMID: 15529307.
591	15562144	Quetiapine/venlafaxin e	2005	Précourt A, Dunewicz M, Grégoire G, Williamson DR. Multiple complications and withdrawal syndrome associated with quetiapine/venlafaxine intoxication. Ann Pharmacother. 2005 Jan;39(1):153-6. doi: 10.1345/aph.1E073. Epub 2004 Nov 23. PMID: 15562144.
592	15591715	5-fluorouracil	2004	Lazar A, Mau-Holzmann UA, Kolb H, Reichenmiller HE, Riess O, Schömig E. Multiple organ failure due to 5-fluorouracil chemotherapy in a patient with a rare dihydropyrimidine dehydrogenase gene variant. Onkologie. 2004 Dec;27(6):559-62. doi: 10.1159/000081338. PMID: 15591715.
593	15634624	Oxcarbazepine	2004	Hahn A, Fischenbeck A, Stephani U. Induction of epileptic negative myoclonus by oxcarbazepine in symptomatic epilepsy. Epileptic Disord. 2004 Dec;6(4):271-4. PMID: 15634624.
594	15644468	Gabapentin	2005	Pina MA, Modrego PJ. Dystonia induced by gabapentin. Ann Pharmacother. 2005 Feb;39(2):380-2. doi: 10.1345/aph.1E503. Epub 2005 Jan 11. PMID: 15644468.
595	15660783	Gabapentin	2005	Zhang C, Glenn DG, Bell WL, O'Donovan CA. Gabapentin-induced myoclonus in end-stage renal disease. Epilepsia. 2005 Jan;46(1):156-8. doi: 10.1111/j.0013-9580.2005.20804.x. PMID: 15660783.
596	15668562	Gabapentin	2005	Babiy M, Stubblefield MD, Herklotz M, Hand M. Asterixis related to gabapentin as a cause of falls. Am J Phys Med Rehabil. 2005 Feb;84(2):136-40. doi: 10.1097/01.phm.0000151943.06257.64. PMID: 15668562.
597	15673706	Dobutamine	2005	Hauben M, Reich L. Case reports of dobutamine-induced myoclonia in severe renal failure: potential of emerging pharmacovigilance technologies. Nephrol Dial Transplant. 2005 Feb;20(2):471-2. doi: 10.1093/ndt/gfh549. PMID: 15673706.
598	15707818	Sertraline	2005	Tiamfook TO, Biddinger PD, Brown DF, Nadel ES. Myoclonus and tachycardia. J Emerg Med. 2005 Feb;28(2):211-4. doi: 10.1016/j.jemermed.2004.11.004. PMID: 15707818.
599	15729090	Quetiapine	2005	Velayudhan L, Kirchner V. Quetiapine-induced myoclonus. Int Clin Psychopharmacol. 2005 Mar;20(2):119-20. doi: 10.1097/00004850-200503000-00011. PMID: 15729090.
600	15754089	Tramadol	2005	Isoardo G, Zibetti M, Troni W. Myoclonus of probable spinal origin as a potential side effect of tramadol. A case report. J Neurol. 2005 Jul;252(7):852. doi: 10.1007/s00415-005-0760-9. Epub 2005 Mar 9. PMID: 15754089.
601	15814107	Pentylenetetrazole	2005	Tirassa P, Costa N, Aloe L. CCK-8 prevents the development of kindling and regulates the GABA and NPY expression in the hippocampus of pentylenetetrazole (PTZ)-treated adult rats. Neuropharmacology. 2005 Apr;48(5):732-42. doi: 10.1016/j.neuropharm.2004.12.001. PMID: 15814107.
602	15826569	Pseudoephedrine	2005	López Lois G, Gómez Carrasco JA, García de Frías E. Reacción adversa por seudoefedrina [Adverse reaction of pseudoephedrine]. An Pediatr (Barc). 2005 Apr;62(4):378-80. Spanish. doi: 10.1157/13073253. PMID: 15826569.
603	15919102	Scopolamine	2005	Enginar N, Nurten A, Celik PY, Açikmeşe B. Scopolamine-induced convulsions in fasted mice after food intake: effects of glucose intake, antimuscarinic activity and anticonvulsant drugs. Neuropharmacology. 2005 Sep;49(3):293-9. doi: 10.1016/j.neuropharm.2005.01.032. PMID: 15919102.
604	15954908	Mushroom Sugihiratake	2005	Gejyo F, Homma N, Higuchi N, Ataka K, Teramura T, Alchi B, Suzuki Y, Nishi S, Narita I; Japanese Society of Nephrology. A novel type of encephalopathy associated with mushroom Sugihiratake ingestion in patients with chronic kidney diseases. Kidney Int. 2005 Jul;68(1):188-92. doi: 10.1111/j.1523-1755.2005.00393.x. PMID: 15954908.
605	15962861	Bupivacaine	2005	Hougaard S, Kristensen BB. Myoklonus i forbindelse med kombineret spinal-epidural anaestesi med bupivacain [Myoclonus following combined spinal-epidural anesthesia]. Ugeskr Laeger. 2005 May 23;167(21):2295. Danish. PMID: 15962861.
606	15965316	Olanzapine	2005	Camacho A, García-Navarro M, Martínez B, Villarejo A, Pomares E. Olanzapine-induced myoclonic status. Clin Neuropharmacol. 2005 May-Jun;28(3):145-7. doi: 10.1097/01.wnf.0000165351.10841.fa. PMID: 15965316.
607	16005413	Opioid	2005	Gnanadesigan N, Espinoza RT, Smith R, Israel M, Reuben DB. Interaction of serotonergic antidepressants and opioid analgesics: Is serotonin syndrome going undetected? J Am Med Dir Assoc. 2005 Jul-Aug;6(4):265-9. doi: 10.1016/j.jamda.2005.04.012.

				PMID: 16005413.
608	16115978	Etomidate	2005	Guler A, Satilmis T, Akinci SB, Celebioglu B, Kanbak M. Magnesium sulfate pretreatment reduces myoclonus after etomidate. Anesth Analg. 2005 Sep;101(3):705-709. doi: 10.1213/01.ANE.0000160529.95019.E6. PMID: 16115978.
609	16133721	Gabapentin	2006	Holtkamp M, Halle A, Meierkord H, Masuhr F. Gabapentin-induced severe myoclonus in a patient with impaired renal function. J Neurol. 2006 Mar;253(3):382-3. doi: 10.1007/s00415-005-0970-1. Epub 2005 Aug 25. PMID: 16133721.
610	16135401	Heroin (diamorphine)	2006	Dürsteler-MacFarland KM, Stohler R, Moldovanyi A, Rey S, Basdekis R, Gschwend P, Eschmann S, Rehm J. Complaints of heroin-maintained patients: A survey of symptoms ascribed to diacetylmorphine. Drug Alcohol Depend. 2006 Feb 28;81(3):231-9. doi: 10.1016/j.drugalcdep.2005.07.004. Epub 2005 Aug 30. PMID: 16135401.
611	16157917	Lamotrigine	2005	Crespel A, Genton P, Berramdane M, Coubes P, Monicard C, Baldy-Moulinier M, Gelisse P. Lamotrigine associated with exacerbation or de novo myoclonus in idiopathic generalized epilepsies. Neurology. 2005 Sep 13;65(5):762-4. doi: 10.1212/01.wnl.0000174517.21383.36. PMID: 16157917.
612	16185554	Enalapril	2005	González L, Feijóo M. Inhibidores de la enzima conversiva de la angiotensina y mioclonías [Myoclonus and angiotensin converting enzyme inhibitors]. Med Clin (Barc). 2005 Sep 24;125(10):398. Spanish. doi: 10.1157/13079177. PMID: 16185554.
613	16317595	Fluoxetine	2005	Cánovas D, Marco M, Sansa G. Mioclonías generalizadas relacionadas con la toma de fluoxetina y un anticonceptivo oral [Generalized myoclonias related with combined fluoxetine and oral contraceptive intake]. Neurologia. 2005 Dec;20(10):709. Spanish. PMID: 16317595.
614	16356781	Oxcarbazepine	2006	Kaddurah AK, Holmes GL. Possible precipitation of myoclonic seizures with oxcarbazepine. Epilepsy Behav. 2006 Feb;8(1):289-93. doi: 10.1016/j.yebeh.2005.10.007. Epub 2005 Dec 13. PMID: 16356781.
615	16379657	Ketamine, isoflurane	2005	Boscan P, Pypendop BH, Solano AM, Ilkiw JE. Cardiovascular and respiratory effects of ketamine infusions in isoflurane-anesthetized dogs before and during noxious stimulation. Am J Vet Res. 2005 Dec;66(12):2122-9. doi: 10.2460/ajvr.2005.66.2122. PMID: 16379657.
616	16459204	Pentylenetetrazol	2006	Tchekalarova J, Georgiev V. Effect of acute versus chronic theophylline administration on acute restraint stress-induced increase of pentylenetetrazol seizure threshold in mice. Brain Res Bull. 2006 Feb 15;68(6):464-8. doi: 10.1016/j.brainresbull.2005.10.004. Epub 2005 Oct 25. PMID: 16459204.
617	16519000	Propofol	2005	Nimmaanrat S. Myoclonic movements following induction of anesthesia with propofol: a case report. J Med Assoc Thai. 2005 Dec;88(12):1955-7. PMID: 16519000.
618	16548978	Etomidate	2006	Lim TA, Lim KY. BIS during etomidate-induced myoclonus. Anaesthesia. 2006 Apr;61(4):410-1. doi: 10.1111/j.1365-2044.2006.04601.x. PMID: 16548978.
619	16553519	Quetiapine	2006	Strachan PM, Benoff BA. Mental status change, myoclonus, electrocardiographic changes, and acute respiratory distress syndrome induced by quetiapine overdose. Pharmacotherapy. 2006 Apr;26(4):578-82. doi: 10.1592/phco.26.4.578. PMID: 16553519.
620	16563323	Etomidate	2006	Kelsaka E, Karakaya D, Sarihasan B, Baris S. Remifentanil pretreatment reduces myoclonus after etomidate. J Clin Anesth. 2006 Mar;18(2):83-6. doi: 10.1016/j.jclinane.2005.05.004. PMID: 16563323.
621	16567688	Cephalosporin	2006	Chan S, Turner MR, Young L, Gregory R. Cephalosporin-induced myoclonus. Neurology. 2006 Mar 28;66(6):E20. doi: 10.1212/01.wnl.0000190256.30385.13. PMID: 16567688.
622	16598342	Busulfan	2006	Denison DJ, Alghzaly AA. Busulfan induced myoclonus. Saudi Med J. 2006 Apr;27(4):557-8. PMID: 16598342.
623	16702909	Lamotrigine, escitalopram	2006	Rosenhagen MC, Schmidt U, Weber F, Steiger A. Combination therapy of lamotrigine and escitalopram may cause myoclonus. J Clin Psychopharmacol. 2006 Jun;26(3):346-7. doi: 10.1097/01.jcp.0000219927.49799.c0. PMID: 16702909.
624	16704599	Spinal-epidural anesthesia	2006	Menezes FV, Venkat N. Spinal myoclonus following combined spinal-epidural anaesthesia for Caesarean section. Anaesthesia. 2006 Jun;61(6):597-600. doi: 10.1111/j.1365-2044.2006.04655.x. PMID: 16704599.
625	16772816	Topiramate	2006	Alonso-Navarro H, Jiménez-Jiménez FJ. Reversible tremor, myoclonus, and fasciculations associated with topiramate use for migraine. Clin Neuropharmacol. 2006 May-Jun;29(3):157-9. doi: 10.1097/01.WNF.0000220825.65393.93. PMID: 16772816.
626	16844959	Hydromorphone	2006	Hofmann A, Tangri N, Lafontaine AL, Postuma RB. Myoclonus as an acute complication of low-dose hydromorphone in multiple system atrophy. J Neurol Neurosurg Psychiatry. 2006 Aug;77(8):994-5. doi: 10.1136/jnnp.2005.076588. PMID: 16844959; PMCID: PMC2077632.
627	16863493	Cefepime	2006	Lam S, Gomolin IH. Cefepime neurotoxicity: case report, pharmacokinetic considerations, and literature review. Pharmacotherapy. 2006 Aug;26(8):1169-74. doi: 10.1592/phco.26.8.1169. PMID: 16863493.
628	16914464	Etomidate	2006	Nyman Y, Von Hofsten K, Palm C, Eksborg S, Lönnqvist PA. Etomidate-Lipuro is associated with considerably less injection pain in children compared with propofol with added lidocaine. Br J Anaesth. 2006 Oct;97(4):536-9. doi: 10.1093/bja/ael187. Epub 2006 Aug 16. PMID: 16914464.
629	16916568	Paroxetine	2007	Terao T, Hikichi T. Serotonin syndrome in a case of depression with various somatic symptoms: the difficulty in differential diagnosis. Prog Neuropsychopharmacol Biol Psychiatry. 2007 Jan 30;31(1):295-6. doi: 10.1016/j.pnpbp.2006.07.007. Epub 2006 Aug 17. PMID: 16916568.
630	16960855	Metodopramide	2006	Nampiaparampil D, Oruc NE. Metodopramide-induced palatopharyngeal myoclonus. Mov Disord. 2006 Nov;21(11):2028-9. doi: 10.1002/mds.21074. PMID: 16960855.
631	16977723	NA	2006	Solberg DK, Koht J, Refsum H. En 74 år gammel bevisstløs kvinne med myoklonier og krampeanfall [A 74-year-old unconscious woman with myoclonia and seizures]. Tidsskr Nor Laegeforen. 2006 Sep 7;126(17):2275-6. Norwegian. PMID: 16977723.
632	16995352	Fluvoxamine, paroxetine	2006	Satoh K, Takano S, Onogi T, Ohtsuki K, Kobayashi T. Serotonin syndrome caused by minimum doses of SSRIS in a patient with spinal cord injury. Fukushima J Med Sci. 2006 Jun;52(1):29-33. doi: 10.5387/fms.52.29. PMID: 16995352.
633	16997421	Etomidate	2007	Miner JR, Danahy M, Moch A, Biros M. Randomized clinical trial of etomidate versus propofol for procedural sedation in the

				emergency department. Ann Emerg Med. 2007 Jan;49(1):15-22. doi: 10.1016/j.annemergmed.2006.06.042. Epub 2006 Sep 25. PMID: 16997421.
634	16997680	Etomidate	2006	Di Liddo L, D'Angelo A, Nguyen B, Bailey B, Amre D, Stanciu C. Etomidate versus midazolam for procedural sedation in pediatric outpatients: a randomized controlled trial. Ann Emerg Med. 2006 Oct;48(4):433-40, 440.e1. doi: 10.1016/j.annemergmed.2006.03.004. Epub 2006 Apr 27. PMID: 16997680.
635	16997777	Various, mainly etomidate	2006	Cole CD, Wang HE, Abo BN, Yealy DM. Drug-assisted effects on protective airway reflexes during out-of-hospital endotracheal intubation (preliminary report). Prehosp Emerg Care. 2006 Oct-Dec;10(4):472-5. doi: 10.1080/10903120600885167. PMID: 16997777.
636	17005362	Etomidate	2006	Aissaoui Y, Belyamani L, El Wali A, Idrissi Hajjouji SM, Atmani M, Drissi Kamili N. Prévention des myoclonies induites par l'étomidate en utilisant une priming dose [Prevention of myoclonus after etomidate using a priming dose]. Ann Fr Anesth Reanim. 2006 Oct;25(10):1041-5. French. doi: 10.1016/j.annfar.2006.07.079. Epub 2006 Sep 26. PMID: 17005362.
637	17047136	Hydromorphone	2006	Patel S, Roshan VR, Lee KC, Cheung RJ. A myoclonic reaction with low-dose hydromorphone. Ann Pharmacother. 2006 Nov;40(11):2068-70. doi: 10.1345/aph.1H243. Epub 2006 Oct 17. PMID: 17047136.
638	17201707	Lamotrigine	2006	Genton P, Gelisse P, Crespel A. Lack of efficacy and potential aggravation of myoclonus with lamotrigine in Unverricht-Lundborg disease. Epilepsia. 2006 Dec;47(12):2083-5. doi: 10.1111/j.1528-1167.2006.00829.x. PMID: 17201707.
639	17302087	Bupivacaine	2006	Plaza Moral AM, de Fernández Lopez Hierro C, Pons Casaovas M, Gomar Sancho C. Toxicidad materna por bupivacaína durante fetoscopia para el tratamiento del síndrome de transfusión fetofetal [Toxic maternal dose of bupivacaine during fetoscopic treatment of twin-to-twin transfusion syndrome]. Rev Esp Anestesiol Reanim. 2006 Dec;53(10):671-3. Spanish. PMID: 17302087.
640	17325906	Ceftazidime	2007	Martin MG. Encephalopathy with myoclonic jerks resulting from ceftazidime therapy: an under-recognized potential side-effect when treating febrile neutropenia. Leuk Lymphoma. 2007 Feb;48(2):413-4. doi: 10.1080/10428190601039755. PMID: 17325906.
641	17336778	Quinolone, beta-lactam, cephalosporin, aminoglycoside, tetracycline, acyclovir, SSRI, imipramine, MAO selective inhibitors, atypical antidepressants, lithium, benzodiazepine, zolpidem, zopiclone, buspirone, full agonist (morphine, fentanyl), partial agonist (buprenorphine), tramadol, dextropropoxyphene, levodopa, dopamine agonists, amantadine, entacapone, selegiline, typical antipsychotics, atypical antipsychotics, metoclopramide, domperidone, cholinesterase inhibitors, gabapentine, carbamazepine, valproic acid, phenobarbital, phenytoin, propofol, prednisolone, ketoprofen, buflomedil, furosemide, amiodarone, omeprazole	2006	Brefel-Courbon C, Gardette V, Ory F, Montastruc JL. Drug-induced myoclonus: a French pharmacovigilance database study. Neurophysiol Clin. 2006 Sep-Dec;36(5-6):333-6. doi: 10.1016/j.neucli.2006.12.003. Epub 2007 Jan 25. PMID: 17336778.
642	17415473	Valproate, melatonin	2006	Savina TA, Balashova OA, Shchipakina TG. Effect of chronic consumption of sodium valproate and melatonin on seizure activity in Krushinskii-Molodkina rats. Bull Exp Biol Med. 2006 Nov;142(5):601-4. doi: 10.1007/s10517-006-0429-0. PMID: 17415473.
643	17415801	Crack cocaine	2007	Kamath S, Bajaj N. Crack dancing in the United Kingdom: apropos a video case presentation. Mov Disord. 2007 Jun 15;22(8):1190-1. doi: 10.1002/mds.21430. PMID: 17415801.
644	17430825	Opioids	2007	Vella-Brincat J, Macleod AD. Adverse effects of opioids on the central nervous systems of palliative care patients. J Pain Palliat Care Pharmacother. 2007;21(1):15-25. PMID: 17430825.
645	17443899	Morphine	2007	Zhu-Ge ZB, Zhu YY, Wu DC, Jin CL, Chen Z. [Involvement of endogenous histamine in modulatory effect of morphine on seizure susceptibility in mice]. Zhejiang Da Xue Xue Bao Yi Xue Ban. 2007 Mar;36(2):130-3, 154. Chinese. doi: 10.3785/j.issn.1008-9292.2007.02.005. PMID: 17443899.
646	17471876	Etomidate	2007	Kuczkowski KM, Hastings BH. Severe myoclonus prior to electroconvulsive therapy following intravenous etomidate. AANA J. 2007 Apr;75(2):88. PMID: 17471876.
647	17516482	Ciprofloxacin	2007	Striano P, Zara F, Coppola A, Ciampa C, Pezzella M, Striano S. Epileptic myoclonus as ciprofloxacin-associated adverse effect. Mov Disord. 2007 Aug 15;22(11):1675-6. doi: 10.1002/mds.21456. PMID: 17516482.
648	17542734	Morphine	2007	da Cunha AF, Carter JE, Grafinger M, Montgomery H, Marks SL, Posner LP, Burns P. Intrathecal morphine overdose in a dog. J Am Vet Med Assoc. 2007 Jun 1;230(11):1665-8. doi: 10.2460/javma.230.11.1665. PMID: 17542734.
649	17564783	Tacrolimus	2007	Azuma T, Oishi M, Takei M, Sawada S. Tacrolimus-related nocturnal myoclonus of the lower limbs in elderly patients with

				rheumatoid arthritis. Mod Rheumatol. 2007;17(3):247-50. doi: 10.1007/s10165-007-0574-y. Epub 2007 Jun 20. PMID: 17564783.
650	17597235	NA, likely antipsychotics	2008	Tse W, Libow LS, Neufeld R, Lesser G, Frank J, Dolan S, Tarshish C, Gracies JM, Olanow CW, Koller WC, Hälbig TD. Prevalence of movement disorders in an elderly nursing home population. Arch Gerontol Geriatr. 2008 May-Jun;46(3):359-66. doi: 10.1016/j.archger.2007.05.008. Epub 2007 Jun 26. PMID: 17597235.
651	17602048	Bupivacaine, fentanyl	2007	Batra YK, Rajeev S, Lokesh VC, Rao KL. Spinal myoclonus associated with intrathecal bupivacaine and fentanyl in an infant. Can J Anaesth. 2007 Jul;54(7):587-8. doi: 10.1007/BF03022328. PMID: 17602048.
652	17618536	Levodopa	2007	Zesiewicz TA, Sullivan KL, Hauser RA. Levodopa-induced dyskinesia in Parkinson's disease: epidemiology, etiology, and treatment. Curr Neurol Neurosci Rep. 2007 Jul;7(4):302-10. doi: 10.1007/s11910-007-0046-y. PMID: 17618536.
653	17621754	Antipsychotic	2007	Kasantikul D, Kanchanatawan B. Antipsychotic-induced tardive movement disorders: a series of twelve cases. J Med Assoc Thai. 2007 Jan;90(1):188-94. PMID: 17621754.
654	17632233	Memantine	2007	Papageorgiou SG, Kontaxis T, Antelli A, Kalfakis N. Exacerbation of myoclonus by memantine in a patient with Alzheimer disease. J Clin Psychopharmacol. 2007 Aug;27(4):407-8. doi: 10.1097/01.jcp.0000264995.31771.05. PMID: 17632233.
655	17685135	Nitrous oxide	2007	Wu MS, Hsu YD, Lin JC, Chen SC, Lee JT. Spinal myoclonus in subacute combined degeneration caused by nitrous oxide intoxication. Acta Neurol Taiwan. 2007 Jun;16(2):102-5. PMID: 17685135.
656	17708573	Mercury	2007	Ragothaman M, Kulkarni G, Ashraf VV, Pal PK, Chickabasavaiah Y, Shankar SK, Govindappa SS, Satishchandra P, Muthane UB. Elemental mercury poisoning probably causes cortical myoclonus. Mov Disord. 2007 Oct 15;22(13):1964-8. doi: 10.1002/mds.21641. PMID: 17708573.
657	17721114	Interferon	2007	Brito MO, Doyle T. Movement and extrapyramidal disorders associated with interferon use in HIV/hepatitis C coinfection. AIDS. 2007 Sep 12;21(14):1987-9. doi: 10.1097/QAD.0b013e32829fb369. PMID: 17721114.
658	17900503	Atropine	2007	Moos DD. Central anticholinergic syndrome: a case report. J Perianesth Nurs. 2007 Oct;22(5):309-21. doi: 10.1016/j.jopan.2007.05.006. PMID: 17900503.
659	17914519	Etomidate	2007	Akcaboy ZN, Akcaboy EY, Altinoren B, Karabulut E, Gogus N. Adding remifentanil to propofol and etomidate in cardioversion anesthesia. Saudi Med J. 2007 Oct;28(10):1550-4. PMID: 17914519.
660	17922247	Picrotoxin	2007	Yakimovskii AF, Varshavskaya VM. Magnesium ions prevent the development of hyperkinesia evoked by administration of picrotoxin into the rat neostriatum. Neurosci Behav Physiol. 2007 Oct;37(8):821-6. doi: 10.1007/s11055-007-0087-3. PMID: 17922247.
661	17922777	Topiramate	2007	Kutluay E, Pakoz B, Beydoun A. Reversible facial myoclonus with topiramate therapy for epilepsy. Epilepsia. 2007 Oct;48(10):2001-2. doi: 10.1111/j.1528-1167.2007.01165_3.x. PMID: 17922777.
662	17959575	Dobutamine	2007	Boord A, Benson B. Myoclonus associated with continuous dobutamine infusion in a patient with end-stage renal disease. Am J Health Syst Pharm. 2007 Nov 1;64(21):2241-3. doi: 10.2146/ajhp060326. PMID: 17959575.
663	17959958	Etomidate	2007	Hüter L, Schreiber T, Gugel M, Schwarzkopf K. Low-dose intravenous midazolam reduces etomidate-induced myoclonus: a prospective, randomized study in patients undergoing elective cardioversion. Anesth Analg. 2007 Nov;105(5):1298-302, table of contents. doi: 10.1213/01.ane.0000287248.25610.c0. PMID: 17959958.
664	18004234	Olanzapine, clomipramine	2007	Verre M, Bossio F, Mammone A, Piccirillo M, Tancioni F, Tortorella V, Varano M. Serotonin syndrome caused by olanzapine and clomipramine. Minerva Anestesiol. 2008 Jan-Feb;74(1-2):41-5. Epub 2007 Nov 16. PMID: 18004234.
665	18021110	Tiagabine	2007	Forbes RA, Kalra H, Hackett LP, Daly FF. Deliberate self-poisoning with tiagabine: an unusual toxidrome. Emerg Med Australas. 2007 Dec;19(6):556-8. doi: 10.1111/j.1742-6723.2007.00973.x. PMID: 18021110.
666	18035006	Etomidate	2007	Uzun S, Gözaçan A, Canbay O, Ozgen S. Remifentanil and etomidate for laryngeal mask airway insertion. J Int Med Res. 2007 Nov-Dec;35(6):878-85. doi: 10.1177/147323000703500616. PMID: 18035006.
667	18061410	Meropenem	2008	Baraboutis IG, Marangos MN, Skoutelis A, Bassaris H. Meropenem-aggravated seizure activity in progressive myoclonus epilepsy. Int J Antimicrob Agents. 2008 Feb;31(2):177-9. doi: 10.1016/j.ijantimicag.2007.09.011. Epub 2007 Dec 3. PMID: 18061410.
668	18063208	Propofol	2007	Lenkovsky F, Robertson BD, Iyer C, Ross L, Ahmed SA, Herazo L, Markin V, Joshi GP. Metoclopramide does not influence the frequency of propofol-induced spontaneous movements. J Clin Anesth. 2007 Nov;19(7):530-3. doi: 10.1016/j.jclinane.2007.05.004. PMID: 18063208.
669	18175786	Cefepime	2008	Sonck J, Laureys G, Verbeelen D. The neurotoxicity and safety of treatment with cefepime in patients with renal failure. Nephrol Dial Transplant. 2008 Mar;23(3):966-70. doi: 10.1093/ndt/gfm713. Epub 2008 Jan 5. PMID: 18175786.
670	18202466	Acyclovir	2004	Skhiri H, Achour A, Skhiri S, Frih A, Bouraoui S, Dhia NB, Elmay M. Neuropsychiatric manifestations in a patient undergoing hemodialysis caused by treatment with oral acyclovir. Saudi J Kidney Dis Transpl. 2004 Jan-Mar;15(1):50-2. PMID: 18202466.
671	18213744	Citalopram	2007	Turedi S, Eraydin I, Gunduz A, Kalkan A, Hos U. First time, low dose citalopram use-related serotonin syndrome. Neurotoxicology. 2007 Nov;28(6):1272-4. doi: 10.1016/j.neuro.2007.05.004. PMID: 18213744.
672	18222628	Fentanyl	2008	Okon TR, George ML. Fentanyl-induced neurotoxicity and paradoxic pain. J Pain Symptom Manage. 2008 Mar;35(3):327-33. doi: 10.1016/j.jpainsymman.2007.04.023. Epub 2008 Jan 28. PMID: 18222628.
673	18226283	Spinal anaesthesia	2008	Alfa JA, Bamgbade OA. Acute myoclonus following spinal anaesthesia. Eur J Anaesthesiol. 2008 Mar;25(3):256-7. doi: 10.1017/S0265021507002578. PMID: 18226283.
674	18230263	Etomidate	2008	Hwang JY, Kim JH, Oh AY, Do SH, Jeon YT, Han SH. A comparison of midazolam with remifentanil for the prevention of myoclonic movements following etomidate injection. J Int Med Res. 2008 Jan-Feb;36(1):17-22. doi: 10.1177/147323000803600103. PMID: 18230263.

675	18303133	Memantine, trimethoprim	2008	Moellentin D, Picone C, Leadbetter E. Memantine-induced myoclonus and delirium exacerbated by trimethoprim. Ann Pharmacother. 2008 Mar;42(3):443-7. doi: 10.1345/aph.1K619. Epub 2008 Feb 26. PMID: 18303133.
676	18344744	Lamotrigine	2008	Fernández Corcuera P, Pomarol E, Amann B, McKenna P. Myoclonus provoked by lamotrigine in a bipolar patient. J Clin Psychopharmacol. 2008 Apr;28(2):248-9. doi: 10.1097/JCP.0b013e318167465f. PMID: 18344744.
677	18376118	Flecainide	2008	Ting SM, Lee D, Maclean D, Sheerin NS. Paranoid psychosis and myoclonus: flecainide toxicity in renal failure. Cardiology. 2008;111(2):83-6. doi: 10.1159/000119694. Epub 2008 Mar 31. PMID: 18376118.
678	18389190	Bupivacaine	2008	Lin CS, Wei-Hung C, Lee YW. Transient spinal myoclonus after spinal anaesthesia with bupivacaine in the perioperation period. Anaesthesist. 2008 May;57(5):518. doi: 10.1007/s00101-008-1362-6. PMID: 18389190.
679	18443635	Opioid	2008	Benyamin R, Trescot AM, Datta S, Buenaventura R, Adlaka R, Sehgal N, Glaser SE, Vallejo R. Opioid complications and side effects. Pain Physician. 2008 Mar;11(2 Suppl):S105-20. PMID: 18443635.
680	18486797	Intravenous sedation	2008	Schneider R, Reebye U, Choi C, Kalman D. Seizure-like activity and prolonged central nervous system side effects after intravenous sedation. J Oral Maxillofac Surg. 2008 Jun;66(6):1277-82. doi: 10.1016/j.joms.2007.04.034. PMID: 18486797.
681	18492617	Pregabalin	2008	Hellwig S, Amtage F. Pregabalin-induced cortical negative myoclonus in a patient with neuropathic pain. Epilepsy Behav. 2008 Aug;13(2):418-20. doi: 10.1016/j.yebeh.2008.04.006. Epub 2008 May 19. PMID: 18492617.
682	18579443	Pregabalin	2008	Modur PN, Milteer WE. Adjunctive pregabalin therapy in mentally retarded, developmentally delayed patients with epilepsy. Epilepsy Behav. 2008 Oct;13(3):554-6. doi: 10.1016/j.yebeh.2008.05.003. Epub 2008 Jun 24. PMID: 18579443.
683	18581470	Oxatomide	2008	Irioka T, Machida A, Yokota T, Mizusawa H. Antihistamine-associated myoclonus: A case report. Mov Disord. 2008 Aug 15;23(11):1615-6. doi: 10.1002/mds.22076. PMID: 18581470.
684	18624020	Citalopram, linezolid	2008	Lorenz RA, Vandenberg AM, Canepa EA. Serotonergic antidepressants and linezolid: a retrospective chart review and presentation of cases. Int J Psychiatry Med. 2008;38(1):81-90. doi: 10.2190/PM.38.1.h. PMID: 18624020.
685	18668386	Bromovalerylurea	2008	Lin JN, Lin HL, Huang CK, Lai CH, Chung HC, Liang SH, Lin HH. Myoclonic jerks due to acute bromovalerylurea intoxication. Clin Toxicol (Phila). 2008 Nov;46(9):861-3. doi: 10.1080/15563650802020361. PMID: 18668386.
686	18686651	Amantadine, pramipexole	2008	Hong CT, Sun Y, Lu CJ. Fatal intoxication using amantadine and pramipexole in a uremic patient. Acta Neurol Taiwan. 2008 Jun;17(2):109-11. PMID: 18686651.
687	18702113	Gabapentin	2008	Ege F, Koçak Y, Titiz AP, Oztürk SM, Oztürk S, Ozbakir S. Gabapentin-Induced myoclonus: case report. Mov Disord. 2008 Oct 15;23(13):1947-8. doi: 10.1002/mds.21911. PMID: 18702113.
688	18715174	Methadone	2008	Ito S, Liao S. Myoclonus associated with high-dose parenteral methadone. J Palliat Med. 2008 Jul;11(6):838-41. doi: 10.1089/jpm.2008.0040. PMID: 18715174.
689	18823813	Amantadine	2009	Nishikawa N, Nagai M, Moritoyo T, Yabe H, Nomoto M. Plasma amantadine concentrations in patients with Parkinson's disease. Parkinsonism Relat Disord. 2009 Jun;15(5):351-3. doi: 10.1016/j.parkreldis.2008.08.005. Epub 2008 Sep 27. PMID: 18823813.
690	18840374	Gabapentin	2008	Pierce DA, Holt SR, Reeves-Daniel A. A probable case of gabapentin-related reversible hearing loss in a patient with acute renal failure. Clin Ther. 2008 Sep;30(9):1681-4. doi: 10.1016/j.clinthera.2008.09.004. PMID: 18840374.
691	18972571	Ondansetron, propofol	2009	Kumar N, Hu WT. Extrapyramidal reaction to ondansetron and propofol. Mov Disord. 2009 Jan 30;24(2):312-3. doi: 10.1002/mds.22367. PMID: 18972571.
692	19008618	Quetiapine	2008	Aggarwal A, Jiloha RC. Quetiapine induced myoclonus. Indian J Med Sci. 2008 Oct;62(10):422-3. PMID: 19008618.
693	19033476	Cephalosporin	2008	Grill MF, Maganti R. Cephalosporin-induced neurotoxicity: clinical manifestations, potential pathogenic mechanisms, and the role of electroencephalographic monitoring. Ann Pharmacother. 2008 Dec;42(12):1843-50. doi: 10.1345/aph.1L307. Epub 2008 Nov 25. PMID: 19033476.
694	19050415	Topiramate, fluvoxamine	2008	Oulis P, Potagas C, Masdrakis VG, Thomopoulos Y, Kouzoupis AV, Soldatos CR. Reversible tremor and myoclonus associated with topiramate-fluvoxamine coadministration. Clin Neuropharmacol. 2008 Nov-Dec;31(6):366-7. doi: 10.1097/WNF.0b013e31815ce4c2. PMID: 19050415.
695	19096603	Gabapentin	2008	Cho KT, Hong SK. Myoclonus induced by the use of gabapentin. J Korean Neurosurg Soc. 2008 May;43(5):237-8. doi: 10.3340/jkns.2008.43.5.237. Epub 2008 May 20. PMID: 19096603; PMCID: PMC2588223.
696	19099135	Venlafaxine	2008	Dutra LA, Pedroso JL, Felix EP, Barsottini OG. Venlafaxine induced-myoclonus in a patient with mixed dementia. Arq Neuropsiquiatr. 2008 Dec;66(4):894-5. doi: 10.1590/s0004-282x2008000600025. PMID: 19099135.
697	19100933	Etomidate	2008	Choi JM, Choi IC, Jeong YB, Kim TH, Hahm KD. Pretreatment of rocuronium reduces the frequency and severity of etomidate-induced myoclonus. J Clin Anesth. 2008 Dec;20(8):601-4. doi: 10.1016/j.jclinane.2008.06.010. PMID: 19100933.
698	19201620	Levetiracetam	2009	Vulliemoz S, Iwanowski P, Landis T, Jallon P. Levetiracetam accumulation in renal failure causing myoclonic encephalopathy with triphasic waves. Seizure. 2009 Jun;18(5):376-8. doi: 10.1016/j.seizure.2009.01.006. Epub 2009 Feb 7. PMID: 19201620.
699	19227708	Morphine	2008	Shelton BW, Deynes-Romero J, Tofani-Montalvo M, Ramírez-Rivera J, Jaumne-Anselmi F. Methadone: an effective alternative to morphine for pain relief in cancer patients. Bol Asoc Med P R. 2008 Jul-Sep;100(3):7-10. PMID: 19227708.
700	19332219	Amlodipine	2009	Wallace EL, Lingle K, Pierce D, Satko S. Amlodipine-induced myoclonus. Am J Med. 2009 Apr;122(4):e7. doi: 10.1016/j.amjmed.2008.10.036. PMID: 19332219.
701	19345234	Pentylenetetrazole	2009	Vlainić J, Pericić D. Effects of acute and repeated zolpidem treatment on pentylenetetrazole-induced seizure threshold and on locomotor activity: comparison with diazepam. Neuropharmacology. 2009 Jun;56(8):1124-30. doi: 10.1016/j.neuropharm.2009.03.010. Epub 2009 Apr 1. PMID: 19345234.

702	19384813	Gamma-butyrolactone	2009	Supady A, Schwab T, Busch HJ. "Liquid Ecstasy": Gamma-Butyrolacton-Entzugsdelir mit Rhabdomyolyse und dialysepflichtiger Niereninsuffizienz ["Liquid ecstasy": gamma-butyrolactone withdrawal delirium with rhabdomyolysis and dialysis dependent renal failure]. Dtsch Med Wochenschr. 2009 Apr;134(18):935-7. German. doi: 10.1055/s-0029-1220252. Epub 2009 Apr 21. PMID: 19384813.
703	19396333	Cefepime	2009	Martín Herrera C, Navarro M. Encefalopatía por cefepima en pacientes con insuficiencia renal [Cefepime-induced encephalopathy in patients with renal failure]. Nefrologia. 2009;29(2):181. Spanish. doi: 10.3265/Nefrologia.2009.29.2.4911.en.full. PMID: 19396333.
704	19432690	Metformin	2009	Jung EY, Cho HS, Seo JW, Kim DW, Kim HJ, Chang SH, Park DJ. Metformin-induced encephalopathy without lactic acidosis in a patient with contraindication for metformin. Hemodial Int. 2009 Apr;13(2):172-5. doi: 10.1111/j.1542-4758.2009.00358.x. PMID: 19432690.
705	19435583	Duloxetine, paroxetine	2009	Melani F, Rosati E, Chiocchetti B, Muscas GC. Antidepressant-associated myoclonic status in a patient with symptomatic generalized epilepsy: does risk occur with therapeutic doses? Epilepsy Behav. 2009 Apr;14(4):681-3. doi: 10.1016/j.yebeh.2009.01.018. Epub 2009 Jan 31. PMID: 19435583.
706	19448237	Tranexamic acid	2009	Mohseni K, Jafari A, Nobahar MR, Arami A. Polymyoclonus seizure resulting from accidental injection of tranexamic acid in spinal anesthesia. Anesth Analg. 2009 Jun;108(6):1984-6. doi: 10.1213/ane.0b013e3181a04d69. PMID: 19448237.
707	19489007	Propofol	2009	Lu CW, Lin TY, Chiang HS, Wang SJ. Facilitation of glutamate release from rat cerebral cortex nerve terminal by subanesthetic concentration propofol. Synapse. 2009 Sep;63(9):773-81. doi: 10.1002/syn.20656. PMID: 19489007.
708	19493601	Pregabalin	2009	Yoo L, Matalon D, Hoffman RS, Goldfarb DS. Treatment of pregabalin toxicity by hemodialysis in a patient with kidney failure. Am J Kidney Dis. 2009 Dec;54(6):1127-30. doi: 10.1053/j.ajkd.2009.04.014. Epub 2009 Jun 3. PMID: 19493601.
709	19499876	Prilocaine	2009	Fores Novales B, Aguilera Celorrio L. Spinal myoclonus following intrathecal anaesthesia with prilocaine. Anaesth Intensive Care. 2009 May;37(3):498-9. PMID: 19499876.
710	19513284	Propofol	2007	Jeon HW, Kang JH, Kim HS, Jo HY, Kim SH. A case of propofol-induced delayed-onset refractory myoclonic seizures. J Clin Neurol. 2007 Sep;3(3):154-7. doi: 10.3988/jcn.2007.3.3.154. Epub 2007 Sep 20. PMID: 19513284; PMCID: PMC2686843.
711	19525493	Cobalamin	2010	Ozdemir O, Baytan B, Gunes AM, Okan M. Involuntary movements during vitamin B12 treatment. J Child Neurol. 2010 Feb;25(2):227-30. doi: 10.1177/0883073809333528. Epub 2009 Jun 12. PMID: 19525493.
712	19526195	Haloperidol	2009	Domínguez C, Benito-León J, Bermejo-Pareja F. Multifocal myoclonus induced by haloperidol. Neurol Sci. 2009 Oct;30(5):385-6. doi: 10.1007/s10072-009-0104-0. Epub 2009 Jun 13. PMID: 19526195.
713	19590258	Cyclobenzaprine	2009	Reuss R, Reuter I, Jauss M, Fischer F, Muller SC, Stolz E. Torticollis under cyclobenzaprine. Pharmacology. 2009;84(2):91-2. doi: 10.1159/000227773. Epub 2009 Jul 8. PMID: 19590258.
714	19618843	Gabapentin	2009	Koide Y, Ikeda H, Inoue Y. [Development or worsening of myoclonus associated with gabapentin therapy]. Rinsho Shinkeigaku. 2009 Jun;49(6):342-7. Japanese. doi: 10.5692/clinicalneurol.49.342. PMID: 19618843.
715	19640240	Topiramate	2009	Meral C, Aydinoz S. Simple dosage inaccuracy might be the cause of serious side effects of topiramate. Clin Toxicol (Phila). 2009 Aug;47(7):691. doi: 10.1080/15563650903158888. PMID: 19640240.
716	19640241	Avermectin	2009	Sung YF, Huang CT, Fan CK, Lin CH, Lin SP. Avermectin intoxication with coma, myoclonus, and polyneuropathy. Clin Toxicol (Phila). 2009 Aug;47(7):686-8. doi: 10.1080/15563650903070901. PMID: 19640241.
717	19642217	Pregabalin, gabapentin	2009	Healy DG, Ingle GT, Brown P. Pregabalin- and gabapentin-associated myoclonus in a patient with chronic renal failure. Mov Disord. 2009 Oct 15;24(13):2028-9. doi: 10.1002/mds.22286. PMID: 19642217.
718	19721703	Valproic acid	2009	Gardner TM, Aziz R, Muralee S, Tampi RR. Valproic Acid-induced myoclonus in a demented patient: a case report. Case Rep Med. 2009;2009:392091. doi: 10.1155/2009/392091. Epub 2009 Aug 9. PMID: 19721703; PMCID: PMC2728607.
719	19744831	Flurothyl	2009	Papandrea D, Kukol WS, Anderson TM, Herron BJ, Ferland RJ. Analysis of flurothyl-induced myoclonus in inbred strains of mice. Epilepsy Res. 2009 Dec;87(2-3):130-6. doi: 10.1016/j.eplepsyres.2009.08.003. Epub 2009 Sep 9. PMID: 19744831; PMCID: PMC2788057.
720	19770559	Olanzapine	2009	Majumder S, Mandal SK, Guha G, Bandyopadhyay D, Chowdhury SR. A single fatal dose of olanzapine. Neurol India. 2009 Jul-Aug;57(4):497. doi: 10.4103/0028-3886.55569. PMID: 19770559.
721	19775046	Propofol, etomidate	2009	Tan HL, Lee CY. Comparison between the effects of propofol and etomidate on motor and electroencephalogram seizure duration during electroconvulsive therapy. Anaesth Intensive Care. 2009 Sep;37(5):807-14. doi: 10.1177/0310057X0903700509. PMID: 19775046.
722	19776320	Clozapine	2009	Butler T. Clozapine-induced negative myoclonus is not cataplexy. J Neuropsychiatry Clin Neurosci. 2009 Summer;21(3):345-6. doi: 10.1176/jnp.2009.21.3.345. PMID: 19776320.
723	19824991	Clozapine	2010	Cormac I, Brown A, Creasey S, Ferriter M, Huckstep B. A retrospective evaluation of the impact of total smoking cessation on psychiatric inpatients taking clozapine. Acta Psychiatr Scand. 2010 May;121(5):393-7. doi: 10.1111/j.1600-0447.2009.01482.x. Epub 2009 Oct 13. PMID: 19824991.
724	19825064	Propofol	2009	Tam MK, Irwin MG, Tse ML, Lui YW, Law KI, Ng PW. Prolonged myoclonus after a single bolus dose of propofol. Anaesthesia. 2009 Nov;64(11):1254-7. doi: 10.1111/j.1365-2044.2009.06035.x. Erratum in: Anaesthesia. 2010 Feb;65(2):222. PMID: 19825064.
725	19830168	Central neuraxial anaesthesia	2009	Bamgbade OA, Alfa JA, Khalaf WM, Zuokumor AP. Central neuraxial anaesthesia presenting with spinal myoclonus in the perioperative period: a case series. J Med Case Rep. 2009 Jun 23;3:7293. doi: 10.4076/1752-1947-3-7293. PMID: 19830168; PMCID: PMC2726533.
726	19833162	Clozapine	2010	Praharaj SK, Venkatesh BG, Sarkhel S, Zia-ul-Haq M, Sinha VK. Clozapine-induced myoclonus: a case study and brief review. Prog Neuropsychopharmacol Biol Psychiatry. 2010 Feb 1;34(1):242-3. doi: 10.1016/j.pnpbp.2009.10.006. Epub 2009 Oct 13. PMID: 19833162.

727	19833162	Clozapine	2010	Praharaj SK, Venkatesh BG, Sarkhel S, Zia-ul-Haq M, Sinha VK. Clozapine-induced myoclonus: a case study and brief review. Prog Neuropsychopharmacol Biol Psychiatry. 2010 Feb 1;34(1):242-3. doi: 10.1016/j.pnpbp.2009.10.006. Epub 2009 Oct 13. PMID: 19833162.
728	19837813	Local anaesthetics	2009	Pasquier P, Ausset S, Pelee De Saint Maurice G, Vest P, Mazoit JX, Auroy Y. Convulsions associated with a low plasma level of local anaesthetics. Br J Anaesth. 2009 Nov;103(5):776. doi: 10.1093/bja/aep285. PMID: 19837813.
729	19954712	Ropivacaine	2009	Huschak G, Rüffert H, Wehner M, Taubert MH, Preiss R, Meinecke CD, Kaisers UX, Regenthal R. Pharmacokinetics and clinical toxicity of prilocaine and ropivacaine following combined drug administration in brachial plexus anesthesia. Int J Clin Pharmacol Ther. 2009 Dec;47(12):733-43. PMID: 19954712.
730	20013432	Linezolid	2009	Sahiner V, Erden Aki SO. Linezolid kullanimi ile ilişkili serotonin sendromu: olgu sunumu [Serotonin syndrome associated with linezolid use: a case report]. Turk Psikiyatri Derg. 2009 Winter;20(4):398-402. Turkish. PMID: 20013432.
731	20018300	Etomidate	2010	Mizrak A, Koruk S, Bilgi M, Kocamer B, Erkutlu I, Ganidagli S, Oner U. Pretreatment with dexmedetomidine or thiopental decreases myoclonus after etomidate: a randomized, double-blind controlled trial. J Surg Res. 2010 Mar;159(1):e11-6. doi: 10.1016/j.jss.2009.07.031. Epub 2009 Aug 19. PMID: 20018300.
732	20059367	Isotretinoin	2010	Wong A, Williams M, Gibb W. Isotretinoin-induced encephalopathy. J Dermatolog Treat. 2010 Nov;21(6):361-2. doi: 10.3109/09546630903341978. PMID: 20059367.
733	20096021	Caffeine	2010	Rudolph T, Knudsen K. A case of fatal caffeine poisoning. Acta Anaesthesiol Scand. 2010 Apr;54(4):521-3. doi: 10.1111/j.1399-6576.2009.02201.x. Epub 2010 Jan 21. PMID: 20096021.
734	20108006	Etomidate	2010	Gultop F, Akkaya T, Bedirli N, Gumus H. Lidocaine pretreatment reduces the frequency and severity of myoclonus induced by etomidate. J Anesth. 2010 Apr;24(2):300-2. doi: 10.1007/s00540-010-0869-6. Epub 2010 Jan 28. PMID: 20108006.
735	20163444	Etomidate	2010	Pastor J, Wix R, Meilán ML, Martínez-Chacón JL, de Dios E, Domínguez-Gadea L, Herrera-Peco I, Sola RG. Etomidate accurately localizes the epileptic area in patients with temporal lobe epilepsy. Epilepsia. 2010 Apr;51(4):602-9. doi: 10.1111/j.1528-1167.2009.02500.x. Epub 2010 Feb 12. PMID: 20163444.
736	20332743	Bupivacaine	2010	Zamidei L, Bandini M, Michelagnoli G, Campostrini R, Consales G. Propriospinal myoclonus following intrathecal bupivacaine in hip surgery: a case report. Minerva Anestesiol. 2010 Apr;76(4):290-3. Epub 2009 Dec 23. PMID: 20332743.
737	20434435	Memantine	2010	Tai KK, Truong DD. Memantine exacerbates myoclonic jerks in a rat model of posthypoxic myoclonus. Brain Res. 2010 Jul 9;1343:194-8. doi: 10.1016/j.brainres.2010.04.058. Epub 2010 Apr 29. PMID: 20434435.
738	20481173	Morphine, hydromorphone	2010	McCann S, Yaksh TL, von Gunten CF. Correlation between myoclonus and the 3-glucuronide metabolites in patients treated with morphine or hydromorphone: a pilot study. J Opioid Manag. 2010 Mar-Apr;6(2):87-94. doi: 10.5055/jom.2010.0008. PMID: 20481173.
739	20483715	Levetiracetam	2010	Caraballo RH, Cersósimo R, De los Santos C. Levetiracetam-induced seizure aggravation associated with continuous spikes and waves during slow sleep in children with refractory epilepsies. Epileptic Disord. 2010 Jun;12(2):146-50. doi: 10.1684/epd.2010.0305. Epub 2010 May 20. PMID: 20483715.
740	20498288	Etomidate	2011	Kaneda K, Yamashita S, Woo S, Han TH. Population pharmacokinetics and pharmacodynamics of brief etomidate infusion in healthy volunteers. J Clin Pharmacol. 2011 Apr;51(4):482-91. doi: 10.1177/0091270010369242. Epub 2010 May 24. PMID: 20498288.
741	20560386	Epidural anesthesia	2010	Hisano Y, Nakamura K, Kitamura R, Ogino Y, Hirata S, Fukami N, Kanoe H, Fujiwara Y. [Myoclonus following spinal and epidural anesthesia--a case report]. Masui. 2010 Jun;59(6):770-2. Japanese. PMID: 20560386.
742	20578939	Clozapine, clomipramine	2010	Horga G, Horga A, Baeza I, Castro-Fornieles J, Lázaro L, Pons A. Drug-induced speech dysfluency and myoclonus preceding generalized tonic-clonic seizures in an adolescent male with schizophrenia. J Child Adolesc Psychopharmacol. 2010 Jun;20(3):233-4. doi: 10.1089/cap.2009.0010. PMID: 20578939.
743	20592323	Topiramate	2010	Miller AD, Prost VM, Bookstaver PB, Gaines KJ. Topiramate-induced myoclonus and psychosis during migraine prophylaxis. Am J Health Syst Pharm. 2010 Jul 15;67(14):1178-80. doi: 10.2146/ajhp090185. PMID: 20592323.
744	20592570	Carisoprodol	2010	Eleid MF, Krahn LE, Agrwal N, Goodman BP. Carisoprodol withdrawal after internet purchase. Neurologist. 2010 Jul;16(4):262-4. doi: 10.1097/NRL.0b013e3181aa917e. PMID: 20592570.
745	20721928	Ethanol	2010	Brust JC. Substance abuse and movement disorders. Mov Disord. 2010 Oct 15;25(13):2010-20. doi: 10.1002/mds.22599. PMID: 20721928.
746	20740488	Amantadine	2010	Gupta A, Lang AE. Drug-induced cranial myoclonus. Mov Disord. 2010 Oct 15;25(13):2264-5. doi: 10.1002/mds.23140. PMID: 20740488.
747	20817933	Ciprofloxacin	2010	Jayathissa S, Woolley M, Ganasegaram M, Holden J, Cu E. Myoclonus and delirium associated with ciprofloxacin. Age Ageing. 2010 Nov;39(6):762. doi: 10.1093/ageing/afq107. Epub 2010 Sep 4. PMID: 20817933.
748	20870687	Opioid	2011	Stone P, Minton O. European Palliative Care Research collaborative pain guidelines. Central side-effects management: what is the evidence to support best practice in the management of sedation, cognitive impairment and myoclonus? Palliat Med. 2011 Jul;25(5):431-41. doi: 10.1177/0269216310380763. Epub 2010 Sep 24. PMID: 20870687.
749	20884828	Citalopram	2010	Zand L, Hoffman SJ, Nyman MA. 74-year-old woman with new-onset myoclonus. Mayo Clin Proc. 2010 Oct;85(10):955-8. doi: 10.4065/mcp.2009.0572. PMID: 20884828; PMCID: PMC2947969.
750	20932710	Fentanyl	2010	Essandoh S, Sakae M, Miller J, Glare PA. A cautionary tale from critical care: resolution of myoclonus after fentanyl rotation to hydromorphone. J Pain Symptom Manage. 2010 Nov;40(5):e4-6. doi: 10.1016/j.jpainsymman.2010.08.005. Epub 2010 Oct 8. PMID: 20932710.
751	21106426	Galantamine	2011	Hernández-Fernández F, Pardal-Fernández JM, García-Martínez E, Segura T. Respiratory myoclonus, a side effect of galantamine. Farm Hosp. 2011 Mar-Apr;35(2):97-9. English, Spanish. doi: 10.1016/j.farma.2010.06.001. Epub 2010 Nov 23. PMID: 21106426.

752	21167107	Clozapine	2011	Liang CS, Hsieh TH. Myoclonus as an indicator of infection in patients with schizophrenia treated with clozapine. J Psychiatry Neurosci. 2011 Jan;36(1):E1-2. doi: 10.1503/jpn.100133. PMID: 21167107; PMCID: PMC3004978.
753	21228393	Dextromethorphan	2011	Tanaka A, Nagamatsu T, Yamaguchi M, Nomura A, Nagura F, Maeda K, Tomino T, Watanabe T, Shimizu H, Fujita Y, Ito Y. Myoclonus after dextromethorphan administration in peritoneal dialysis. Ann Pharmacother. 2011 Jan;45(1):e1. doi: 10.1345/aph.1P301. Epub 2010 Dec 14. PMID: 21228393.
754	21233984	Monoamine oxidase inhibitors	1990	Remick RA, Froese C. Monoamine oxidase inhibitors: clinical review. Can Fam Physician. 1990 Jun;36:1151-5. PMID: 21233984; PMCID: PMC2280482.
755	21367668	Lacosamide, carbamazepine	2011	Belcastro V, Arnaboldi M, Taborelli A, Prontera P. Induction of epileptic negative myoclonus by addition of lacosamide to carbamazepine. Epilepsy Behav. 2011 Mar;20(3):589-90. doi: 10.1016/j.yebeh.2011.01.022. Epub 2011 Mar 1. PMID: 21367668.
756	21373307	Fluoxetine	2010	Arora B, Kannikeswaran N. The serotonin syndrome-the need for physician's awareness. Int J Emerg Med. 2010 Aug 20;3(4):373-7. doi: 10.1007/s12245-010-0195-7. PMID: 21373307; PMCID: PMC3047867.
757	21399985	Ifosfamide	2011	Savica R, Rabinstein AA, Josephs KA. Ifosfamide associated myoclonus-encephalopathy syndrome. J Neurol. 2011 Sep;258(9):1729-31. doi: 10.1007/s00415-011-5990-4. Epub 2011 Mar 12. PMID: 21399985.
758	21425106	Gabapentin	2011	Prieto-Pérez L, Montastruc J, García-Ruiz PJ. Mioclonías secundarias a gabapentina en un paciente con insuficiencia renal crónica [Myoclonias secondary to gabapentin in a patient with chronic renal failure]. Rev Neurol. 2011 Apr 16;52(8):512. Spanish. PMID: 21425106.
759	21431624	Methylergometrine	2011	Ishibashi T, Ishibashi S, Uchida T, Nakazawa K, Makita K. Reversible cerebral vasoconstriction syndrome with limb myoclonus following intravenous administration of methylergometrine. J Anesth. 2011 Jun;25(3):405-8. doi: 10.1007/s00540-011-1122-7. Epub 2011 Mar 23. PMID: 21431624.
760	21491099	Citalopram	2011	Talarico G, Tosto G, Pietracupa S, Piacentini E, Canevelli M, Lenzi GL, Bruno G. Serotonin toxicity: a short review of the literature and two case reports involving citalopram. Neurol Sci. 2011 Jun;32(3):507-9. doi: 10.1007/s10072-011-0546-z. Epub 2011 Apr 14. PMID: 21491099.
761	21532372	Escitalopram	2011	Tremolizzo L, Fermi S, Fusco ML, Susani E, Frigo M, Piolti R, Ferrarese C, Appollonio I. Generalized action myoclonus associated with escitalopram in a patient with mixed dementia. J Clin Psychopharmacol. 2011 Jun;31(3):394-5. doi: 10.1097/JCP.0b013e318218f4d5. PMID: 21532372.
762	21585220	Quinolones	2011	Tomé AM, Filipe A. Quinolones: review of psychiatric and neurological adverse reactions. Drug Saf. 2011 Jun 1;34(6):465-88. doi: 10.2165/11587280-000000000-00000. PMID: 21585220.
763	21632605	Etomidate	2011	Nyman Y, von Hofsten K, Ritzmo C, Eksborg S, Lönnqvist PA. Effect of a small priming dose on myoclonic movements after intravenous anaesthesia induction with Etomidate-Lipuro in children. Br J Anaesth. 2011 Aug;107(2):225-8. doi: 10.1093/bja/aer129. Epub 2011 Jun 1. PMID: 21632605.
764	21655019	Etomidate	2011	Saricaoglu F, Uzun S, Arun O, Arun F, Aypar U. A clinical comparison of etomidate-lipuro, propofol and admixture at induction. Saudi J Anaesth. 2011 Jan;5(1):62-6. doi: 10.4103/1658-354X.76509. PMID: 21655019; PMCID: PMC3101756.
765	21655027	Tranexamic acid	2011	Kaabachi O, Eddhif M, Rais K, Zaabar MA. Inadvertent intrathecal injection of tranexamic acid. Saudi J Anaesth. 2011 Jan;5(1):90-2. doi: 10.4103/1658-354X.76504. PMID: 21655027; PMCID: PMC3101765.
766	21823925	Hydromorphone	2011	Paramanandam G, Prommer E, Schwenke DC. Adverse effects in hospice patients with chronic kidney disease receiving hydromorphone. J Palliat Med. 2011 Sep;14(9):1029-33. doi: 10.1089/jpm.2011.0103. Epub 2011 Aug 8. PMID: 21823925.
767	21824061	Cathinones	2011	Spiller HA, Ryan ML, Weston RG, Jansen J. Clinical experience with and analytical confirmation of "bath salts" and "legal highs" (synthetic cathinones) in the United States. Clin Toxicol (Phila). 2011 Jul;49(6):499-505. doi: 10.3109/15563650.2011.590812. PMID: 21824061.
768	21874144	Metronidazole	2009	Foreid H, Coelho M, Ferreira J, Ferro JM. Reversible iatrogenic subacute cerebellar syndrome, myoclonus and MRI findings following metronidazole administration. BMJ Case Rep. 2009;2009:bcr05.2009.1841. doi: 10.1136/bcr.05.2009.1841. Epub 2009 Aug 28. PMID: 21874144; PMCID: PMC3027318.
769	21880885	Diltiazem	2011	Swanoski MT, Chen JS, Monson MH. Myoclonus associated with long-term use of diltiazem. Am J Health Syst Pharm. 2011 Sep 15;68(18):1707-10. doi: 10.2146/ajhp100704. PMID: 21880885.
770	21912040	Cefepime	2011	Yadla M, Kishore CK, Sriramnaveen P, Reddy YS, Sainaresh VV, Bhuma V, Sivakumar V. Neurotoxicity due to cefepime in patients on maintenance hemodialysis. Saudi J Kidney Dis Transpl. 2011 Sep;22(5):1026-7. PMID: 21912040.
771	21920212	Bupivacaine	2011	Abrão J, Bianco Mde P, Roma W, Krippa JE, Hallak JE. Spinal myoclonus after subarachnoid anesthesia with bupivacaine. Rev Bras Anestesiol. 2011 Sep-Oct;61(5):619-23, 339-40. English, Portuguese, Spanish. doi: 10.1016/S0034-7094(11)70073-3. PMID: 21920212.
772	21959734	Acyclovir	2011	Quiñones Ortiz L, Suárez Laurés A, Pobes Martínez A, de la Torre M, Torres Lacalle A, Forascepi Roza R. Warning against unexpected medication in haemodialysis. Nefrologia. 2011;31(5):611-2. English, Spanish. doi: 10.3265/Nefrologia.pre2011.Jun.10921. PMID: 21959734.
773	22010456	Moclobemide, venlafaxine, mianserine	2011	Majewska M, Szponar J, Pyra E, Kostek H, Kujawa A. Zespół serotoninergiczny w przebiegu zatrucia lekami--opis przypadku [Serotonin syndrome in the course of drug-poisoning--case presentation]. Przegl Lek. 2011;68(8):523-6. Polish. PMID: 22010456.
774	22196307	Olanzapine	2012	Rosen JB, Milstein MJ, Haut SR. Olanzapine-associated myoclonus. Epilepsy Res. 2012 Feb;98(2-3):247-50. doi: 10.1016/j.eplepsyres.2011.07.020. Epub 2011 Dec 21. PMID: 22196307.
775	22279347	Gabapentin	2012	Guddati AK, Zafar Z, Cheng JT, Mohan S. Treatment of gabapentin-induced myoclonus with continuous renal replacement therapy. Indian J Nephrol. 2012 Jan;22(1):59-61. doi: 10.4103/0971-4065.83744. PMID: 22279347; PMCID: PMC3263068.
776	22294022	Aciclovir, valaciclovir	2012	Ruiz-Roso G, Gomis A, Fernández-Lucas M, Díaz-Domínguez M, Teruel-Briones JL, Quereda C. Aciclovir and valaciclovir neurotoxicity in patients with renal failure. Nefrologia. 2012;32(1):114-5. English, Spanish. doi: 10.3265/Nefrologia.pre2011.Nov.11247. PMID: 22294022.

777	22421702	Amantadine	2012	Yarnall AJ, Burn DJ. Amantadine-induced myoclonus in a patient with progressive supranuclear palsy. Age Ageing. 2012 Sep;41(5):695-6. doi: 10.1093/ageing/afs043. Epub 2012 Mar 15. PMID: 22421702.
778	22440086	Sertraline	2012	Bušková J, Vorlová T, Piško J, Sonka K. Severe sleep-related movement disorder induced by sertraline. Sleep Med. 2012 Jun;13(6):769-70. doi: 10.1016/j.sleep.2012.01.006. Epub 2012 Mar 21. PMID: 22440086.
779	22456301	Oxcarbazepine	2012	Deng S, Luo R, Mao M, Huang L. Myoclonus precipitated by oral suspension of oxcarbazepine in idiopathic generalized epilepsy. Int J Clin Pharmacol Ther. 2012 Apr;50(4):300-1. doi: 10.5414/cp201660. PMID: 22456301.
780	22584239	Carbamazepine	2012	Magaudda A, Di Rosa G. Carbamazepine-induced non-epileptic myoclonus and tic-like movements. Epileptic Disord. 2012 Jun;14(2):172-3. doi: 10.1684/epd.2012.0504. PMID: 22584239.
781	22591802	Cobalamin	2012	Zanus C, Alberini E, Costa P, Colonna F, Zennaro F, Carrozzi M. Involuntary movements after correction of vitamin B12 deficiency: a video-case report. Epileptic Disord. 2012 Jun;14(2):174-80. doi: 10.1684/epd.2012.0507. PMID: 22591802.
782	22719217	Escitalopram, cocaine	2012	Malik HU, Kumar K. Serotonin syndrome with escitolapram and concomitant use of cocaine: a case report. Clin Med Insights Case Rep. 2012;5:81-5. doi: 10.4137/CCRep.S9540. Epub 2012 May 30. PMID: 22719217; PMCID: PMC3377381.
783	22762475	Etomidate	2012	Sneyd JR. Novel etomidate derivatives. Curr Pharm Des. 2012;18(38):6253-6. doi: 10.2174/138161212803832362. PMID: 22762475.
784	22805231	Levetiracetam	2012	Babtain FA. Levetiracetam may worsen myoclonus in patients with juvenile myoclonic epilepsy: case reports. Clin Neuropharmacol. 2012 Jul-Aug;35(4):201-2. doi: 10.1097/WNF.0b013e31825eed8c. PMID: 22805231.
785	22837118	Meperidine	2012	Abdollahi MH, Forouzannia SK, Bagherinasab M, Barzegar K, Fekri A, Sarebanhassanabadi M, Entezari A. The effect of ondansetron and meperedin on preventing shivering after off-pump coronary artery bypass graft. Acta Med Iran. 2012;50(6):395-8. PMID: 22837118.
786	22922060	Cobalamin	2013	Abourazzak S, Chaouki S, Boubou M, Amrani M, Atmani S, Hida M. Mouvements involontaires apparus chez un nourrisson traité pour une carence en vitamine B12 [Involuntary movements in infantile cobalamin deficiency appearing during treatment]. Presse Med. 2013 Apr;42(4 Pt 1):479-82. French. doi: 10.1016/j.lpm.2012.06.012. Epub 2012 Aug 24. PMID: 22922060.
787	22925158	Morphine, hydromorphone	2013	Juba KM, Wahler RG, Daron SM. Morphine and hydromorphone-induced hyperalgesia in a hospice patient. J Palliat Med. 2013 Jul;16(7):809-12. doi: 10.1089/jpm.2011.0502. Epub 2012 Aug 27. PMID: 22925158.
788	22925591	Opioid	2013	Jackson TP, Lonergan DF, Todd RD, Martin PR. Intentional intrathecal opioid detoxification in 3 patients: characterization of the intrathecal opioid withdrawal syndrome. Pain Pract. 2013 Apr;13(4):297-309. doi: 10.1111/j.1533-2500.2012.00584.x. Epub 2012 Aug 28. PMID: 22925591.
789	22995543	Etomidate	2013	Wu J, Yao S, Wu Z, Wu Z, Chu S, Xia G, Deng F. A comparison of anesthetic regimens using etomidate and propofol in patients undergoing first-trimester abortions: double-blind, randomized clinical trial of safety and efficacy. Contraception. 2013 Jan;87(1):55-62. doi: 10.1016/j.contraception.2012.08.014. Epub 2012 Sep 17. PMID: 22995543.
790	22995672	Morphine	2013	Gretton SK, Ross JR, Rutter D, Sato H, Droney JM, Welsh KI, Joel S, Riley J. Plasma morphine and metabolite concentrations are associated with clinical effects of morphine in cancer patients. J Pain Symptom Manage. 2013 Apr;45(4):670-80. doi: 10.1016/j.jpainsymman.2012.03.015. Epub 2012 Sep 18. PMID: 22995672.
791	23001110	Organophosphate	2012	Rajasekharan C, Renjith SW, Jayapal T. Opsoclonus and lingual myoclonus due to organophosphate poisoning: images in clinical medicine. BMJ Case Rep. 2012 Sep 21;2012:bcr2012007043. doi: 10.1136/bcr-2012-007043. PMID: 23001110; PMCID: PMC8029556.
792	23037679	Haloperidol	2012	Vural A, Tezer FI. Myoclonus induced by haloperidol in the intensive care unit. J Neuropsychiatry Clin Neurosci. 2012 Summer;24(3):E41. doi: 10.1176/appi.neuropsych.11080179. PMID: 23037679.
793	23062245	Olanzapine	2012	Singh LK, Praharaj SK, Sahu M. Nonfatal suicidal overdose of olanzapine in an adolescent. Curr Drug Saf. 2012 Sep;7(4):328-9. PMID: 23062245.
794	23066555	Amiodarone	2012	Deik AF, Shanker VL. A case of amiodarone-associated myoclonus responsive to levetiracetam. Can J Neurol Sci. 2012 Sep;39(5):680-1. doi: 10.1017/s0317167100018229. PMID: 23066555.
795	23114671	Gentamicin	2012	Sarva H, Panichpisal K. Gentamicin-induced myoclonus: a case report and literature review of antibiotics-induced myoclonus. Neurologist. 2012 Nov;18(6):385-8. doi: 10.1097/NRL.0b013e3182704d78. PMID: 23114671.
796	23144108	Carbamazepine	2012	Azad C, Guglani V. Eyelid myoclonia with absence seizure precipitated by carbamazepine therapy. Indian Pediatr. 2012 Oct;49(10):840-1. doi: 10.1007/s13312-012-0173-7. PMID: 23144108.
797	23192584	Pregabalin	2012	Shimizu T, Yoshida T, Kitamura K, Hamada O. Disturbance of consciousness and involuntary movements caused by pregabalin. BMJ Case Rep. 2012 Nov 28;2012:bcr2012007559. doi: 10.1136/bcr-2012-007559. PMID: 23192584; PMCID: PMC4544218.
798	23364782	Meperidine	2012	Ozkaya H, Akcan AB, Aydemir G, Akbaş M. Düşük doz meperidin uygulamasına bağlı konvulsiyon: Olgu sunumu [Convulsion due to application of low dose meperidine: a case report]. Agri. 2012;24(4):187-90. Turkish. doi: 10.5505/agri.2012.68542. PMID: 23364782.
799	23372880	Etomidate	2013	Ko BJ, Oh JN, Lee JH, Choi SR, Lee SC, Chung CJ. Comparison of effects of fentanyl and remifentanil on hemodynamic response to endotracheal intubation and myoclonus in elderly patients with etomidate induction. Korean J Anesthesiol. 2013 Jan;64(1):12-8. doi: 10.4097/kjae.2013.64.1.12. Epub 2013 Jan 21. PMID: 23372880; PMCID: PMC3558642.
800	23391344	Propofol, remifentanil	2013	Davis JJ, Buck NS, Swenson JD, Johnson KB, Greis PE. Serotonin syndrome manifesting as patient movement during total intravenous anesthesia with propofol and remifentanil. J Clin Anesth. 2013 Feb;25(1):52-4. doi: 10.1016/j.jclinane.2012.05.002. PMID: 23391344.
801	23430549	Ceftriaxone	2013	Sechi G, Ceccherini I, Bachetti T, Deiana GA, Sechi E, Balbi P. Ceftriaxone for Alexander's Disease: A Four-Year Follow-Up. JIMD Rep. 2013;9:67-71. doi: 10.1007/8904_2012_180. Epub 2012 Oct 13. PMID: 23430549; PMCID: PMC3565626.
802	23450863	Morphine	2012	Iff I, Valeskini K, Mosing M. Severe pruritus and myoclonus following intrathecal morphine administration in a dog. Can Vet J. 2012 Sep;53(9):983-6. PMID: 23450863; PMCID: PMC3418785.

803	23558062	Etomidate	2013	Yates AM, Wolfson AB, Shum L, Kehrl T. A descriptive study of myoclonus associated with etomidate procedural sedation in the ED. Am J Emerg Med. 2013 May;31(5):852-4. doi: 10.1016/j.ajem.2013.02.042. Epub 2013 Apr 1. PMID: 23558062.
804	23599979	Etomidate	2013	Zou L, Yuan H, Wang HY, Geng ZY, Xu L, Sun L. [Role of target controlled infusion of remifentanil for the prevention of etomidate induced myoclonus during general anesthesia]. Zhongguo Yi Xue Ke Xue Yuan Xue Bao. 2013 Feb;35(1):112-5. Chinese. PMID: 23599979.
805	23686057	Disulfiram	2013	Sreejayan K, Praharaj SK. Myoclonus associated with disulfiram. J Neuropsychiatry Clin Neurosci. 2013 Spring;25(2):E37-9. doi: 10.1176/appi.neuropsych.12030073. PMID: 23686057.
806	23715067	Hydromorphone	2013	Gagnon DJ, Jwo K. Tremors and agitation following low-dose intravenous hydromorphone administration in a patient with kidney dysfunction. Ann Pharmacother. 2013 Jul-Aug;47(7-8):e34. doi: 10.1345/aph.1R784. Epub 2013 May 28. PMID: 23715067.
807	23774821	Oxcarbazepine	2013	Fanella M, Egeo G, Fattouch J, Casciato S, Lapenta L, Morano A, Giallonardo AT, Di Bonaventura C. Oxcarbazepine-induced myoclonic status epilepticus in juvenile myoclonic epilepsy. Epileptic Disord. 2013 Jun;15(2):181-7. doi: 10.1684/epd.2013.0563. PMID: 23774821.
808	23798279	Bismuth	2013	Masannat Y, Nazer E. Pepto bismuth associated neurotoxicity: A rare side effect of a commonly used medication. W V Med J. 2013 May-Jun;109(3):32-4. PMID: 23798279.
809	23869656	Lamotrigine	2013	Moore PW, Donovan JW, Burkhart KK, Haggerty D. A case series of patients with lamotrigine toxicity at one center from 2003 to 2012. Clin Toxicol (Phila). 2013 Aug;51(7):545-9. doi: 10.3109/15563650.2013.818685. Epub 2013 Jul 19. PMID: 23869656.
810	23879446	Ciprofloxacin, oxycodone	2014	Kango Gopal G, Hewton C, Pazhvoor SK. Myoclonus associated with concomitant ciprofloxacin and oxycodone in an older patient. Br J Clin Pharmacol. 2014 May;77(5):906-7. doi: 10.1111/bcp.12213. PMID: 23879446; PMCID: PMC4004412.
811	23896816	Lacosamide	2014	Legros B, Depondt C, Levy-Nogueira M, Ligot N, Mavroudakis N, Naeije G, Gaspard N. Intravenous lacosamide in refractory seizure clusters and status epilepticus: comparison of 200 and 400 mg loading doses. Neurocrit Care. 2014 Jun;20(3):484-8. doi: 10.1007/s12028-013-9882-6. PMID: 23896816.
812	23900376	Azithromycin	2013	Vadalá SF, Pellegrini D, Silva ED, Miñarro D, Finn BC, Bruetman JE, Nápoli G, Young P. Enfermedad de Von Economo: Caso clínico [Lethargic encephalitis. Report of one case]. Rev Med Chil. 2013 Apr;141(4):531-4. Spanish. doi: 10.4067/S0034-98872013000400016. PMID: 23900376.
813	23936682	Peripheral nerve block	2013	Hudson AJ, Guthmiller KB, Hyatt MN. Myoclonus following a Peripheral Nerve Block. Case Rep Anesthesiol. 2013;2013:213472. doi: 10.1155/2013/213472. Epub 2013 Jul 11. PMID: 23936682; PMCID: PMC3725761.
814	23936696	Ranolazine	2013	Porhomayon J, Zadeii G, Yarahmadi A. A rare neurological complication of ranolazine. Case Rep Neurol Med. 2013;2013:451206. doi: 10.1155/2013/451206. Epub 2013 Jul 1. PMID: 23936696; PMCID: PMC3713362.
815	23986051	Fluvoxamine	2013	Takahashi C, Goto E, Taira S, Kataoka N, Nishihara M, Katsumata T, Goto I, Takiuchi H. [Serotonin syndrome in a patient with small cell lung cancer]. Gan To Kagaku Ryoho. 2013 Aug;40(8):1059-61. Japanese. PMID: 23986051.
816	24094859	Bromomethane (methyl bromide)	2013	de Souza A, Narvencar KP, Sindhoora KV. The neurological effects of methyl bromide intoxication. J Neurol Sci. 2013 Dec 15;335(1-2):36-41. doi: 10.1016/j.jns.2013.09.022. Epub 2013 Sep 20. PMID: 24094859.
817	24200036	Cefepime	2013	Fugate JE, Kalimullah EA, Hocker SE, Clark SL, Wijdicks EF, Rabinstein AA. Cefepime neurotoxicity in the intensive care unit: a cause of severe, underappreciated encephalopathy. Crit Care. 2013 Nov 7;17(6):R264. doi: 10.1186/cc13094. PMID: 24200036; PMCID: PMC4057506.
818	24238149	Quetiapine	2013	George M, Haasz M, Coronado A, Salhanick S, Korbel L, Kitzmiller JP. Acute dyskinesia, myoclonus, and akathisa in an adolescent male abusing quetiapine via nasal insufflation: a case study. BMC Pediatr. 2013 Nov 16;13:187. doi: 10.1186/1471-2431-13-187. PMID: 24238149; PMCID: PMC4225618.
819	24347782	Piperazine	2013	Abiramalatha T, Mehndiratta S, Rajeshwari K, Dubey AP. Piperazine citrate induced myoclonus in a child. Indian J Pharmacol. 2013 Nov-Dec;45(6):640. doi: 10.4103/0253-7613.121391. PMID: 24347782; PMCID: PMC3847264.
820	24483863	Etanercept	2014	Domínguez Leñero V, Sánchez-Martínez I, Giménez-Cortés ME, Valiente-Borrego F. Mioclonias cervicales en paciente en tratamiento con etanercept [Myoclonus in patients treated with etanercept]. Farm Hosp. 2014 Jan 1;38(1):69-70. Spanish. doi: 10.7399/FH.2014.38.1.1109. PMID: 24483863.
821	24506579	Cefepime	2014	Ugai T, Morisaki K, Tsuda K, Sugihara H, Nishida Y, Yamakura M, Takeuchi M, Matsue K. Cefepime-induced encephalopathy in patients with haematological malignancies: clinical features and risk factors. Scand J Infect Dis. 2014 Apr;46(4):272-9. doi: 10.3109/00365548.2013.878032. Epub 2014 Feb 10. PMID: 24506579.
822	24529669	Etomidate	2013	Nooraei N, Solhpour A, Mohajerani SA. Priming with atracurium efficiently suppresses etomidate-induced myoclonus. Acta Anaesthesiol Taiwan. 2013 Dec;51(4):145-8. doi: 10.1016/j.aat.2013.12.005. Epub 2014 Jan 20. PMID: 24529669.
823	24535067	Etomidate	2014	Isitemiz I, Uzman S, Toptaş M, Vahapoglu A, Gül YG, Inal FY, Akkoc I. Prevention of etomidate-induced myoclonus: which is superior: Fentanyl, midazolam, or a combination? A Retrospective comparative study. Med Sci Monit. 2014 Feb 16;20:262-7. doi: 10.12659/MSM.889833. PMID: 24535067; PMCID: PMC3930766.
824	24548550	Pentylenetetrazole	2014	Aksoy D, Solmaz V, Erbas O. Positive effect of calcitonin on the seizures induced by pentylenetetrazole in rats. Epilepsy Res. 2014 Mar;108(3):390-5. doi: 10.1016/j.eplepsyres.2014.01.012. Epub 2014 Jan 30. PMID: 24548550.
825	24614665	Flecainide	2014	Velasco SL, Sierra-Hidalgo F, Rodríguez RM, Guerreo AJ, Morales JR. Flecainide-induced myoclonus. Clin Neuropharmacol. 2014 Mar-Apr;37(2):65-6. doi: 10.1097/WNF.0000000000000025. PMID: 24614665.
826	24614666	Lamotrigine	2014	Algahtani HA, Aldarmahi AA, Al-Rabia MW, Almalki WH, Bryan Young G. Generalized myoclonus and spasticity induced by lamotrigine toxicity: a case report and literature review. Clin Neuropharmacol. 2014 Mar-Apr;37(2):52-4. doi: 10.1097/WNF.0000000000000020. PMID: 24614666.
827	24828848	Etomidate	2014	Lv Z, Fang J, Zhu J, Liang B, Li F, Jiang S, Li A, Cheng Z, Dong T. Intravenous dezocine pretreatment reduces the incidence and intensity of myoclonus induced by etomidate. J Anesth. 2014 Dec;28(6):944-7. doi: 10.1007/s00540-014-1842-6. Epub 2014 May 15. Erratum in: J Anesth. 2014 Dec;28(6):948. Lu, Zhifeng [Corrected to Lv, Zhifeng]. PMID: 24828848.
828	24852503	Cobalamin	2014	de Souza A, Moloi MW. Involuntary movements due to vitamin B12 deficiency. Neurol Res. 2014 Dec;36(12):1121-8. doi:

				10.1179/1743132814Y.0000000396. Epub 2014 May 22. PMID: 24852503.
829	24858231	Etomidate	2015	He L, Ding Y, Chen H, Qian Y, Li Z. Dezocine pretreatment prevents myoclonus induced by etomidate: a randomized, double-blinded controlled trial. J Anesth. 2015 Feb;29(1):143-5. doi: 10.1007/s00540-014-1854-2. Epub 2014 May 25. PMID: 24858231.
830	24937115	Sulpiride	2002	Asahi S, Nishikawa T, Kurata K, Morinobu S, Yamawaki S. A case of myclonus, resembling epileptic seizure, induced by short-term sulpiride treatment. Int J Psychiatry Clin Pract. 2002;6(4):215-6. doi: 10.1080/136515002761581027. PMID: 24937115.
831	24961940	Propofol	2015	Cattai A, Rabozzi R, Natale V, Franci P. The incidence of spontaneous movements (myoclonus) in dogs undergoing total intravenous anaesthesia with propofol. Vet Anaesth Analg. 2015 Jan;42(1):93-8. doi: 10.1111/vaa.12160. Epub 2014 Jun 25. PMID: 24961940.
832	24987501	Piperacillin, nafcillin	2014	Meyer MA. Myoclonic jerks secondary to piperacillin and nafcillin. Neurol Int. 2014 Jun 16;6(2):5349. doi: 10.4081/ni.2014.5349. PMID: 24987501; PMCID: PMC4077207.
833	25066813	Meropenem	2014	Spina Silva T, Dal-Prá Ducci R, Zorzetto FP, Braatz VL, de Paola L, Kowacs PA. Meropenem-induced myoclonus: a case report. Seizure. 2014 Nov;23(10):912-4. doi: 10.1016/j.seizure.2014.06.017. Epub 2014 Jul 10. PMID: 25066813.
834	25103080	Gabapentin	2014	Shea YF, Mok MM, Chang RS. Gabapentin-induced myoclonus in an elderly with end-stage renal failure. J Formos Med Assoc. 2014 Sep;113(9):660-1. doi: 10.1016/j.jfma.2012.06.001. Epub 2012 Sep 3. PMID: 25103080.
835	25202036	Donepezil	2014	Bougea A, Gerakoulis S, Anagnostou E, Paraskevas G, Kapaki E, Kararizou E. Donepezil-induced myoclonus in a patient with Alzheimer disease. Ann Pharmacother. 2014 Dec;48(12):1659-61. doi: 10.1177/1060028014550334. Epub 2014 Sep 8. PMID: 25202036.
836	25203795	Risperidone, trihexyphenidyl and benzodiazepines	2014	Hsu YC, Yeh YW. Multidrug overdose-induced myoclonus complicated by rhabdomyolysis: possible role and mechanism of muscle toxicity of risperidone. J Clin Pharm Ther. 2014 Dec;39(6):698-700. doi: 10.1111/jcpt.12205. Epub 2014 Sep 9. PMID: 25203795.
837	25288698	Memantine	2014	Kitagawa N, Takeuchi A. Memantine-induced myoclonus. Neurology. 2014 Oct 7;83(15):1387. doi: 10.1212/WNL.0000000000000863. PMID: 25288698.
838	25309313	Pentylenetetrazole	2014	García-Cabrero AM, Sánchez-Elexpuru G, Serratosa JM, Sánchez MP. Enhanced sensitivity of laforin- and malin-deficient mice to the convulsant agent pentylenetetrazole. Front Neurosci. 2014 Sep 12;8:291. doi: 10.3389/fnins.2014.00291. PMID: 25309313; PMCID: PMC4162417.
839	25314987	Caesium chloride	2014	Warsame MO, Gamboa D, Nielsen EW. En kvinne i 40-årene med kreft, synkope og kramper [A woman in her forties with cancer, syncope and spasms]. Tidsskr Nor Laegeforen. 2014 Oct 14;134(19):1855-7. Norwegian. doi: 10.4045/tidsskr.13.1528. PMID: 25314987.
840	25317545	Etomidate	2014	He L, Ding Y, Chen H, Qian Y, Li Z. Butorphanol pre-treatment prevents myoclonus induced by etomidate: a randomised, double-blind, controlled clinical trial. Swiss Med Wkly. 2014 Oct 15;144:w14042. doi: 10.4414/smw.2014.14042. PMID: 25317545.
841	25351237	Etomidate	2015	Luan HF, Zhao ZB, Feng JY, Cui JZ, Zhang XB, Zhu P, Zhang YH. Prevention of etomidate-induced myoclonus during anesthetic induction by pretreatment with dexmedetomidine. Braz J Med Biol Res. 2015 Feb;48(2):186-90. doi: 10.1590/1414-431X20144100. Epub 2014 Oct 24. PMID: 25351237; PMCID: PMC4321226.
842	25479320	Pentylenetetrazol	2016	Uyanikgil Y, Özkeşkek K, Çavuşoğlu T, Solmaz V, Tümer MK, Erbas O. Positive effects of ceftriaxone on pentylenetetrazol-induced convulsion model in rats. Int J Neurosci. 2016;126(1):70-5. doi: 10.3109/00207454.2014.991821. Epub 2015 May 22. PMID: 25479320.
843	25499271	Fentanyl	2015	Larson KJ, Wittwer ED, Nicholson WT, Weingarten TN, Price DL, Sprung J. Myoclonus in patient on fluoxetine after receiving fentanyl and low-dose methylene blue during sentinel lymph node biopsy. J Clin Anesth. 2015 May;27(3):247-51. doi: 10.1016/j.jclinane.2014.11.002. Epub 2014 Dec 11. PMID: 25499271.
844	25540528	Phenytoin	2014	Verma R, Kumar S, Biyani S, Singh A. Opsoclonus - Myoclonus syndrome induced by phenytoin intoxication. J Neurosci Rural Pract. 2014 Nov;5(Suppl 1):S109-10. doi: 10.4103/0976-3147.145254. PMID: 25540528; PMCID: PMC4271371.
845	25553506	Etomidate	2015	Shen XC, Ao X, Cao Y, Lan L, Liu XM, Sun WJ, Li P, Lan CH. Etomidate-remifentanil is more suitable for monitored anesthesia care during gastroscopy in older patients than propofol-remifentanil. Med Sci Monit. 2015 Jan 1;21:1-8. doi: 10.12659/MSM.891183. PMID: 25553506; PMCID: PMC4288392.
846	25671244	Metaxalone	2015	Martini DI, Nacca N, Haswell D, Cobb T, Hodgman M. Serotonin syndrome following metaxalone overdose and therapeutic use of a selective serotonin reuptake inhibitor. Clin Toxicol (Phila). 2015 Mar;53(3):185-7. doi: 10.3109/15563650.2015.1009993. Epub 2015 Feb 11. PMID: 25671244.
847	25673664	Etomidate	2015	d'Ovidio D, Spadavecchia C, Angeli G, Adami C. Etomidate anaesthesia by immersion in oriental fire-bellied toads (Bombina orientalis). Lab Anim. 2015 Oct;49(4):319-26. doi: 10.1177/0023677215571655. Epub 2015 Feb 11. PMID: 25673664.
848	25679129	Clozapine	2015	Takahashi T, Masuya Y, Ueno K, Watanabe K, Takahashi M, Morita S, Higashima M, Wada Y. Clozapine-related negative myoclonus associated with urinary tract infection: a case report. J Clin Psychopharmacol. 2015 Apr;35(2):205-6. doi: 10.1097/JCP.0000000000000290. Erratum in: J Clin Psychopharmacol. 2015 Aug;35(4):381. doi: 10.1097/JCP.0000000000000365. Morita, Machiyo [corrected to Morita, Sachiyo]. PMID: 25679129.
849	25684877	Ceftazidime	2015	Joseph J, Vimala A. Ceftazidime-induced myoclonus and encephalopathy in hemodialysis patient. Indian J Nephrol. 2015 Jan-Feb;25(1):61-2. doi: 10.4103/0971-4065.144426. PMID: 25684877; PMCID: PMC4323917.
850	25780020	Cefepime	2015	Khasani S. Cefepime-induced jaw myoclonus. Neurology. 2015 Mar 17;84(11):1183. doi: 10.1212/WNL.0000000000001365. PMID: 25780020.
851	25784625	Acyclovir	2015	Gentry JL 3rd, Peterson C. Death Delusions and Myoclonus: Acyclovir Toxicity. Am J Med. 2015 Jul;128(7):692-4. doi: 10.1016/j.amjmed.2015.03.001. Epub 2015 Mar 14. PMID: 25784625.
852	25802272	Metoclopramide	2015	Immovilli P, Rota E, Morelli N, Iafelice I, Magnacavallo A, Guidetti D. Metoclopramide-induced facial and palatopharyngeal myoclonus. Neurology. 2015 Mar 24;84(12):1284. doi: 10.1212/WNL.0000000000001393. PMID: 25802272.
853	25840854	Epidural analgesia	2015	Ji TT, Shih CK, Hsieh YJ, Sun WZ. Desultory propriospinal myoclonus after epidural analgesia in a healthy parturient. Int J Obstet

				Anesth. 2015 Aug;24(3):285-6. doi: 10.1016/j.ijoa.2015.02.009. Epub 2015 Feb 26. PMID: 25840854.
854	25867876	Levobupivacaine	2015	Akelçi K, Atıcı Ş. Epidural levobupivakain infüzyonuna bağlı geçici spinal miyoklonus: Olgu sunumu [Transient spinal myoclonus due to epidural levobupivacaine infusion: case report]. Agri. 2015;27(1):58-60. Turkish. doi: 10.5505/agri.2015.45077. PMID: 25867876.
855	25896869	Midazolam	2015	Ozcan B, Kavurt S, Yucel H, Bas AY, Demirel N. Rhythmic myoclonic jerking induced by midazolam in a preterm infant. Pediatr Neurol. 2015 Jun;52(6):e9. doi: 10.1016/j.pediatrneurol.2015.02.019. Epub 2015 Mar 2. PMID: 25896869.
856	25948979	Olanzapine	2014	Tikka SK, Pratap A, Sinha VK. Dose-dependent olanzapine-induced myoclonus. Toxicol Int. 2014 Sep-Dec;21(3):335-6. doi: 10.4103/0971-6580.155393. PMID: 25948979; PMCID: PMC4413423.
857	25956160	Piperacillin/tazobactam	2015	Man BL, Fu YP. Piperacillin/tazobactam-induced myoclonic jerks in a man with chronic renal failure. BMJ Case Rep. 2015 May 7;2015:bcr2015210184. doi: 10.1136/bcr-2015-210184. PMID: 25956160; PMCID: PMC4434266.
858	25956204	Pregabalin	2015	Olszewska DA, Chalissery AJ, Williams J, Lynch T, Smyth S. Speech myoclonus due to probable pregabalin adverse drug-reaction. Parkinsonism Relat Disord. 2015 Jul;21(7):823-4. doi: 10.1016/j.parkreldis.2015.04.026. Epub 2015 May 2. PMID: 25956204.
859	26111222	trimethoprim-sulfamethoxazole	2016	Gray DA, Foo D. Reversible myoclonus, asterixis, and tremor associated with high dose trimethoprim-sulfamethoxazole: a case report. J Spinal Cord Med. 2016;39(1):115-7. doi: 10.1179/2045772315Y.0000000018. Epub 2015 Jun 25. PMID: 26111222; PMCID: PMC4725781.
860	26114261	Clozapine	2015	Erratum: Clozapine-Related Negative Myoclonus Associated With Urinary Tract Infection. J Clin Psychopharmacol. 2015 Aug;35(4):381. doi: 10.1097/JCP.0000000000000365. Erratum for: J Clin Psychopharmacol. 2015 Apr;35(2):205-6. doi: 10.1097/JCP.0000000000000290. Morita, Machiyo [corrected to Morita, Sachiyo]. PMID: 26114261.
861	26123247	Dextromethorphan, chlorphenamine	2017	Velázquez A, Santos S, Bellosta E, Iñiguez C. Myoclonus secondary to use of anti-flu drug. Neurologia. 2017 Mar;32(2):133-135. English, Spanish. doi: 10.1016/j.nrl.2015.05.004. Epub 2015 Jun 27. PMID: 26123247.
862	26139733	Etomidate	2015	Desai PM, Kane D, Sarkar MS. Cardioversion: What to choose? Etomidate or propofol. Ann Card Anaesth. 2015 Jul-Sep;18(3):306-11. doi: 10.4103/0971-9784.159798. PMID: 26139733; PMCID: PMC4881724.
863	26234740	Opioid	2016	Winegarden J, Carr DB, Bradshaw YS. Intravenous Ketamine for Rapid Opioid Dose Reduction, Reversal of Opioid-Induced Neurotoxicity, and Pain Control in Terminal Care: Case Report and Literature Review. Pain Med. 2016 Apr;17(4):644-9. doi: 10.1111/pme.12865. Epub 2016 Jan 28. PMID: 26234740.
864	26293915	Clozapine	2015	Praharaj SK, Vemanna N, Sharma PS. Knee buckling (negative myoclonus) associated with clozapine: Is there a dose threshold? Clin Toxicol (Phila). 2015 Nov;53(9):918-9. doi: 10.3109/15563650.2015.1079326. Epub 2015 Aug 20. PMID: 26293915.
865	26317045	Memantine	2015	Murgai AA, LeDoux MS. Memantine-induced Myoclonus in a Patient with Alzheimer Disease. Tremor Other Hyperkinet Mov (N Y). 2015 Aug 14;5:337. doi: 10.7916/D8ZG6RD9. PMID: 26317045; PMCID: PMC4548970.
866	26318583	Fentanyl	2016	López Pardo P, Izquierdo Zamarriego G. Mioclonías por fentanilo. A propósito de un caso [Myoclonus due to fentanyl. Report of a case]. Rev Esp Geriatr Gerontol. 2016 Jan-Feb;51(1):60-1. Spanish. doi: 10.1016/j.regg.2015.07.006. Epub 2015 Aug 28. PMID: 26318583.
867	26413243	Cefepime	2015	Khasani S, Gill S, Semenova L, Sarva H. Predominant Jaw Myoclonus from Cefepime Toxicity: A Case Report and a Review of the Literature. J Mov Disord. 2015 Sep;8(3):144-6. doi: 10.14802/jmd.15017. Epub 2015 Sep 10. PMID: 26413243; PMCID: PMC4572666.
868	26418639	Citalopram	2015	Forsberg-Gillving M, Bode M, Sindrup SH. Patient med nedsat leverfunktion fikmyoklonier under behandling medselektive serotoningenoptagelseshæmmere [Myoclonus as a side effect to citalopram treatment in a patient with liver cirrhosis]. Ugeskr Laeger. 2015 Sep 21;177(39):V04150325. Danish. PMID: 26418639.
869	26475124	Ceftazidime	2016	Collins RD, Tverdek FP, Bruno JJ, Coyle EA. Probable Nonconvulsive Status Epilepticus With the Use of High-Dose Continuous Infusion Ceftazidime. J Pharm Pract. 2016 Dec;29(6):564-568. doi: 10.1177/0897190015608503. Epub 2015 Oct 16. PMID: 26475124.
870	26491328	Escitalopram	2015	Sato Y, Nakamura K, Yasui-Furukori N. Serotonin syndrome induced by the readministration of escitalopram after a short-term interruption in an elderly woman with depression: a case report. Neuropsychiatr Dis Treat. 2015 Sep 30;11:2505-7. doi: 10.2147/NDT.S92081. PMID: 26491328; PMCID: PMC4599182.
871	26678295	Fentanyl	2016	Ringer SK, Spielmann N, Weiss M, Mauch JY. Fentanyl bolus induces muscle tremors in sevoflurane-anaesthetized piglets. Lab Anim. 2016 Aug;50(4):312-4. doi: 10.1177/0023677215623896. Epub 2015 Dec 16. PMID: 26678295.
872	26691710	Memantine	2015	Pei LJ, Tianzhi IL, Lim WS. Memantine-Induced Myoclonus Precipitated by Renal Impairment and Drug Interactions. J Am Geriatr Soc. 2015 Dec;63(12):2643-2644. doi: 10.1111/jgs.13847. PMID: 26691710.
873	26718619	Cefepime	2015	Honore PM, Spapen HD. Cefepime-induced neurotoxicity in critically ill patients undergoing continuous renal replacement therapy: beware of dose reduction! Crit Care. 2015 Dec 30;19:455. doi: 10.1186/s13054-015-1179-z. PMID: 26718619; PMCID: PMC4699351.
874	26723966	Imipenem	2016	Gschwind M, Simonetta F, Vulliemoz S. Reversible encephalopathy with photoparoxysmal response during imipenem/cilastatin treatment. J Neurol Sci. 2016 Jan 15;360:23-4. doi: 10.1016/j.jns.2015.11.038. Epub 2015 Nov 21. PMID: 26723966.
875	26834968	Clozapine	2015	Osborne IJ, McIvor RJ. Clozapine-induced myoclonus: a case report and review of the literature. Ther Adv Psychopharmacol. 2015 Dec;5(6):351-6. doi: 10.1177/2045125315612015. PMID: 26834968; PMCID: PMC4722506.
876	26840341	Etomidate	2018	Wang J, Li QB, Wu YY, Wang BN, Kang JL, Xu XW. Efficacy and Safety of Opioids for the Prevention of Etomidate-Induced Myoclonus: A Meta-Analysis. Am J Ther. 2018 Sep/Oct;25(5):e517-e523. doi: 10.1097/MJT.0000000000000404. PMID: 26840341.
877	26845815	Lidocaine (lignocaine)	2012	Kituu N, Mugenya WO, Kituyi PW. Central Nervous System Lignocaine Toxicity In An Infant Following Ventriculo-Peritoneal Shunt And Spina Bifida Repair: A Case Report. East Afr Med J. 2012 Feb;89(2):71-2. PMID: 26845815.
878	26871805	Etomidate	2016	Wu GN, Xu HJ, Liu FF, Wu X, Zhou H. Low-Dose Ketamine Pretreatment Reduces the Incidence and Severity of Myoclonus

				Induced by Etomidate: A Randomized, Double-Blinded, Controlled Clinical Trial. Medicine (Baltimore). 2016 Feb;95(6):e2701. doi: 10.1097/MD.0000000000002701. PMID: 26871805; PMCID: PMC4753901.
879	26888997	Antibiotic	2016	Bhattacharyya S, Darby RR, Raibagkar P, Gonzalez Castro LN, Berkowitz AL. Antibiotic-associated encephalopathy. Neurology. 2016 Mar 8;86(10):963-71. doi: 10.1212/WNL.0000000000002455. Epub 2016 Feb 17. Erratum in: Neurology. 2016 May 31;86(22):2116. doi: 10.1212/WNL.0000000000002754. PMID: 26888997.
880	26922134	Antipsychotic	2016	Seigneurie AS, Sauvanaud F, Limosin F. Dyskinésies tardives induites par les antipsychotiques : données actuelles sur leur prévention et prise en charge [Prevention and treatment of tardive dyskinesia caused by antipsychotic drugs]. Encephale. 2016 Jun;42(3):248-54. French. doi: 10.1016/j.encep.2015.12.021. Epub 2016 Feb 26. PMID: 26922134.
881	26993408	Etomidate	2016	Aggarwal S, Goyal VK, Chaturvedi SK, Mathur V, Baj B, Kumar A. Estudo comparativo entre propofol e etomidato em pacientes sob anestesia geral [A comparative study between propofol and etomidate in patients under general anesthesia]. Rev Bras Anestesiol. 2016 May-Jun;66(3):237-41. Portuguese. doi: 10.1016/j.bjan.2016.02.010. Epub 2016 Mar 15. PMID: 26993408.
882	27006537	Etomidate	2016	Alipour M, Tabari M, Azad AM. Comparative study evaluating efficacy of sufentanil versus midazolam in preventing myoclonic movements following etomidate. J Anaesthesiol Clin Pharmacol. 2016 Jan-Mar;32(1):29-32. doi: 10.4103/0970-9185.173382. PMID: 27006537; PMCID: PMC4784209.
883	27095811	Snake bite	2016	Ramcharan K, Abdool K, Persad N, Alexander A. Snake bite-induced myoclonus, myokymia and myospasm with leukoencephalopathy: a video presentation. BMJ Case Rep. 2016 Apr 19;2016:10.1136/bcr-2016-214963. doi: 10.1136/bcr-2016-214963. PMID: 27095811; PMCID: PMC4840739.
884	27114634	Alcohol	2016	Prakash S, Balhara YP. Rare Form of Dyskinetic Movements Associated with Alcohol Withdrawal. Indian J Psychol Med. 2016 Mar-Apr;38(2):163-4. doi: 10.4103/0253-7176.178816. PMID: 27114634; PMCID: PMC4820561.
885	27155778	Etomidate	2016	Yılmaz Çakirgöz M, Demirel İ, Duran E, Özer AB, Hancı V, Türkmen ÜA, Aydın A, Ersoy A, Büyükyıldırım A. Efeito do pré-tratamento com gabapentina sobre a mioclonia após etomidato: um estudo randômico, duplo-cego e controlado por placebo [Effect of gabapentin pretreatment on myoclonus after etomidate: a randomized, double-blind, placebo-controlled study]. Rev Bras Anestesiol. 2016 Jul-Aug;66(4):356-62. Portuguese. doi: 10.1016/j.bjan.2016.04.008. Epub 2016 May 5. PMID: 27155778.
886	27241156	Etomidate	2016	Wang L, Li W, Xu R, Long L. [Meta analysis for the anesthesia effect and adverse reactions of etomidate and propofol on the painless abortion surgery]. Zhong Nan Da Xue Xue Bao Yi Xue Ban. 2016 Apr;41(4):427-33. Chinese. doi: 10.11817/j.issn.1672-7347.2016.04.015. PMID: 27241156.
887	27247915	Etomidate	2016	Sedighinejad A, Naderi Nabi B, Haghighi M, Biazar G, Imantalab V, Rimaz S, Zaridoost Z. Comparison of the Effects of Low-Dose Midazolam, Magnesium Sulfate, Remifentanil and Low-Dose Etomidate on Prevention of Etomidate-Induced Myoclonus in Orthopedic Surgeries. Anesth Pain Med. 2016 Apr 2;6(2):e35333. doi: 10.5812/aapm.35333. PMID: 27247915; PMCID: PMC4885461.
888	27264590	Cobalamin	2011	Tosun A, Aral YZ, Çeçen E, Aydoğdu A, Çetinkaya Çakmak B. Involuntary movement in infants during vitamin B12 treatment. Turk J Haematol. 2011 Dec 5;28(4):317-22. English. doi: 10.5152/tjh.2011.18. PMID: 27264590.
889	27274853	Cefepime	2016	Meillier A, Rahimian D. Cefepime-induced encephalopathy with normal renal function. Oxf Med Case Reports. 2016 Jun 1;2016(6):118-20. doi: 10.1093/omcr/omw042. PMID: 27274853; PMCID: PMC4887827.
890	27308256	Etomidate	2016	Salen P, Grossman M, Grossman M, Milazzo A, Stoltzfus J. A comparison of ketamine versus etomidate for procedural sedation for the reduction of large joint dislocations. Int J Crit Illn Inj Sci. 2016 Apr-Jun;6(2):79-84. doi: 10.4103/2229-5151.183022. PMID: 27308256; PMCID: PMC4901832.
891	27343784	Etomidate	2016	Yılmaz Çakirgöz M, Demirel İ, Duran E, Özer AB, Hancı V, Türkmen ÜA, Aydın A, Ersoy A, Büyükyıldırım A. Effect of gabapentin pretreatment on myoclonus after etomidate: a randomized, double-blind, placebo-controlled study. Braz J Anesthesiol. 2016 Jul-Aug;66(4):356-62. doi: 10.1016/j.bjane.2014.11.014. Epub 2015 Oct 20. PMID: 27343784.
892	27408805	Cephalosporins	2016	Bora I, Demir AB, Uzun P. Nonconvulsive status epilepticus cases arising in connection with cephalosporins. Epilepsy Behav Case Rep. 2016 May 20;6:23-7. doi: 10.1016/j.ebcr.2016.04.005. PMID: 27408805; PMCID: PMC4925880.
893	27502592	Amphetamine	2016	Michelis JP, Zimmermann J, Bedarf JR, Bundschuh R, Gaertner FC, Paus S. Facial movement disorder and dopamine imaging in a patient with amphetamine abuse. Parkinsonism Relat Disord. 2016 Oct;31:153-155. doi: 10.1016/j.parkreldis.2016.07.020. Epub 2016 Aug 2. PMID: 27502592.
894	27510560	Palonosetron	2016	Chaw SH, Chan L, Lee PK, Bakar JA, Rasiah R, Foo LL. Prolonged drug-induced myoclonus: is it related to palonosetron? J Anesth. 2016 Dec;30(6):1063-1066. doi: 10.1007/s00540-016-2228-8. Epub 2016 Aug 10. PMID: 27510560.
895	27764052	Clozapine	2016	Sepede G, Di Iorio G, Spano MC, Lorusso M, Sarchione F, Santacroce R, Salerno RM, Di Giannantonio M. A Case of Resistant Schizophrenia Successfully Treated With Clozapine/Long-acting Injectable Aripiprazole Combination. Clin Neuropharmacol. 2016 Nov/Dec;39(6):322-324. doi: 10.1097/WNF.0000000000000191. PMID: 27764052.
896	27894232	Ciprofloxacin	2016	Bueno Juana E, Vicente de Vera Floristán C, Gracia Gutiérrez A, Pérez Calvo JI, Tejero Juste C. Mioclonías asociadas al tratamiento con ciprofloxacino [Myoclonus associated with Ciprofloxacin therapy]. Farm Hosp. 2016 Nov 1;40(n06):622-623. Spanish. doi: 10.7399/fh.2016.40.6.10536. PMID: 27894232.
897	27904101	Milnacipran	2016	Koshiishi T, Okuyama K. [Probable Serotonin Syndrome and Withdrawal Symptoms Caused by Milnacipran]. Yakugaku Zasshi. 2016;136(12):1675-1679. Japanese. doi: 10.1248/yakushi.16-00032. PMID: 27904101.
898	28017210	Venlafaxine	2017	Necpál J, Skorvanek M. Opsoclonus-myoclonus ataxia syndrome secondary to venlafaxine intoxication. J Neurol Sci. 2017 Jan 15;372:19-20. doi: 10.1016/j.jns.2016.11.023. Epub 2016 Nov 15. PMID: 28017210.
899	28079763	Etomidate	2017	Ye L, Xiao X, Zhu L. The Comparison of Etomidate and Propofol Anesthesia in Patients Undergoing Gastrointestinal Endoscopy: A Systematic Review and Meta-Analysis. Surg Laparosc Endosc Percutan Tech. 2017 Feb;27(1):1-7. doi: 10.1097/SLE.0000000000000373. PMID: 28079763.
900	28084272	Bismuth	2017	Siram R, Botta R, Kashikunte C, Pal PK, Yadav R. Chronic encephalopathy with ataxia, myoclonus, and auditory neuropathy: A case of bismuth poisoning. Neurol India. 2017 Jan-Feb;65(1):186-187. doi: 10.4103/0028-3886.198185. PMID: 28084272.
901	28102628	Atropine	2017	Miyoshi H, Nakamura R, Yamaga A, Haraki T, Yasuda T, Hamada H, Kawamoto M. Transient symptomatic worsening by atropine in opsoclonus-myoclonus syndrome. Pediatr Int. 2017 Jan;59(1):97-98. doi: 10.1111/ped.13180. PMID: 28102628.

902	28122422	Metronidazole	2017	Lee HC, Kim YE, Ma HI. Metronidazole-Induced Craniocervical Myoclonus with Reversible Bilateral Dentate Nucleus Lesions. J Mov Disord. 2017 Jan;10(1):67-68. doi: 10.14802/jmd.16021. Epub 2017 Jan 18. PMID: 28122422; PMCID: PMC5288659.
903	28124668	Cefuroxime	2017	van Dam DG, Burgers DM, Foudraine N, Janssen PK, Neef C, le Noble JL. Treatment of cefuroxime-induced neurotoxicity with continuous venovenous haemofiltration. Neth J Med. 2017 Jan;75(1):32-34. PMID: 28124668.
904	28131212	Lamotrigine	2017	Tombini M, Pellegrino G, Assenza G, Di Lazzaro V. De novo multifocal myoclonus induced by lamotrigine in a temporal lobe epilepsy case. J Neurol Sci. 2017 Feb 15;373:31-32. doi: 10.1016/j.jns.2016.12.022. Epub 2016 Dec 15. PMID: 28131212.
905	28173638	Valproate	2017	Riker RR, Gagnon DJ, Hatton C, May T, Seder DB, Stokem K, Fraser GL. Valproate Protein Binding Is Highly Variable in ICU Patients and Not Predicted by Total Serum Concentrations: A Case Series and Literature Review. Pharmacotherapy. 2017 Apr;37(4):500-508. doi: 10.1002/phar.1912. Epub 2017 Mar 16. PMID: 28173638.
906	28189131	Oxycodone	2017	Lau F, Gardiner M. Oxycodone/naloxone: An unusual adverse drug reaction. Aust Fam Physician. 2017 Jan/Feb;46(1):42-43. PMID: 28189131.
907	28203564	Bilimbi Fruit	2017	Caetano CP, de Sá CB, Faleiros BA, Gomes MF, Pereira ER. Neurotoxicity following the Ingestion of Bilimbi Fruit (Averrhoa bilimbi) in an End-Stage Renal Disease Patient on Hemodialysis. Case Rep Nephrol Dial. 2017 Jan 20;7(1):6-12. doi: 10.1159/000454945. PMID: 28203564; PMCID: PMC5301110.
908	28223779	Etomidate	2017	Du X, Zhou C, Pan L, Li C. Effect of dexmedetomidine in preventing etomidate-induced myoclonus: a meta-analysis. Drug Des Devel Ther. 2017 Feb 8;11:365-370. doi: 10.2147/DDDT.S121979. PMID: 28223779; PMCID: PMC5308599.
909	28223849	Gabapentin	2017	Quintero GC. Review about gabapentin misuse, interactions, contraindications and side effects. J Exp Pharmacol. 2017 Feb 9;9:13-21. doi: 10.2147/JEP.S124391. PMID: 28223849; PMCID: PMC5308580.
910	28239547	Lacosamide	2016	Birnbaum D, Koubeissi M. Unmasking of myoclonus by lacosamide in generalized epilepsy. Epilepsy Behav Case Rep. 2016 Nov 16;7:28-30. doi: 10.1016/j.ebcr.2016.09.006. PMID: 28239547; PMCID: PMC5318289.
911	28284883	Etomidate	2017	Kim MG, Park SW, Kim JH, Lee J, Kae SH, Jang HJ, Koh DH, Choi MH. Etomidate versus propofol sedation for complex upper endoscopic procedures: a prospective double-blinded randomized controlled trial. Gastrointest Endosc. 2017 Sep;86(3):452-461. doi: 10.1016/j.gie.2017.02.033. Epub 2017 Mar 8. PMID: 28284883.
912	28291508	Etomidate	2017	Li X, Liu J, Zhou M, Zhou C. Parecoxib sodium pretreatment reduces myoclonus after etomidate: A prospective, double-blind, randomized clinical trial . Int J Clin Pharmacol Ther. 2017 Jul;55(7):601-605. doi: 10.5414/CP202768. PMID: 28291508.
913	28291512	Ampicillin	2017	Fisse AL, Straßburger-Krogias K, Gold R, Ellrichmann G. Recurrent trimethoprim-sulfamethoxazole-induced aseptic meningitis with associated ampicillin-induced myoclonic twitches . Int J Clin Pharmacol Ther. 2017 Jul;55(7):627-629. doi: 10.5414/CP202957. PMID: 28291512.
914	28321081	Metoclopramide	2017	Harada T, Hirosawa T, Morinaga K, Shimizu T. Metoclopramide-induced Serotonin Syndrome. Intern Med. 2017;56(6):737-739. doi: 10.2169/internalmedicine.56.7727. Epub 2017 Mar 17. PMID: 28321081; PMCID: PMC5410491.
915	28367296	Meperidine	2017	Joe S, Kim E, Park J, Lee D, Son J, Kim H. Famotidine-induced reversal of meperidine-related serotonin syndrome: a case report. Korean J Anesthesiol. 2017 Apr;70(2):221-223. doi: 10.4097/kjae.2017.70.2.221. Epub 2017 Jan 11. PMID: 28367296; PMCID: PMC5370302.
916	28386402	Morphine	2017	Woodward OB, Naraen S, Naraen A. Opioid-induced myoclonus and hyperalgesia following a short course of low-dose oral morphine. Br J Pain. 2017 Feb;11(1):32-35. doi: 10.1177/2049463716664371. Epub 2016 Aug 22. PMID: 28386402; PMCID: PMC5370622.
917	28415947	Etomidate	2017	Zhou C, Zhu Y, Liu Z, Ruan L. Effect of pretreatment with midazolam on etomidate-induced myoclonus: A meta-analysis. J Int Med Res. 2017 Apr;45(2):399-406. doi: 10.1177/0300060516682882. Epub 2017 Feb 2. PMID: 28415947; PMCID: PMC5536644.
918	28452847	Ranolazine	2018	Trehan N, Singh M, Kottam AR. Ranolazine-Associated Myoclonus. Am J Ther. 2018 Jul/Aug;25(4):e507-e508. doi: 10.1097/MJT.0000000000000598. PMID: 28452847.
919	28488910	Methadone	2017	Hoff AM, Hartwig KN, Rosielle DA. Methadone-Induced Neurotoxicity in Advanced Cancer: A Case Report. J Palliat Med. 2017 Sep;20(9):1042-1044. doi: 10.1089/jpm.2016.0502. Epub 2017 May 10. PMID: 28488910.
920	28547577	Pramipexole	2017	Cardon-Dunbar A, Robertson T, Roberts MS, Isbister GK. Pramipexole Overdose Associated with Visual Hallucinations, Agitation and Myoclonus. J Med Toxicol. 2017 Dec;13(4):343-346. doi: 10.1007/s13181-017-0615-7. Epub 2017 May 25. PMID: 28547577; PMCID: PMC5711753.
921	28577954	Nivolumab	2017	Raskin J, Masrori P, Cant A, Snoeckx A, Hiddinga B, Kohl S, Janssens A, Cras P, Van Meerbeeck JP. Recurrent dysphasia due to nivolumab-induced encephalopathy with presence of Hu autoantibody. Lung Cancer. 2017 Jul;109:74-77. doi: 10.1016/j.lungcan.2017.05.002. Epub 2017 May 10. PMID: 28577954.
922	28658112	Etomidate	2017	Liu J, Liu R, Meng C, Cai Z, Dai X, Deng C, Zhang J, Zhou H. Propofol decreases etomidate-related myoclonus in gastroscopy. Medicine (Baltimore). 2017 Jun;96(26):e7212. doi: 10.1097/MD.0000000000007212. PMID: 28658112; PMCID: PMC5500034.
923	28676198	Gabapentin	2017	Ibrahim H, Oman Z, Schuelke M, Edwards JC. Treatment of Gabapentin Toxicity With Peritoneal Dialysis: Assessment of Gabapentin Clearance. Am J Kidney Dis. 2017 Dec;70(6):878-880. doi: 10.1053/j.ajkd.2017.05.010. Epub 2017 Jul 1. PMID: 28676198.
924	28755095	Beta-lactam	2017	Deshayes S, Coquerel A, Verdon R. Neurological Adverse Effects Attributable to β-Lactam Antibiotics: A Literature Review. Drug Saf. 2017 Dec;40(12):1171-1198. doi: 10.1007/s40264-017-0578-2. PMID: 28755095.
925	28761332	Etomidate	2017	Zhu Y, Yang Y, Zhou C, Bao Z. Using dezocine to prevent etomidate-induced myoclonus: a meta-analysis of randomized trials. Drug Des Devel Ther. 2017 Jul 18;11:2163-2170. doi: 10.2147/DDDT.S137464. PMID: 28761332; PMCID: PMC5522665.
926	28784915	Escitalopram, dextromethorphan	2017	Dy P, Arcega V, Ghali W, Wolfe W. Serotonin syndrome caused by drug to drug interaction between escitalopram and dextromethorphan. BMJ Case Rep. 2017 Aug 7;2017:bcr2017221486. doi: 10.1136/bcr-2017-221486. PMID: 28784915; PMCID: PMC5747823.
927	28802899	Pentylentetrazole	2017	Amin F, Dar AH, Osama K, Khan F, Mitha R, Tharwani A, Haider G, Chand P, Arain FM. A species dependent response to the

				pro-epileptic drug pentylentetrazole in birds. Brain Res Bull. 2017 Sep;134:189-194. doi: 10.1016/j.brainresbull.2017.08.003. Epub 2017 Aug 9. PMID: 28802899.
928	28892927	Levofloxacin	2017	Kunder SK, Avinash A, Nayak V, Tilak A. A Rare Instance of Levofloxacin Induced Myoclonus. J Clin Diagn Res. 2017 Jul;11(7):FD01-FD02. doi: 10.7860/JCDR/2017/27005.10189. Epub 2017 Jul 1. PMID: 28892927; PMCID: PMC5583779.
929	28895986	Anesthesia	2017	Kösem B, Kılınç H. An unusual complication of anesthesia: Unilateral spinal myoclonus. Agri. 2017 Apr;29(2):90-91. doi: 10.5505/agri.2016.92053. PMID: 28895986.
930	28905654	Cyclosporine	2018	Kang HG, Park SK, Wang SJ, Oh SY, Ryu HU. Opsoclonus-myoclonus syndrome following long-term use of cyclosporine. Clin Toxicol (Phila). 2018 May;56(5):373-376. doi: 10.1080/15563650.2017.1375511. Epub 2017 Sep 14. PMID: 28905654.
931	28943562	Ceftriaxone	2017	Hagiya H, Miyawaki K, Yamamoto N, Yoshida H, Kitagawa A, Asaoka T, Eguchi H, Akeda Y, Tomono K. Ceftriaxone-induced Neurotoxicity in a Patient after Pancreas-Kidney Transplantation. Intern Med. 2017 Nov 15;56(22):3103-3107. doi: 10.2169/internalmedicine.8774-16. Epub 2017 Sep 25. PMID: 28943562; PMCID: PMC5725869.
932	28975050	Ciprofloxacin	2017	van Samkar A, De Kleermaeker FGCM, Te Riele MGE, Verrips A. Negative Myoclonus Induced by Ciprofloxacin. Tremor Other Hyperkinet Mov (N Y). 2017 Sep 6;7:500. doi: 10.7916/D8QC0FXT. PMID: 28975050; PMCID: PMC5623757.
933	29046518	Etomidate	2017	An X, Li C, Sahebally Z, Wen X, Zhao B, Fang X. Pretreatment with Oxycodone Simultaneously Reduces Etomidate-Induced Myoclonus and Rocuronium-Induced Withdrawal Movements During Rapid-Sequence Induction. Med Sci Monit. 2017 Oct 19;23:4989-4994. doi: 10.12659/msm.902652. PMID: 29046518; PMCID: PMC5659139.
934	29067833	Bupropion	2018	Xiang XM, Phillips DJ. Nonepileptic Myoclonus Following Bupropion Overdose. Clin Pediatr (Phila). 2018 Aug;57(9):1100-1102. doi: 10.1177/0009922817737082. Epub 2017 Oct 25. PMID: 29067833.
935	29071284	Cefepime	2017	Appa AA, Jain R, Rakita RM, Hakimian S, Pottinger PS. Characterizing Cefepime Neurotoxicity: A Systematic Review. Open Forum Infect Dis. 2017 Oct 10;4(4):ofx170. doi: 10.1093/ofid/ofx170. PMID: 29071284; PMCID: PMC5639733.
936	29095326	Bismuth	2017	Sampognaro P, Vo KT, Richie M, Blanc PD, Keenan K. Bismuth Subgallate Toxicity in the Age of Online Supplement Use. Neurologist. 2017 Nov;22(6):237-240. doi: 10.1097/NRL.0000000000000144. PMID: 29095326.
937	29111014	Gabapentin, pregabalin	2017	Kim JB, Jung JM, Park MH, Lee EJ, Kwon DY. Negative myoclonus induced by gabapentin and pregabalin: A case series and systematic literature review. J Neurol Sci. 2017 Nov 15;382:36-39. doi: 10.1016/j.jns.2017.09.019. Epub 2017 Sep 18. PMID: 29111014.
938	29137682	Cefepime	2017	Payne LE, Gagnon DJ, Riker RR, Seder DB, Glisic EK, Morris JG, Fraser GL. Cefepime-induced neurotoxicity: a systematic review. Crit Care. 2017 Nov 14;21(1):276. doi: 10.1186/s13054-017-1856-1. PMID: 29137682; PMCID: PMC5686900.
939	29203576	Etomidate	2018	McGrath M, Ma C, Raines DE. Dimethoxy-etomidate: A Nonhypnotic Etomidate Analog that Potently Inhibits Steroidogenesis. J Pharmacol Exp Ther. 2018 Feb;364(2):229-237. doi: 10.1124/jpet.117.245332. Epub 2017 Dec 4. PMID: 29203576; PMCID: PMC5783534.
940	29225828	Cefepime	2017	Guzman-Limon M, Amatya S, Samuels J, Swinford R, Bhatnagar S, Samuel J. Cefepime-induced neurotoxicity in a pediatric patient on chronic hemodialysis: a case report. Clin Case Rep. 2017 Oct 9;5(12):1931-1933. doi: 10.1002/ccr3.1217. PMID: 29225828; PMCID: PMC5715434.
941	29260037	Cefepime	2017	Isitan C, Ferree A, Hohler AD. Cefepime induced neurotoxicity: A case series and review of the literature. eNeurologicalSci. 2017 Aug 4;8:40-43. doi: 10.1016/j.ensci.2017.08.001. PMID: 29260037; PMCID: PMC5730896.
942	29298279	Lithium	2017	Stetkarova I, Bocek V, Gismatullina A, Svobodova Z, Peisker T. Severe chronic lithium intoxication in patient treated for bipolar disorder. Neuro Endocrinol Lett. 2017 Dec;38(6):397-400. PMID: 29298279.
943	29360288	Clozapine	2018	McCollum B, Barclay J, de Leon J. Unexpected Falls During Clozapine Treatment Explained by Myoclonus. Prim Care Companion CNS Disord. 2018 Jan 18;20(1):17l02151. doi: 10.4088/PCC.17l02151. PMID: 29360288.
944	29364803	Lead	2017	Cury RG, Marin JH, Contreras Lopez WO. Lead Poisoning: Myoclonus Following Welding Exposure. Acta Med Port. 2017 Dec 29;30(12):889. doi: 10.20344/amp.9398. Epub 2017 Dec 29. PMID: 29364803.
945	29367224	Lithium	2018	Orleans RA, Dubin MJ, Kast KA. The effect of a therapeutic lithium level on a stroke-related cerebellar tremor. BMJ Case Rep. 2018 Jan 24;2018:bcr2017222920. doi: 10.1136/bcr-2017-222920. PMID: 29367224; PMCID: PMC5786966.
946	29428303	Morphine, bupivacaine	2018	McFadzean WJ, Holopherne-Doran D. Myoclonus and hypersensitivity of the tail following intrathecal administration of morphine and bupivacaine in a cat. Vet Anaesth Analg. 2018 Mar;45(2):238-239. doi: 10.1016/j.vaa.2017.09.038. Epub 2017 Oct 1. PMID: 29428303.
947	29491517	Etomidate	2018	Gupta P, Gupta M. Comparison of different doses of intravenous lignocaine on etomidate-induced myoclonus: A prospective randomised and placebo-controlled study. Indian J Anaesth. 2018 Feb;62(2):121-126. doi: 10.4103/ija.IJA_563_17. PMID: 29491517; PMCID: PMC5827478.
948	29515422	Metronidazole	2018	Sørensen CG, Karlsson WK, Amin FM, Lindelof M. Convulsive Seizures as Presenting Symptom of Metronidazole-Induced Encephalopathy: A Case Report. Case Rep Neurol. 2018 Feb 1;10(1):34-37. doi: 10.1159/000485915. PMID: 29515422; PMCID: PMC5836251.
949	29536782	Etomidate	2018	Wang W, Lv J, Wang Q, Yang L, Yu W. Oxycodone for prevention of etomidate-induced myoclonus: a randomized double-blind controlled trial. J Int Med Res. 2018 May;46(5):1839-1845. doi: 10.1177/0300060518761788. Epub 2018 Mar 14. PMID: 29536782; PMCID: PMC5991229.
950	29542295	Memantine, bupropion	2018	Ahn BJ, Kwon KY. Elderly woman presenting with unusual propriospinal myoclonus triggered by drug-induced nausea and vomiting. Geriatr Gerontol Int. 2018 Mar;18(3):504-505. doi: 10.1111/ggi.13237. PMID: 29542295.
951	29561417	General anesthesia	2018	Lee JJ, Lim S, Lee YS, Shin HY, Baek CW, Jung YH, Woo YC, Park YH. Myoclonic movement after general anesthesia: A case report and review of the literature. Medicine (Baltimore). 2018 Mar;97(12):e0141. doi: 10.1097/MD.0000000000010141. PMID: 29561417; PMCID: PMC5895320.
952	29643630	Etomidate	2018	Dey S, Kumar M. Comparison of pretreatment with dexmedetomidine with midazolam for prevention of etomidate-induced

				myoclonus and attenuation of stress response at intubation: A randomized controlled study. J Anaesthesiol Clin Pharmacol. 2018 Jan-Mar;34(1):94-98. doi: 10.4103/joacp.JOACP_297_16. PMID: 29643630; PMCID: PMC5885457.
953	29691274	Paroxetine	2018	Correia P, Ribeiro JA, Bento C, Sales F. Negative myoclonus secondary to paroxetine intake. BMJ Case Rep. 2018 Apr 24;2018:bcr2018224586. doi: 10.1136/bcr-2018-224586. PMID: 29691274; PMCID: PMC5926597.
954	29721398	Ceftazidime	2018	Ong CY, Qin Y. Myoclonus from Antibiotic Therapy (Ceftazidime-induced Neurotoxicity): A Case Report and Review. Cureus. 2018 Mar 1;10(3):e2250. doi: 10.7759/cureus.2250. PMID: 29721398; PMCID: PMC5929940.
955	29742520	Ipilimumab	2018	Maller B, Peguero E, Tanvetyanon T. Ipilimumab/Nivolumab-related Opsoclonus-Myoclonus-Ataxia Syndrome Variant in a Patient with Malignant Pleural Mesothelioma. J Immunother. 2018 Nov/Dec;41(9):411-412. doi: 10.1097/CJI.0000000000000228. PMID: 29742520.
956	29768328	Etomidate	2018	Lee JM, Min G, Lee JM, Kim SH, Choi HS, Kim ES, Keum B, Jeen YT, Chun HJ, Lee HS, Kim CD, Park JJ, Lee BJ, Choi SJ, Kim W. Efficacy and safety of etomidate-midazolam for screening colonoscopy in the elderly: A prospective double-blinded randomized controlled study. Medicine (Baltimore). 2018 May;97(20):e10635. doi: 10.1097/MD.0000000000010635. PMID: 29768328; PMCID: PMC5976307.
957	29879048	Etomidate	2018	Lv Y, He H, Xie J, Jin W, Shou C, Pan Y, Wang L, Mo Y, Dai Q, Geng W, Wang J. Effects of transcutaneous acupoint electrical stimulation combined with low-dose sufentanil pretreatment on the incidence and severity of etomidate-induced myoclonus: A randomized controlled trial. Medicine (Baltimore). 2018 Jun;97(23):e10969. doi: 10.1097/MD.0000000000010969. PMID: 29879048; PMCID: PMC5999512.
958	29925727	Amantadine	2018	Ono H, Okamura M, Fukushima A. [Similarity of Clinically Significant Neuropsychiatric Adverse Reactions Listed in Package Inserts between the Anti-influenza Drugs Oseltamivir and Amantadine (Possibility Attributable to Common Pharmacological Effects)]. Yakugaku Zasshi. 2018 Sep 1;138(9):1201-1215. Japanese. doi: 10.1248/yakushi.18-00022. Epub 2018 Jun 20. PMID: 29925727.
959	29927775	Methylphenidate	2019	Naguy A, Alamiri B, El-Sori DS, Khraibut B. Methylphenidate-Associated Onychotillomania, Myoclonus, and Enuresis Reversed by Switching to Atomoxetine. Am J Ther. 2019 Sep/Oct;26(5):e614-e616. doi: 10.1097/MJT.0000000000000812. PMID: 29927775.
960	29928219	Fentanyl	2018	Almedallah DK, Alshamlan DY, Shariff EM. Acute Opioid-Induced Myoclonic Reaction after Use of Fentanyl as an Anesthetic Drug for an Emergency Cesarean Section. Case Rep Neurol. 2018 May 30;10(2):130-134. doi: 10.1159/000486891. PMID: 29928219; PMCID: PMC6006639.
961	29939205	Donepezil	2018	Whateley JM, Huffman AJ, Henderson EJ. Acute inability to mobilise resulting from probable donepezil-induced myoclonus. Age Ageing. 2018 Nov 1;47(6):907-908. doi: 10.1093/ageing/afy093. PMID: 29939205.
962	30004301	Hydromorphone	2018	Martin EJ, Vaughan CL, Atayee R, Hirst JM, O'Donnell K, Edmonds KP. Hydromorphone-induced chorea as an atypical presentation of opioid neurotoxicity: A case report and review of the literature. Palliat Med. 2018 Oct;32(9):1529-1532. doi: 10.1177/0269216318786861. Epub 2018 Jul 13. PMID: 30004301.
963	30054843	Etomidate	2019	Han SJ, Lee TH, Yang JK, Cho YS, Jung Y, Chung IK, Park SH, Park S, Kim SJ. Etomidate Sedation for Advanced Endoscopic Procedures. Dig Dis Sci. 2019 Jan;64(1):144-151. doi: 10.1007/s10620-018-5220-3. Epub 2018 Jul 27. PMID: 30054843.
964	30071713	Etomidate	2018	Mullick P, Talwar V, Aggarwal S, Prakash S, Pawar M. Comparison of priming versus slow injection for reducing etomidate-induced myoclonus: a randomized controlled study. Korean J Anesthesiol. 2018 Aug;71(4):305-310. doi: 10.4097/kja.d.18.27168. Epub 2018 Jul 30. PMID: 30071713; PMCID: PMC6078874.
965	30104829	Etomidate	2018	Gupta M, Gupta P. Nalbuphine pretreatment for prevention of etomidate induced myoclonus: A prospective, randomized and double-blind study. J Anaesthesiol Clin Pharmacol. 2018 Apr-Jun;34(2):200-204. doi: 10.4103/joacp.JOACP_210_16. PMID: 30104829; PMCID: PMC6066906.
966	30148931	Bupropion	2018	Cartier R L, Romero O C, Pérez P G. Supraversión de la mirada en intoxicación mortal por bupropión [Sustained upgaze in a patient who ingested high doses of bupropion. Report of one case]. Rev Med Chil. 2018 May;146(5):665-669. Spanish. doi: 10.4067/s0034-98872018000500665. PMID: 30148931.
967	30175627	Etomidate	2018	Choi GJ, Kang H, Baek CW, Jung YH, Ko JS. Etomidate versus propofol sedation for electrical external cardioversion: a meta-analysis. Curr Med Res Opin. 2018 Nov;34(11):2023-2029. doi: 10.1080/03007995.2018.1519501. Epub 2018 Sep 20. PMID: 30175627.
968	30224173	Aldicarb	2019	Poyato Borrego M, Rufo Tejeiro O, Villarrasa Clemente FM. Generalized myoclonus after ingestion of aldicarb as suicide attempt. Med Clin (Barc). 2019 Apr 18;152(8):329-330. English, Spanish. doi: 10.1016/j.medcli.2018.07.005. Epub 2018 Sep 14. PMID: 30224173.
969	30279807	Contrast induced	2017	Abela L, Magri Gatt K, Farrugia J, Mallia M. Contrast induced spinal myoclonus after percutaneous coronary intervention. J Cardiol Cases. 2017 Jul 21;16(3):97-100. doi: 10.1016/j.jccase.2017.05.012. PMID: 30279807; PMCID: PMC6149275.
970	30323563	Etomidate	2018	Lang B, Zhang L, Yang C, Lin Y, Zhang W, Li F. Pretreatment with lidocaine reduces both incidence and severity of etomidate-induced myoclonus: a meta-analysis of randomized controlled trials. Drug Des Devel Ther. 2018 Oct 4;12:3311-3319. doi: 10.2147/DDDT.S174057. PMID: 30323563; PMCID: PMC6174893.
971	30335235	Salbutamol	2018	Montoya-Giraldo MA, Montoya DV, Atehortúa DA, Buendía JA, Zuluaga AF. Myoclonus induced by salbutamol: A case report. Biomedica. 2018 Sep 1;38(3):303-307. doi: 10.7705/biomedica.v38i3.3813. PMID: 30335235.
972	30336409	Spinal anesthesia	2019	Chen RW, Aggarwal SK, Pisano DV. Autonomic instability triggered by spinal anesthesia. J Clin Anesth. 2019 Feb;52:122. doi: 10.1016/j.jclinane.2018.09.020. Epub 2018 Oct 16. PMID: 30336409.
973	30381161	Gabapentin, pregabalin	2019	Desai A, Kherallah Y, Szabo C, Marawar R. Gabapentin or pregabalin induced myoclonus: A case series and literature review. J Clin Neurosci. 2019 Mar;61:225-234. doi: 10.1016/j.jocn.2018.09.019. Epub 2018 Oct 28. PMID: 30381161.
974	30381307	Linezolid	2018	Khoury A, Runnstrom M, Ebied A, Penny ES. Linezolid-associated serotonin toxicity after escitalopram discontinuation: concomitant drug considerations. BMJ Case Rep. 2018 Oct 30;2018:bcr2018226597. doi: 10.1136/bcr-2018-226597. PMID: 30381307; PMCID: PMC6214389.
975	30412900	Cefepime	2019	Lizarraga KJ, Heros DO, Adams D, Lang AE, Kanner AM. Opsoclonus-myoclonus-encephalopathy induced by cefepime. J Neurol Sci. 2019 Jan 15;396:33-35. doi: 10.1016/j.jns.2018.10.028. Epub 2018 Nov 1. PMID: 30412900.

976	30415896	Fentanyl analog	2019	Nellhaus EM, Murray S, Hansen Z, Loudin S, Davies TH. Novel Withdrawal Symptoms of a Neonate Prenatally Exposed to a Fentanyl Analog. J Pediatr Health Care. 2019 Jan;33(1):102-106. doi: 10.1016/j.pedhc.2018.08.014. Epub 2018 Nov 8. PMID: 30415896.
977	30497306	Etomidate	2019	Zhu Y, Zhou C, He Q. Butorphanol effectively prevents etomidate-induced myoclonus: a pooled analysis of 788 patients. J Int Med Res. 2019 Jan;47(1):353-360. doi: 10.1177/0300060518801457. Epub 2018 Nov 30. PMID: 30497306; PMCID: PMC6384487.
978	30613902	Bupivacaine, dibucaine, lidocaine, prilocaine, tetracaine, levobupivacaine	2019	Shiratori T, Hotta K, Satoh M. Spinal myoclonus following neuraxial anesthesia: a literature review. J Anesth. 2019 Feb;33(1):140-147. doi: 10.1007/s00540-018-02607-z. Epub 2019 Jan 6. PMID: 30613902.
979	30625903	Etomidate	2009	Lee SW, Gill HJ, Park SC, Kim JY, Kim JH, Lee JY, Yang HJ, Kim MK. The effect of remifentanil for reducing myoclonus during induction of anesthesia with etomidate. Korean J Anesthesiol. 2009 Oct;57(4):438-443. doi: 10.4097/kjae.2009.57.4.438. PMID: 30625903.
980	30839320	Gabapentin	2019	Yeddi A, Adam O, Khalid M, Benjaram S, Abu-Heija A, Abdallah MA, Shah P. Myoclonus and Altered Mental Status Induced by Single Dose of Gabapentin in a Patient With End-Stage Renal Disease: A Case Report and Literature Review. Am J Ther. 2019 Nov/Dec;26(6):e768-e770. doi: 10.1097/MJT.0000000000000942. PMID: 30839320.
981	30844852	Escitalopram, clomipramine	2019	Suzuki A, Otani K. Serotonin Syndrome After an Alcohol Intake in a Patient Treated With Escitalopram and Clomipramine. Clin Neuropharmacol. 2019 May/Jun;42(3):103-104. doi: 10.1097/WNF.0000000000000331. PMID: 30844852.
982	30925500	Clozapine	2019	Chochol MD, Kataria L, O'Rourke MC, Lamotte G. Clozapine-Associated Myoclonus and Stuttering Secondary to Smoking Cessation and Drug Interaction: A Case Report. J Clin Psychopharmacol. 2019 May/Jun;39(3):275-277. doi: 10.1097/JCP.0000000000001032. PMID: 30925500.
983	31001027	Cefepime	2019	Khoo CS, Tee TY, Tan HJ, Ali RA. A Treatable Encephalopathy in a Peritoneal Dialysis Patient - Cefepime-Induced Encephalopathy. J Neurosci Rural Pract. 2019 Apr-Jun;10(2):324-326. doi: 10.4103/jnrp.jnrp_315_18. PMID: 31001027; PMCID: PMC6454930.
984	31005049	Levetiracetam	2019	Wang D, Zhou Q, Ren L, Lin Y, Gao L, Du J, Wang Y. Levetiracetam-induced a new seizure type in a girl with a novel SV2A gene mutation. Clin Neurol Neurosurg. 2019 Jun;181:64-66. doi: 10.1016/j.clineuro.2019.03.020. Epub 2019 Mar 28. PMID: 31005049.
985	31031491	Etomidate	2019	Bisht M, Pokhriyal AS, Khurana G, Sharma JP. Effect of Fentanyl and Nalbuphine for Prevention of Etomidate-Induced Myoclonus. Anesth Essays Res. 2019 Jan-Mar;13(1):119-125. doi: 10.4103/aer.AER_188_18. PMID: 31031491; PMCID: PMC6444973.
986	31037076	Valproate	2019	Nasr Esfahani P, Nasiri J, Badihian S, Yaghini O. Short-Term Side Effects of Low Dose Valproate Monotherapy in Epileptic Children: A Prospective Study. Iran J Child Neurol. 2019 Spring;13(2):37-46. PMID: 31037076; PMCID: PMC6451855.
987	31045772	Morphine	2019	Guo X, Li Y, Yang Y, Zhao Y, Guo J, Zhang Y, Peng Z, Feng Z. Intrathecal morphine combined with ropivacaine induces spinal myoclonus in cancer patients with an implanted intrathecal drug delivery system: Three case reports. Medicine (Baltimore). 2019 May;98(18):e15330. doi: 10.1097/MD.0000000000015330. PMID: 31045772; PMCID: PMC6504238.
988	31096522	Etomidate	2019	Chen L, Liang X, Tan X, Wen H, Jiang J, Li Y. Safety and efficacy of combined use of propofol and etomidate for sedation during gastroscopy: Systematic review and meta-analysis. Medicine (Baltimore). 2019 May;98(20):e15712. doi: 10.1097/MD.0000000000015712. PMID: 31096522; PMCID: PMC6531275.
989	31114161	Etomidate	2019	Hua J, Miao S, Shi M, Tu Q, Wang X, Liu S, Wang G, Gan J. Effect of butorphanol on etomidate-induced myoclonus: a systematic review and meta-analysis. Drug Des Devel Ther. 2019 Apr 16;13:1213-1220. doi: 10.2147/DDDT.S191982. PMID: 31114161; PMCID: PMC6489683.
990	31143451	Lithium	2019	Sarrigiannis PG, Zis P, Unwin ZC, Blackburn DJ, Hoggard N, Zhao Y, Billings SA, Khan AA, Yianni J, Hadjivassiliou M. Tremor after long term lithium treatment; is it cortical myoclonus? Cerebellum Ataxias. 2019 May 22;6:5. doi: 10.1186/s40673-019-0100-y. PMID: 31143451; PMCID: PMC6532190.
991	31190739	Etomidate	2019	Lang B, Zhang L, Li F, Lin Y, Zhang W, Yang C. Comparison of the efficacy and safety of remifentanil versus different pharmacological approaches on prevention of etomidate-induced myoclonus: a meta-analysis of randomized controlled trials. Drug Des Devel Ther. 2019 May 9;13:1593-1607. doi: 10.2147/DDDT.S200200. PMID: 31190739; PMCID: PMC6512956.
992	31201049	Cephalosporin	2019	Triplett JD, Lawn ND, Chan J, Dunne JW. Cephalosporin-related neurotoxicity: Metabolic encephalopathy or non-convulsive status epilepticus? J Clin Neurosci. 2019 Sep;67:163-166. doi: 10.1016/j.jocn.2019.05.035. Epub 2019 Jun 11. PMID: 31201049.
993	31205109	Gabapentin	2019	Hui CH, Leung JK, Chang RS, Shea YF. Reversible dysphagia due to gabapentin-induced jaw myoclonus. Chin Med J (Engl). 2019 Jun 20;132(12):1485-1486. doi: 10.1097/CM9.0000000000000271. PMID: 31205109; PMCID: PMC6629332.
994	31212864	Levofloxacin	2019	Nishikubo M, Kanamori M, Nishioka H. Levofloxacin-Associated Neurotoxicity in a Patient with a High Concentration of Levofloxacin in the Blood and Cerebrospinal Fluid. Antibiotics (Basel). 2019 Jun 12;8(2):78. doi: 10.3390/antibiotics8020078. PMID: 31212864; PMCID: PMC6627500.
995	31239638	Etomidate	2019	Miao S, Zou L, Wang G, Wang X, Liu S, Shi M. Effect of dexmedetomidine on etomidate-induced myoclonus: a randomized, double-blind controlled trial. Drug Des Devel Ther. 2019 May 27;13:1803-1808. doi: 10.2147/DDDT.S194456. PMID: 31239638; PMCID: PMC6554000.
996	31333113	Opioid	2019	Mercadante S. Opioid Analgesics Adverse Effects: The Other Side of the Coin. Curr Pharm Des. 2019;25(30):3197-3202. doi: 10.2174/1381612825666190717152226. PMID: 31333113.
997	31363439	Gabapentin	2019	Hampton Z, Shahrestani N, Little A. Gabapentin-induced Facial Myoclonus in the Setting of Acute on Chronic Kidney Disease. Cureus. 2019 May 25;11(5):e4758. doi: 10.7759/cureus.4758. PMID: 31363439; PMCID: PMC6663113.
998	31364825	Ondansetron	2019	Kolli V, Addula M. Ondansetron-Induced Myoclonus With Escitalopram and HAART: Role of Drug Interactions. Prim Care Companion CNS Disord. 2019 Jul 25;21(4):18l02364. doi: 10.4088/PCC.18l02364. PMID: 31364825.
999	31413902	Carbon dioxide	2019	Onder H. A Case of Severe Myoclonus due to Carbon Dioxide Retention in the Setting of Chronic Obstructive Pulmonary Disease Exacerbation. Tremor Other Hyperkinet Mov (N Y). 2019 Jun 21;9. doi: 10.7916/d8-85y8-rr41. PMID: 31413902; PMCID: PMC6692766.

1000	31464594	Bismuth	2019	Borbinha C, Serrazina F, Salavisa M, Viana-Baptista M. Bismuth encephalopathy- a rare complication of long-standing use of bismuth subsalicylate. BMC Neurol. 2019 Aug 29;19(1):212. doi: 10.1186/s12883-019-1437-9. PMID: 31464594; PMCID: PMC6714398.
1001	31618336	Pentylenetetrazol	2019	Kilinc E, Gunes H. Modulatory effects of neuropeptides on pentylenetetrazol-induced epileptic seizures and neuroinflammation in rats. Rev Assoc Med Bras (1992). 2019 Oct 10;65(9):1188-1192. doi: 10.1590/1806-9282.65.9.1188. PMID: 31618336.
1002	31620602	Pregabalin	2018	Park KD, Kim MK, Lee SJ. Negative myoclonus associated with pregabalin. Yeungnam Univ J Med. 2018 Dec;35(2):240-243. doi: 10.12701/yujm.2018.35.2.240. Epub 2018 Dec 31. PMID: 31620602; PMCID: PMC6784710.
1003	31738190	Clozapine	2020	Uzun Ö, Bolu A, Taşçi AB, Oğur B. Knee Buckling (Negative Myoclonus) Associated With Clozapine: Reports on 3 Cases. Clin Neuropharmacol. 2020 Jan/Feb;43(1):26-27. doi: 10.1097/WNF.0000000000000370. PMID: 31738190.
1004	32123760	Metoclopramide	2020	Ribeiro L, Monteiro A. Intravenous metoclopramide induced generalized multifocal myoclonus. eNeurologicalSci. 2020 Feb 21;18:100225. doi: 10.1016/j.ensci.2020.100225. PMID: 32123760; PMCID: PMC7037577.
1005	32147665	Cefepime	2020	Cunningham JM, Sachs KV, Allyn R. Cefepime-Induced Neurotoxicity Presenting with Nonconvulsive Status Epilepticus Admitted as a Stroke Alert. Am J Case Rep. 2020 Mar 9;21:e921643. doi: 10.12659/AJCR.921643. PMID: 32147665; PMCID: PMC7081951.
1006	32149815	Dexamethasone	2020	Boudier-Revéret M, Chang MC. Segmental Spinal Myoclonus After a Cervical Transforaminal Epidural Steroid Injection. Am J Phys Med Rehabil. 2020 Nov;99(11):e128-e130. doi: 10.1097/PHM.0000000000001414. PMID: 32149815.
1007	32191616	Buspirone	2020	Rissardo JP, Caprara ALF. Buspirone-associated Movement Disorder: A Literature Review. Prague Med Rep. 2020;121(1):5-24. doi: 10.14712/23362936.2020.1. PMID: 32191616.
1008	32217863	Milnacipran, perospirone	2020	Ishida T, Uchida H, Kaneko S, Sugiyama K, Hamabe Y, Mimura M, Suzuki T. Life-Threatening Serotonin Syndrome Precipitated by Discontinuation of Serotonin-Dopamine Antagonist in the Presence of Serotonergic Agents: A Case Report. Clin Neuropharmacol. 2020 May/Jun;43(3):81-83. doi: 10.1097/WNF.0000000000000385. PMID: 32217863.
1009	32280614	Etomidate	2019	Nazemroaya B, Mousavi SM. Comparison of Premedication with Low-Dose Midazolam Versus Etomidate for Reduction of Etomidate-Induced Myoclonus During General Anesthesia for Electroconvulsive Therapy: A Randomized Clinical Trial. Anesth Pain Med. 2019 Dec 23;9(6):e94388. doi: 10.5812/aapm.94388. PMID: 32280614; PMCID: PMC7118685.
1010	32321201	Tramadol	2020	Bae SY, Lee SJ. Negative myoclonus associated with tramadol use. Yeungnam Univ J Med. 2020 Oct;37(4):329-331. doi: 10.12701/yujm.2020.00108. Epub 2020 Apr 23. PMID: 32321201; PMCID: PMC7606963.
1011	32384309	Carbamazepine, oxcarbazepine	2020	Rissardo JP, Caprara ALF. Carbamazepine-, Oxcarbazepine-, Eslicarbazepine-Associated Movement Disorder: A Literature Review. Clin Neuropharmacol. 2020 May/Jun;43(3):66-80. doi: 10.1097/WNF.0000000000000387. PMID: 32384309.
1012	32388939	Propofol	2021	Kumar A, Kumar A, Kumar N, Kumar A. Intraoperative refractory status epilepticus caused by propofol -a case report. Korean J Anesthesiol. 2021 Feb;74(1):70-72. doi: 10.4097/kja.20162. Epub 2020 May 11. PMID: 32388939; PMCID: PMC7862937.
1013	32412333	Lamotrigine	2020	Hagley SP, Epstein SE, Stern JA, Poppenga R. Lamotrigine Toxicosis Treated with Intravenous Lipid Emulsion Therapy in a Dog. J Am Anim Hosp Assoc. 2020 Jul/Aug;56(4):226-230. doi: 10.5326/JAAHA-MS-6815. Epub 2020 May 15. PMID: 32412333.
1014	32419008	Amitriptyline	2020	Rissardo JP, Caprara ALF. The Link Between Amitriptyline and Movement Disorders: Clinical Profile and Outcome. Ann Acad Med Singap. 2020 Apr;49(4):236-251. PMID: 32419008.
1015	32529182	Amoxicillin-clavulanic acid	2020	Viloria-Alebesque A, Povar-Echeverría M, Bruscas-Alijarde MJ, Gracia-Gutiérrez A, Royo-Trallero L, Al-Cheikh-Felices P. Myoclonus induced by amoxicillin-clavulanic acid. Epilepsy Behav Rep. 2020 Apr 30;14:100367. doi: 10.1016/j.ebr.2020.100367. PMID: 32529182; PMCID: PMC7283096.
1016	32541330	Topiramate	2020	Rissardo JP, Caprara ALF. Topiramate-Associated Movement Disorder: Case Series and Literature Review. Clin Neuropharmacol. 2020 Jul/Aug;43(4):116-120. doi: 10.1097/WNF.0000000000000395. PMID: 32541330.
1017	32546134	Antidepressants	2020	Revet A, Montastruc F, Roussin A, Raynaud JP, Lapeyre-Mestre M, Nguyen TTH. Antidepressants and movement disorders: a postmarketing study in the world pharmacovigilance database. BMC Psychiatry. 2020 Jun 16;20(1):308. doi: 10.1186/s12888-020-02711-z. PMID: 32546134; PMCID: PMC7298955.
1018	32569451	Ondansetron	2020	Naguy A. Ondansetron-Induced Myoclonus With Escitalopram and Highly Active Antiretroviral Therapy: A Closer Look at 5-HT3 Receptors. Prim Care Companion CNS Disord. 2020 Jun 18;22(3):19l02524. doi: 10.4088/PCC.19l02524. PMID: 32569451.
1019	32606557	Levofloxacin	2020	Reddy VASK, Mittal GK, Sekhar S, Singhdev J, Mishra R. Levofloxacin-Induced Myoclonus and Encephalopathy. Ann Indian Acad Neurol. 2020 May-Jun;23(3):405-407. doi: 10.4103/aian.AIAN_429_19. Epub 2020 Jun 10. PMID: 32606557; PMCID: PMC7313561.
1020	32671642	Haloperidol	2021	Remelli F, Bugada M, Matteucci G, Brunori M, Gianotti G, Zurlo A, Volpato S. An unwanted reaction by the use of Haloperidol in hyperkinetic delirium. Aging Clin Exp Res. 2021 May;33(5):1409-1411. doi: 10.1007/s40520-020-01649-2. Epub 2020 Jul 15. PMID: 32671642; PMCID: PMC8081681.
1021	32674858	Duloxetine	2021	Trigo López J, Martínez Pías E, Carrancho García A, Pedraza Hueso MI. Opsoclonus-myoclonus syndrome secondary to duloxetine poisoning. Neurologia (Engl Ed). 2021 Apr;36(3):250-252. English, Spanish. doi: 10.1016/j.nrl.2020.05.009. Epub 2020 Jul 14. PMID: 32674858.
1022	32699729	Cefepime	2020	Khorasani-Zadeh A, Greca I, Gada K. Cefepime-Induced Seizures: The Overlooked Outpatient Adverse Reaction. Cureus. 2020 Jul 19;12(7):e9268. doi: 10.7759/cureus.9268. PMID: 32699729; PMCID: PMC7372227.
1023	32719978	Pentylenetetrazol	2020	Durankuş F, Şenkal E, Sünnetçi E, Albayrak Y, Beyazyüz M, Atasoy Ö, Erbaş O. Beneficial Effects of Ibuprofen on Pentylenetetrazol-induced Convulsion. Neurochem Res. 2020 Oct;45(10):2409-2416. doi: 10.1007/s11064-020-03101-3. Epub 2020 Jul 27. PMID: 32719978.
1024	32789224	Amantadine	2017	Kunieda K, Shigematsu T, Fujishima I. Case Reports Describing Amantadine Intoxication in a Rehabilitation Hospital. Prog Rehabil Med. 2017 Dec 27;2:20170017. doi: 10.2490/prm.20170017. PMID: 32789224; PMCID: PMC7365224.
1025	32817824	Amiodarone	2020	Celli D, Marquez A, Byer M, Colombo R. Levetiracetam for the treatment of myoclonic neurotoxicity induced by amiodarone. HeartRhythm Case Rep. 2020 May 20;6(8):488-490. doi: 10.1016/j.hrcr.2020.04.010. PMID: 32817824; PMCID: PMC7424226.

1026	33019383	Midodrine	2020	Ye X, Ling B, Wu J, Wu S, Ren Y, Zhang H, Song F, Xuan Z, Chen M. Case report: severe myoclonus associated with oral midodrine treatment for hypotension. Medicine (Baltimore). 2020 Oct 2;99(40):e21533. doi: 10.1097/MD.0000000000021533. PMID: 33019383; PMCID: PMC7535667.
1027	33033779	Pregabalin	2020	Rissardo JP, Caprara ALF. Pregabalin-associated movement disorders: A literature review. Brain Circ. 2020 Jun 26;6(2):96-106. doi: 10.4103/bc.bc_57_19. PMID: 33033779; PMCID: PMC7511912.
1028	33157963	Etomidate	2020	Hao L, Hu X, Zhu B, Li W, Huang X, Kang F. Clinical observation of the combined use of propofol and etomidate in painless gastroscopy. Medicine (Baltimore). 2020 Nov 6;99(45):e23061. doi: 10.1097/MD.0000000000023061. PMID: 33157963; PMCID: PMC7647540.
1029	33173368	Immune Checkpoint Inhibitors	2020	Yu CW, Yau M, Mezey N, Joarder I, Micieli JA. Neuro-ophthalmic Complications of Immune Checkpoint Inhibitors: A Systematic Review. Eye Brain. 2020 Nov 3;12:139-167. doi: 10.2147/EB.S277760. PMID: 33173368; PMCID: PMC7648547.
1030	33178486	NA	2020	Chouksey A, Pandey S. Clinical Spectrum of Drug-Induced Movement Disorders: A Study of 97 Patients. Tremor Other Hyperkinet Mov (N Y). 2020 Oct 26;10:48. doi: 10.5334/tohm.554. PMID: 33178486; PMCID: PMC7597587.
1031	33480615	Gabapentin	2021	Brown A, Esechie A, Gogia B, Shanina E. Gabapentin-Induced Myokymia: A Case Report. Clin Neuropharmacol. 2021 Mar-Apr 01;44(2):75-76. doi: 10.1097/WNF.0000000000000434. PMID: 33480615.
1032	33565094	Etomidate	2021	Jones TL, Calbay R, da Cunha AF, Hofmeister EH. Descriptive assessment of adverse events associated with midazolam-etomidate versus saline-etomidate in healthy hydromorphone premedicated dogs. J Small Anim Pract. 2021 Jun;62(6):437-441. doi: 10.1111/jsap.13304. Epub 2021 Feb 10. PMID: 33565094.
1033	33614301	Cefepime	2021	Oyenuga M, Oyenuga A, Rauf A, Balogun O, Singh N. New Onset Non-Convulsive Status Epilepticus Despite Cefepime Renal Dose Adjustment. Cureus. 2021 Jan 13;13(1):e12689. doi: 10.7759/cureus.12689. PMID: 33614301; PMCID: PMC7883526.
1034	33622074	Amantadine	2021	Poon LH, Lee AJ, Vuong M, Zuzuarregui JR. Amantadine Associated Myoclonus: Case Report and Review of the Literature. J Pharm Pract. 2021 Oct;34(5):814-817. doi: 10.1177/0897190021997003. Epub 2021 Feb 24. PMID: 33622074.
1035	33717772	Cefepime	2021	Shah S, Bland S. Cefepime-Induced Encephalopathy With Seizures in a Pediatric Patient With End-Stage Renal Disease Rapidly Reversed by High-Efficiency Hemodialysis. Cureus. 2021 Mar 12;13(3):e13842. doi: 10.7759/cureus.13842. PMID: 33717772; PMCID: PMC7954516.
1036	33728142	Cefepime	2021	Keerty D, Shareef NA, Ramsakal A, Haynes E, Syed M. Cefepime-Induced Encephalopathy. Cureus. 2021 Feb 4;13(2):e13125. doi: 10.7759/cureus.13125. PMID: 33728142; PMCID: PMC7935159.
1037	33819498	Etomidate	2022	Moningi S, Reddy GP, Nikhar SA, Chikkala R, Kulkarni DK, Ramachandran G. Comparison of the influence of low dose etomidate and propofol as priming dose on the incidence of etomidate induced myoclonus: a randomised, double-blind clinical trial. Braz J Anesthesiol. 2022 Mar-Apr;72(2):261-266. doi: 10.1016/j.bjane.2021.02.047. Epub 2021 Apr 2. PMID: 33819498; PMCID: PMC9373251.
1038	33832932	Cefepime	2021	Zimmermann P, Camenzind D, Beer JH, Tarnutzer AA. Negative myoclonus as the leading symptom in acute cefepime neurotoxicity. BMJ Case Rep. 2021 Apr 8;14(4):e239744. doi: 10.1136/bcr-2020-239744. PMID: 33832932; PMCID: PMC8039221.
1039	33838567	SSRIs	2021	Magli G, Conti M, Polano R, Luzzu G, Fadda M, Sotgiu S, Casellato S. Seizures associated with selective serotonin reuptake inhibitors: A case of pharmacologically induced epileptic myoclonia. Seizure. 2021 May;88:75-77. doi: 10.1016/j.seizure.2021.03.031. Epub 2021 Apr 1. PMID: 33838567.
1040	33867552	Morphine	2021	Fujiyama M, Lavallée J, Lewis K, Duke-Novakovski T. Myoclonus and hypersensitivity of the hind limbs and tail with urinary retention following neuraxial administration of morphine in a cat. Can Vet J. 2021 Apr;62(4):389-392. PMID: 33867552; PMCID: PMC7953927.
1041	34046142	Cobalamin	2021	Hasbaoui BE, Mebrouk N, Saghir S, Yajouri AE, Abilkassem R, Agadr A. Vitamin B12 deficiency: case report and review of literature. Pan Afr Med J. 2021 Mar 4;38:237. doi: 10.11604/pamj.2021.38.237.20967. PMID: 34046142; PMCID: PMC8140678.
1042	34049752	Indomethacin	2021	Chan TLH, Hindiyeh N. Hemicrania continua: Indomethacin induced myoclonus. Clin Neurol Neurosurg. 2021 Jul;206:106703. doi: 10.1016/j.clineuro.2021.106703. Epub 2021 May 20. PMID: 34049752.
1043	34092866	Etomidate	2020	Rajpurohit V, Chaudhary K, Kishan R, Kumari K, Sethi P, Sharma A. Bi-Spectral Index-Guided Comparison of Propofol versus Etomidate for Induction in Electroconvulsive Therapy. Anesth Essays Res. 2020 Jul-Sep;14(3):504-509. doi: 10.4103/aer.AER_92_20. Epub 2021 Mar 22. PMID: 34092866; PMCID: PMC8159030.
1044	34243055	Lamotrigine	2021	Zouari R, Bouchaala W, Nsir SB, Kamoun F, Triki C. Opsoclonus myoclonus induced by lamotrigine toxicity. Seizure. 2021 Oct;91:247-250. doi: 10.1016/j.seizure.2021.06.021. Epub 2021 Jun 29. PMID: 34243055.
1045	34307616	Clozapine	2021	Le DS, Su H, Liao ZL, Yu EY. Low-dose clozapine-related seizure: A case report and literature review. World J Clin Cases. 2021 Jul 16;9(20):5611-5620. doi: 10.12998/wjcc.v9.i20.5611. PMID: 34307616; PMCID: PMC8281419.
1046	34316630	Amantadine	2020	Dames B, Karl JA, Verhagen Metman L. High dose amantadine therapy may cause increased falling in patients with Parkinson's disease: A case report. Clin Park Relat Disord. 2020 Feb 22;3:100045. doi: 10.1016/j.prdoa.2020.100045. PMID: 34316630; PMCID: PMC8298788.
1047	34606429	Valproate	2021	Rissardo JP, Caprara ALF, Durante Í. Valproate-associated Movement Disorder: A Literature Review. Prague Med Rep. 2021;122(3):140-180. doi: 10.14712/23362936.2021.14. PMID: 34606429.
1048	34642157	Cannabidiol	2021	Zawar I, Franic L, Kotagal P, Knight EP. Exacerbation of eyelid myoclonia in patients with epilepsy and eyelid myoclonia receiving cannabidiol. Epileptic Disord. 2021 Dec 1;23(6):906-910. doi: 10.1684/epd.2021.1338. PMID: 34642157.
1049	34646604	Tramadol	2021	Wasey W, Aziz I, Saleh S, Manahil N, Wasey N. Tramadol Induced Jerks. Cureus. 2021 Aug 29;13(8):e17547. doi: 10.7759/cureus.17547. PMID: 34646604; PMCID: PMC8481130.
1050	34815949	Escitalopram, bupropion, lurasidone, lamotrigine, vortioxetine	2021	Thumtecho S, Wainipitapong S, Suteparuk S. Escitalopram, bupropion, lurasidone, lamotrigine and possible vortioxetine overdose presented with serotonin syndrome and diffuse encephalopathy: A case report. Toxicol Rep. 2021 Nov 6;8:1846-1848. doi: 10.1016/j.toxrep.2021.11.003. PMID: 34815949; PMCID: PMC8591335.

1051	34877439	Levetiracetam	2021	Bou Nasif M, Varade S, Koubeissi MZ. Multifocal myoclonus as a presentation of levetiracetam toxicity. Clin Neurophysiol Pract. 2021 Nov 7;6:281-284. doi: 10.1016/j.cnp.2021.10.004. PMID: 34877439; PMCID: PMC8632708.
1052	34975141	Acamprosate	2021	Soni PK, Singh LK, Das S, Nandan NK. Acamprosate-induced myoclonic jerks: A rare side effect. Indian J Pharmacol. 2021 Nov-Dec;53(6):511-512. doi: 10.4103/ijp.ijp_386_21. PMID: 34975141; PMCID: PMC8764974.
1053	34979637	Lamotrigine	2021	Rissardo JP, Fornari Caprara AL. Lamotrigine-Associated Movement Disorder: A Literature Review. Neurol India. 2021 Nov-Dec;69(6):1524-1538. doi: 10.4103/0028-3886.333440. PMID: 34979637.
1054	35273864	Cefepime	2022	Sharma S, Khan M, Owais M, Haider A. Cefepime-Induced Neurotoxicity in a 74-Year-Old Woman. Cureus. 2022 Feb 4;14(2):e21918. doi: 10.7759/cureus.21918. PMID: 35273864; PMCID: PMC8901163.
1055	35345760	Caffeine	2022	Ohta R, Sano C. Serotonin Syndrome Triggered by Overuse of Caffeine and Complicated With Neuroleptic Malignant Syndrome: A Case Report. Cureus. 2022 Feb 21;14(2):e22468. doi: 10.7759/cureus.22468. PMID: 35345760; PMCID: PMC8942071.
1056	35379091	Daptomycin	2021	Scolari MJ, Pellegrini D. Daptomycin associated myoclonus: A case report. Farm Hosp. 2021 Nov 23;46(1):40-42. English. PMID: 35379091.
1057	35395161	Pentylenetetrazole	2022	Khatami P, Mirazi N, Khosravi M, Bananej M. Anticonvulsant activity of oxaprozin in a rat model of pentylenetetrazole-induced seizure by targeting oxidative stress and SIRT1/PGC1α signaling. Can J Physiol Pharmacol. 2022 Jun 1;100(6):534-541. doi: 10.1139/cjpp-2021-0757. Epub 2022 Apr 8. PMID: 35395161.
1058	35559341	Etomidate	2022	Zhang KD, Wang LY, Zhang DX, Zhang ZH, Wang HL. Comparison of the Effectiveness of Various Drug Interventions to Prevent Etomidate-Induced Myoclonus: A Bayesian Network Meta-Analysis. Front Med (Lausanne). 2022 Apr 26;9:799156. doi: 10.3389/fmed.2022.799156. PMID: 35559341; PMCID: PMC9086535.
1059	35586533	Pembrolizumab	2022	Gallagher A, Murphy M, McDermott R, Alexander M, O'Dowd S. Pembrolizumab-Induced Steroid-Responsive Myoclonus. Mov Disord Clin Pract. 2022 May 3;9(4):546-550. doi: 10.1002/mdc3.13453. PMID: 35586533; PMCID: PMC9092743.
1060	35641732	Quetiapine	2022	Fioravanti V, Cavallieri F, Rossi J, Macaluso MC, Valzania F. Subcortical generalized myoclonus as a presenting symptom of quetiapine overdose. Neurol Sci. 2022 Oct;43(10):6135-6136. doi: 10.1007/s10072-022-06174-w. Epub 2022 Jun 1. PMID: 35641732; PMCID: PMC9155232.
1061	35681187	Antimicrobial	2022	Moynan D, Maqbool E, de Barra E. Antimicrobial neurotoxicity: an under-recognised cause of delirium. Acute Med. 2022;21(2):115-116. doi: 10.52964/AMJA.0908. PMID: 35681187.
1062	35687883	Quetiapine	2022	Uvais NA, Ashfaq AM. Very Low Single-Dose Quetiapine-Induced Myoclonus. Prim Care Companion CNS Disord. 2022 Jun 9;24(3):21cr02907. doi: 10.4088/PCC.21cr02907. PMID: 35687883.
1063	35804217	Levetiracetam	2022	Nonaka M, Neshige S, Maruyama H. Levetiracetam-induced myoclonus following recovery from non-convulsive status epilepticus in an elderly woman. Intern Emerg Med. 2022 Sep;17(6):1829-1830. doi: 10.1007/s11739-022-03040-7. Epub 2022 Jul 8. PMID: 35804217.
1064	35861924	Antiseizure	2022	Sáenz-Farret M, Tijssen MAJ, Eliashiv D, Fisher RS, Sethi K, Fasano A. Antiseizure Drugs and Movement Disorders. CNS Drugs. 2022 Aug;36(8):859-876. doi: 10.1007/s40263-022-00937-x. Epub 2022 Jul 21. Erratum in: CNS Drugs. 2022 Sep;36(9):1017-1018. doi: 10.1007/s40263-022-00947-9. PMID: 35861924.
1065	35909709	Lithium	2022	Rissardo JP, Caprara ALF, Durante Í, Rauber A. Lithium-associated movement disorder: A literature review. Brain Circ. 2022 Jun 30;8(2):76-86. doi: 10.4103/bc.bc_77_21. PMID: 35909709; PMCID: PMC9336594.
1066	35971666	Cefepime	2022	Maan G, Keitoku K, Kimura N, Sawada H, Pham A, Yeo J, Hagiya H, Nishimura Y. Cefepime-induced neurotoxicity: systematic review. J Antimicrob Chemother. 2022 Oct 28;77(11):2908-2921. doi: 10.1093/jac/dkac271. PMID: 35971666.
1067	35974938	Lamotrigine	2022	Daneshyar S, Ghiasian M, Moradi S, Khanlarzadeh E. Efficacy of levetiracetam, lamotrigine and sodium valproate on seizure attacks and EEG disorders in patients with juvenile myoclonic epilepsy: A double blind randomized clinical trial. Caspian J Intern Med. 2022 Summer;13(3):617-622. doi: 10.22088/cjim.13.3.617. PMID: 35974938; PMCID: PMC9348222.
1068	36072169	Quetiapine	2022	Varma S, Xavier S, Desai S, Ali S. A Case of Serotonin Syndrome Precipitated by Quetiapine in a Middle-Aged Female on Trazodone and Sertraline. Cureus. 2022 Aug 4;14(8):e27668. doi: 10.7759/cureus.27668. PMID: 36072169; PMCID: PMC9440612.
1069	36111923	Etomidate	2022	Feng Y, Liu J, Zhang WS. Etomidate-induced myoclonus correlates with the dysfunction of astrocytes and glutamate transporters in the neocortex of Sprague-Dawley rats. Eur Rev Med Pharmacol Sci. 2022 Sep;26(17):6221-6235. doi: 10.26355/eurrev_202209_29640. PMID: 36111923.
1070	36369970	Ceftazidime/ avibactam	2022	Guo X, Guo M, Li J, Cui X. Central nervous system adverse events of ceftazidime/avibactam: A retrospective study using Food and Drug Administration Adverse Event Reporting System. J Clin Pharm Ther. 2022 Dec;47(12):2369-2372. doi: 10.1111/jcpt.13796. Epub 2022 Nov 12. PMID: 36369970.
1071	36578637	Phenytoin	2022	Rissardo JP, Caprara ALF. Phenytoin-associated movement disorder: A literature review. Tzu Chi Med J. 2022 Oct 3;34(4):409-417. doi: 10.4103/tcmj.tcmj_74_22. PMID: 36578637; PMCID: PMC9791846.
1072	36601749	Aciclovir	2023	Vonberg FW, Dawson A, Scott G, Davies N. Aciclovir-induced neurotoxicity. Pract Neurol. 2023 Apr;23(2):157-159. doi: 10.1136/pn-2022-003597. Epub 2022 Dec 8. PMID: 36601749.
1073	36607803	Etomidate	2023	Feng Y, Chang P, Kang Y, Liao P, Li CY, Liu J, Zhang WS. Etomidate-Induced Myoclonus in Sprague-Dawley Rats Involves Neocortical Glutamate Accumulation and N -Methyl- d -Aspartate Receptor Activity. Anesth Analg. 2023 Jul 1;137(1):221-233. doi: 10.1213/ANE.0000000000006292. Epub 2022 Dec 1. PMID: 36607803.
1074	36609395	Organophosphate	2023	Haridas A, Ravi P. Opsoclonus-myoclonus syndrome caused by organophosphate poisoning. Pract Neurol. 2023 Jun;23(3):243-245. doi: 10.1136/pn-2022-003612. Epub 2023 Jan 6. PMID: 36609395.
1075	36686274	Dobutamine	2022	Noel E, Fayoda B, Rabbani R, Benjamin YS, Lee J, Gillespie A. Dobutamine-Induced Myoclonus in a Peritoneal Dialysis Patient: Case Report. Kidney Med. 2022 Dec 17;5(3):100591. doi: 10.1016/j.xkme.2022.100591. PMID: 36686274; PMCID: PMC9851887.
1076	36709514	Haloperidol	2023	Hayase T, Saiga H, Yamaguchi T. Haloperidol-induced myoclonus in a patient with delirium. Geriatr Gerontol Int. 2023

				Mar;23(3):243-244. doi: 10.1111/ggi.14550. Epub 2023 Jan 29. PMID: 36709514.
1077	36728323	Amiodarone	2024	Paulraj S, Ahmed J. Amiodarone Rechallenge-Associated Myoclonus. Am J Ther. 2024 Jan-Feb 01;31(1):e45-e47. doi: 10.1097/MJT.0000000000001580. Epub 2023 Jan 9. PMID: 36728323.
1078	36789096	Etomidate	2023	Hu B, Zhang M, Wu Z, Zhang X, Zou X, Tan L, Song T, Li X. Comparison of Remimazolam Tosilate and Etomidate on Hemodynamics in Cardiac Surgery: A Randomised Controlled Trial. Drug Des Devel Ther. 2023 Feb 8;17:381-388. doi: 10.2147/DDDT.S401969. PMID: 36789096; PMCID: PMC9922514.
1079	36820568	Etomidate	2023	Hong JT, Park SW. Etomidate versus propofol for sedation in gastrointestinal endoscopy: A systematic review and meta-analysis of outcomes. Medicine (Baltimore). 2023 Feb 10;102(6):e32876. doi: 10.1097/MD.0000000000032876. PMID: 36820568; PMCID: PMC9907930.
1080	36852358	Cefepime	2023	Abu-Abaa M, Bahadli D, Abdulhussein O, Abdulsahib A, Landau D. Cefepime-Induced Neurotoxicity Can Be Confused With Neuroleptic Malignant Syndrome, Catatonia and Serotonin Syndrome: A Case Report. Cureus. 2023 Jan 26;15(1):e34223. doi: 10.7759/cureus.34223. PMID: 36852358; PMCID: PMC9960373.
1081	36876671	Etomidate	2023	Feng Y, Chen XB, Zhang YL, Chang P, Zhang WS. Propofol decreased the etomidate-induced myoclonus in adult patients: a meta-analysis and systematic review. Eur Rev Med Pharmacol Sci. 2023 Feb;27(4):1322-1335. doi: 10.26355/eurrev_202302_31366. PMID: 36876671.
1082	37066709	Nitrous oxide	2023	Sepahvand M, Rashidi S, Emamikhah M, Rohani M, Yazdi N. Laughing Ceased, Nitrous Oxide-Induced Myelopathy Evolved. Can J Neurol Sci. 2023 Apr 17:1-3. doi: 10.1017/cjn.2023.44. Epub ahead of print. PMID: 37066709.
1083	37335194	Clozapine	2023	Rodin I, Evans A. Case of Clozapine-Associated Negative Myoclonus. J Clin Psychopharmacol. 2023 Jul-Aug 01;43(4):386-387. doi: 10.1097/JCP.0000000000001717. Epub 2023 Jun 19. PMID: 37335194.
1084	37367728	Fluoroquinolone	2023	Rissardo JP, Caprara ALF. Fluoroquinolone-Associated Movement Disorder: A Literature Review. Medicines (Basel). 2023 May 25;10(6):33. doi: 10.3390/medicines10060033. PMID: 37367728; PMCID: PMC10303425.
1085	37380026	Cobalamin	2023	Özyürek H, Ince H, Tasdemir HA, Aydin OF. Involuntary Movements in Cobalamin Deficiency. Klin Padiatr. 2023 Jun 28. English. doi: 10.1055/a-2085-8461. Epub ahead of print. PMID: 37380026.
1086	37400075	Bupivacaine, morphine	2023	Cenani A. A case of myoclonus in a cat after intrathecal injection of bupivacaine and morphine. J Am Vet Med Assoc. 2023 Jun 29;261(10):1555-1557. doi: 10.2460/javma.23.05.0277. PMID: 37400075.
1087	37441201	Bupropion	2023	Riaz A, Ali HT, Allahham A, Fornari Caprara AL, Rissardo JP. Bupropion-Induced Myoclonus: Case Report and Review of the Literature. Neurohospitalist. 2023 Jul;13(3):297-302. doi: 10.1177/19418744231173283. Epub 2023 Apr 25. PMID: 37441201; PMCID: PMC10334061.
1088	37463777	Tranexamic acid	2023	Costa L, Costa M, Martins J, Castro R. Polymyoclonus, ventricular fibrillation and Takotsubo after accidental spinal injection of tranexamic acid. BMJ Case Rep. 2023 Jul 18;16(7):e251814. doi: 10.1136/bcr-2022-251814. PMID: 37463777; PMCID: PMC10357813.
1089	37489424	Amantadine	2023	Rissardo JP, Fornari Caprara AL. Myoclonus Secondary to Amantadine: Case Report and Literature Review. Clin Pract. 2023 Jul 20;13(4):830-837. doi: 10.3390/clinpract13040075. PMID: 37489424; PMCID: PMC10366862.
1090	37560913	Etomidate	2024	Greenwood J, Crull A, Graves M, Ledvina M. Pharmacological interventions for reducing the incidence of myoclonus in patients receiving etomidate for induction of general anesthesia: an umbrella review. JBI Evid Synth. 2024 Jan 1;22(1):66-89. doi: 10.11124/JBIES-22-00390. PMID: 37560913.
1091	37636233	Meropenem	2023	Millar Vernetti P, Dalamo K, Khan Z, Gonzalez-Duarte A, Frucht S, Kaufmann H. Meropenem-Induced Facial Myoclonus. Mov Disord Clin Pract. 2023 Aug 24;10(Suppl 3):S21-S23. doi: 10.1002/mdc3.13777. PMID: 37636233; PMCID: PMC10448627.
1092	37655076	Etomidate	2023	Alipour M, Abdi N, Zaj P, Mashhadi L. Efficacy of Granisetron versus Sufentanil on Reducing Myoclonic Movements Following Etomidate: Double-blind, randomised clinical trial. Sultan Qaboos Univ Med J. 2023 Aug;23(3):380-386. doi: 10.18295/squmj.1.2023.009. Epub 2023 Aug 28. PMID: 37655076; PMCID: PMC10467562.
1093	37682124	Etomidate	2023	Shan G, Lu H, Dai F, Liu Y, Yin D, Cao H. Low-dose nalmefene pretreatment reduces etomidate-induced myoclonus: A randomized, double-blind controlled trial. Medicine (Baltimore). 2023 Sep 8;102(36):e35138. doi: 10.1097/MD.0000000000035138. PMID: 37682124; PMCID: PMC10489433.
1094	37688712	Opioid	2023	Mercadante S. Opioid-induced Neurotoxicity in Patients with Cancer Pain. Curr Treat Options Oncol. 2023 Oct;24(10):1367-1377. doi: 10.1007/s11864-023-01117-9. Epub 2023 Sep 9. PMID: 37688712.
1095	37719637	Baclofen	2023	Vidanapathirana MN, Palliyaguruge T, Wijewickrama E. Baclofen-induced myoclonus in patients with renal impairment: A case series. SAGE Open Med Case Rep. 2023 Sep 14;11:2050313X231200966. doi: 10.1177/2050313X231200966. PMID: 37719637; PMCID: PMC10503278.
1096	37748004	Ciprofloxacin	2023	Javed H, Ali HT, Soliman ZA, Caprara ALF, Rissardo JP. Three Cases of Myoclonus Secondary to Ciprofloxacin: "Ciproclonus". Clin Neuropharmacol. 2023 Sep-Oct 01;46(5):200-203. doi: 10.1097/WNF.0000000000000565. Epub 2023 Jul 26. PMID: 37748004.
1097	37755242	Gabapentin	2023	Rissardo JP, Medeiros Araujo de Matos U, Fornari Caprara AL. Gabapentin-Associated Movement Disorders: A Literature Review. Medicines (Basel). 2023 Sep 6;10(9):52. doi: 10.3390/medicines10090052. PMID: 37755242; PMCID: PMC10536490.
1098	37772280	Amantadine	2023	Lin I, Armengou-Garcia L, Sasikumar S, Kuhlman G, Fox SH, Lang AE, Espay AJ. Amantadine-Induced Craniofacial Myoclonus: Distinctive Iatrogenic Dysarthria in Parkinson's Disease. Mov Disord Clin Pract. 2023 Jul 14;10(9):1408-1413. doi: 10.1002/mdc3.13828. PMID: 37772280; PMCID: PMC10525052.
1099	37795503	Magnesium	2023	Ray S, Park KW. Movement Disorders and Other Neurologic Impairment Associated With Hypomagnesemia: A Systematic Review. Neurol Clin Pract. 2023 Dec;13(6):e200202. doi: 10.1212/CPJ.0000000000200202. Epub 2023 Oct 2. PMID: 37795503; PMCID: PMC10547470.
1100	37829775	Etomidate	2023	Rautela RS, Gulabani M, Kumar P, Salhotra R, Mohta M, Verma K. Comparative assessment of dexmedetomidine and butorphanol for attenuation of etomidate-induced myoclonus: A double-blind, randomised controlled study. Indian J Anaesth. 2023 Sep;67(9):815-820. doi: 10.4103/ija.ija_414_23. Epub 2023 Sep 6. PMID: 37829775; PMCID: PMC10566664.

1101	37905262	Ceftriaxone	2023	Nanjundappa A, Munankami S, Al Talib K, Gummadi J, Bandaru SK. Ceftriaxone-Induced Encephalopathy in a Patient With End-Stage Renal Disease on Hemodialysis. Cureus. 2023 Sep 29;15(9):e46226. doi: 10.7759/cureus.46226. PMID: 37905262; PMCID: PMC10613515.
1102	37917866	Donepezil	2023	Rissardo JP, Fornari Caprara AL. Action Myoclonus Secondary to Donepezil: Case Report and Literature Review. Rambam Maimonides Med J. 2023 Oct 29;14(4):e0023. doi: 10.5041/RMMJ.10510. PMID: 37917866; PMCID: PMC10619993.
1103	37937281	Propofol	2023	Chao S, Khan R, Lieberman J, Buren M. Propofol-induced myoclonus during maintenance of anaesthesia. Anaesth Rep. 2023 Nov 5;11(2):e12253. doi: 10.1002/anr3.12253. PMID: 37937281; PMCID: PMC10626004.
1104	38021980	Gabapentin	2023	Mohamed AN, Michel MA, McFarlane SI. Gabapentin-Induced Myoclonus in a Patient With Chronic Kidney Disease. Cureus. 2023 Oct 19;15(10):e47351. doi: 10.7759/cureus.47351. PMID: 38021980; PMCID: PMC10657337.
1105	38032086	Clozapine, lithium	2024	Elmarasi O, Abdelhady S, Pathare A, Mahgoub Y. Clozapine- and Lithium-Associated Myoclonus: The Kindling Effect. A Case Report and Literature Review. J Clin Psychopharmacol. 2024 Jan-Feb 01;44(1):57-58. doi: 10.1097/JCP.0000000000001777. Epub 2023 Nov 29. PMID: 38032086.
1106	38174498	Valproic Acid	2023	Finsterer J. Myoclonus is Mostly Painless but can be Induced by Valproic Acid. Neurol India. 2023 Nov-Dec;71(6):1313-1314. doi: 10.4103/0028-3886.391375. PMID: 38174498.
1107	38500249	COVID-19 vaccine	2024	Angeles GED, Dichoso LPC, Jamora RDG. COVID-19 vaccine related movement disorders: a systematic review. J Mov Disord. 2024 Mar 19. doi: 10.14802/jmd.24001. Epub ahead of print. PMID: 38500249.
1108	38549854	Dobutamine	2024	Lee AY, Barforoshi S, Singh A, Shrestha R, Ha J, Kittleson M. Dobutamine-Induced Myoclonus in a Patient With Advanced Heart Failure and Chronic Kidney Disease. JACC Case Rep. 2024 Feb 16;29(6):102255. doi: 10.1016/j.jaccas.2024.102255. PMID: 38549854; PMCID: PMC10966379.
1109	38567024	Midazolam	2024	Park S, Ibrahim M, Torres A. Persistent Paradoxical Reaction to Midazolam despite General Anesthesia with Dexmedetomidine. Case Rep Anesthesiol. 2024 Mar 26;2024:4152422. doi: 10.1155/2024/4152422. PMID: 38567024; PMCID: PMC10987248.
1110	38586704	Linezolid	2024	Ferreira Â, Sobrosa P, Costa M, Miranda I, Guerra D. Linezolid Toxicity: A Clinical Case Report. Cureus. 2024 Mar 6;16(3):e55672. doi: 10.7759/cureus.55672. PMID: 38586704; PMCID: PMC10996881.
1111	38645917	Tacrolimus	2024	Mazumder MA, Gulati S. Tacrolimus-Induced Focal Myoclonus of Unilateral Hand in a Kidney Transplant Recipient. Indian J Nephrol. 2024 Jan-Feb;34(1):93. doi: 10.4103/ijn.ijn_146_23. Epub 2023 Aug 4. PMID: 38645917; PMCID: PMC11003582.
1112	38838738	Flumazenil	2024	D'Onofrio G, Major P. Reversal of Benzodiazepine-Induced Myoclonus by Flumazenil in the NICU. Neuropediatrics. 2024 Jun 5. doi: 10.1055/a-2338-5736. Epub ahead of print. PMID: 38838738.
1113	38863974	Tranexamic acid	2024	Tian N, Sun Y, Liu Y, Jin J, Chen S, Han H, Zhang Y, Li Z. Safety assessment of tranexamic acid: real-world adverse event analysis from the FAERS database. Front Pharmacol. 2024 May 28;15:1388138. doi: 10.3389/fphar.2024.1388138. PMID: 38863974; PMCID: PMC11165083.
1114	38868692	Atomoxetine	2022	Sato F, Suzuki A, Noto K, Shirata T, Kanno M, Kobayashi R, Otani K. Serotonin syndrome induced by overdose of atomoxetine alone in a patient with attention-deficit hyperactivity disorder: A case report. PCN Rep. 2022 Sep 2;1(3):e41. doi: 10.1002/pcn5.41. PMID: 38868692; PMCID: PMC11114337.
1115	38880018	Vigabatrin	2024	Iodice A, Marchiò G, Asta F, Rocchetti K, Rosati A. Vigabatrin-associated hyperkinetic movements in two children with epileptic spasms: Case reports and video phenomenology description. Seizure. 2024 Jun 4;120:12-14. doi: 10.1016/j.seizure.2024.06.003. Epub ahead of print. PMID: 38880018.

Table S2. Anesthetic-Induced Myoclonus.

Subclass	Drug	Reference
Anesthetic gases	Nitrous oxide	Wu et al. (2007) [17]
Volatile liquids	Enflurane	Ng et al. (1980) [15]
	Isoflurane	Harrison et al. (1986) [21]
	Sevoflurane	Conreux et al. (2001) [22]
Intravenous anesthetics	Phenols (propofol)	Tam et al. (2009) [18]
	Benzodiazepine (midazolam)	Magny et al. (1994) [23]
	Opioids (fentanyl)	Almedallah et al. (2018) [24]
	Arylcyclohexylamines (ketamine)	Boscan et al. (2005), animal models [25]
	Etomidate	Laughlin et al. (1985) [16]
Local anesthetics	Bupivacaine	Lee et al. (2010) [26]
	Dibucaine	Watanabe et al. (1987) [27]
	Lidocaine (lignocaine)	Nadkarni et al. (1982) [28]
	Prilocaine	Fores Novales et al. (2009) [29]
	Tetracaine	Fox et al. (1979) [20]
	Levobupivacaine	Kang et al. (2016) [30]

Table S3. Antibiotic-Induced Myoclonus.

Subclass	Antibiotic	References
Penicillins	Penicillin G or oxacillin	Kurtzman et al. (1970) [50]; Sackellares et al.(1979) [56]
	Amoxicillin-clavulanic acid	Viloria-Alebesque et al. (2020) [57]
	Piperacillin-tazobactam	Park-Matsumoto et al. (1996) [54]
Cephalosporins	Cefuroxime (2nd generation)	Herishanu et al. (1998) [58]
	Cefmetazole (2nd generation)	Uchihara et al. (1988) [59]
	Ceftriaxone (3rd generation)	Hagiya et al. (2017) [60]
	Ceftazidime (3rd generation)	Chan et al. (2006) [61]
	Moxalactam (3rd generation)	Cho et al. (1986) [62]
	Cefepime (4th generation)	Khasani et al. (2015) [63]; Sonck et al. (2008) [64]; Zimmermann et al. (2021) [65]
Carbapenems	Imipenem	Lau et al. (2004) [66]; Rivera et al. (1999) [67]; Frucht et al. (1997) [68]1/15/2025 4:38:00 PM
	Meropenem	Baraboutis et al. (2008) [69]; Spina Silva et al. (2014) [70]
	Ertapenem	Apodaca et al. (2015) [71]
Fluoroquinolones	Ciprofloxacin	Jayathissa et al. (2010) [72]; Post et al. (2004) [73]; Javed et al. (2023) [74]
	Moxifloxacin	Kayipmaz et al. (2017) [75]
	Levofloxacin	Reddy et al. (2020) [76]
	Gatifloxacin	Marinella et al. (2001) [77]
	Ofloxacin	Bagon et al. (1999) [78]
Macrolides	Erythromycin	Michtell et al. (1971) [79]
	Azithromycin	Vadalá et al. (2013) [80]
Others	Aminoglycosides (gentamicin)	Sarva et al. (2012) [81]
	Cotrimoxazole (sulfamethoxazole/ trimethoprim)	Dib et al. (2004) [82]
	Lipopeptides (daptomycin)	Scolari et al. (2021) [83]
	Glycopeptides (vancomycin)	Patel et al. (2018) [84]
	Tetracyclines (doxycycline)	Jacob et al. (2020) [85]
	Oxazolidinones (linezolid)	Ferreira et al. (2024) [86]

Table S4. Antidepressant-Induced Myoclonus.

Subclass	Drug	Reference
Selective serotonin reuptake inhibitors	Citalopram	Forsberg-Gillving et al. (2015) [112]
	Escitalopram	Sato et al. (2015) [113]
	Fluoxetine	Arora et al. (2010) [114]
	Fluvoxamine	Takahashi et al. (2013) [115]
	Paroxetine	Correia et al. (2018) [116]
	Sertraline	Bušková et al. (2012) [117]
Serotonin-norepinephrine reuptake inhibitors	Duloxetine	Trigo López et al. (2021) [118]
	Milnacipran	Koshiishi et al. (2016) [119]
	Venlafaxine	Necpál et al. (2017) [120]
Norepinephrine reuptake inhibitors	Atomoxetine	Sato et al. (2022) [121]
Norepinephrine-dopamine reuptake inhibitors	Bupropion	Riaz et al. (2023) [122]
Noradrenergic and specific serotonergic antidepressant	Mianserin	Majewska et al. (2011) [123]
	Mirtazapine	Hernández et al. (2002) [124]
Serotonin antagonists and reuptake inhibitors	Nefazodone	Mason et al. (2000) [125]
	Trazodone	Patel et al. (1988) [126]
Serotonin modulators and stimulators	Vortioxetine	Thumtecho et al. (2021) [127]
Tricyclic antidepressant	Amitriptyline	Rissardo et al. (2020) [111]
	Clomipramine	Bloem et al. (1999) [128]
	Desipramine	Masand et al. (1992) [129]
	Imipramine	Black et al. (1994) [130]
	Nortriptyline	Mason et al. (2000) [125]
Tetracyclic antidepressant	Maprotiline	Kettl et al. (1983) [131]
Monoamine oxidase inhibitors	Iproniazid	de Larquier et al. (1999) [132]
	Moclobemide	Gillman et al. (1995) [133]
	Phenelzine	White et al. (1987) [134]
	Tranylcypromine	Mason et al. (2000) [125]
Adjunctive therapy	Buspirone	Rissardo et al. (2020) [135]

Table S5. Antipsychotic-Induced Myoclonus.

Subclass	Drug	Reference
Typical	Haloperidol	Hayase et al. (2023) [191]
Atypical	Amisulpride	Altıparmak et al. (2022) [192]
	Clozapine	Berman et al. (1992) [193]
	Chlorpromazine	Leroy et al. (1956) [194]
	Olanzapine	Tikka et al. (2014) [195]
	Perospirone	Ishida et al. (2020) [196]
	Quetiapine	Aggarwal et al. (2008) [197]
	Risperidone	Zand et al. (2010) [198]
	Sulpiride	Asahi et al. (2002) [199]
	Sultopride	Montaz et al. (1992) [200]

Table S6. Antiseizure Medication-Induced Myoclonus.

Drug	Note	Reference
Brivaracetam	No case was found in the literature.	Sáenz-Farret et al. (2022) [217]
Carbamazepine	28 cases in the literature. The mean age was 35.2 years, and the mean dose of carbamazepine was 812.5 mg/day. All the individuals fully recovered after management.	Rissardo et al. (2020) [218]
Cenobamate	No case was found in the literature.	Rissardo et al. (2023) [219]
Clonazepam	No case was found in the literature. There is an improvement in myoclonus with the use of benzodiazepines.	Sáenz-Farret et al. (2022) [217]
Eslicarbazepine	No case was found in the literature.	Rissardo et al. (2020) [218]
Ethosuximide	No case was found in the literature. There is an improvement in myoclonus with the use of ethosuximide.	Wallace et al. (1998) [220]
Felbamate	No case was found in the literature.	Sáenz-Farret et al. (2022) [217]
Gabapentin	135 cases in the literature. The mean age was 53.9 years, and the mean dose of gabapentin was 1277.7 mg/day. 79.2% of the individuals fully recovered after the management.	Rissardo et al. (2023) [221]
Lacosamide	Multifocal electroencephalogram-negative myoclonus has been reported with lacosamide.	Birnbaum et al. (2016) [222]
Lamotrigine	Myoclonus was usually multifocal, and a cortical source was identified. Also, most individuals had been using lamotrigine for months before the occurrence of myoclonus.	Rissardo et al. (2021) [223]
Levetiracetam	No case was found in the literature.	Sáenz-Farret et al. (2022) [217]
Oxcarbazepine	5 cases in the literature. The mean age was 6.2 years, and the mean dose of oxcarbazepine was 900 mg/day. All the individuals fully recovered after management.	Rissardo et al. (2020) [218]
Perampanel	No case was found in the literature. Myoclonus has improved with the use of perampanel.	Thomas et al. (2024) [224]
Phenobarbital	No case was found in the literature with only phenobarbital, but there are some cases of mixtures of medications with phenobarbital causing myoclonus. The use of phenobarbital has improved myoclonus.	Rosen et al. (1969) [225]1/15/2025 4:38:00 PM
Phenytoin	49 cases reported in the literature. The mean age was 70.7 years, and the mean dose of phenytoin was 356.2 mg/day. 90% of the individuals fully recovered after management.	Rissardo et al. (2022) [226]
Pregabalin	Myoclonus was the most commonly reported movement disorder associated with pregabalin. It was multifocal in 48.71% of the patients with bilateral upper limb involvement.	Rissardo et al. (2020) [227]
Primidone	No case was found in the literature. Myoclonus has improved with the use of primidone.	Obeso et al. (1989) [228]
Rufinamide	No case was found in the literature.	Sáenz-Farret et al. (2022) [217]
Stiripentol	No case was found in the literature.	Sáenz-Farret et al. (2022) [217]
Tiagabine	No case was found in the literature. Myoclonus has improved with the use of tiagabine.	Wallace et al. (2001) [229]
Topiramate	Myoclonus associated with topiramate was associated with older individuals and with the male sex.	Rissardo et al. (2020) [230]
Valproate	54 cases reported in the literature. The mean age was 40.2 years, and the mean dose of valproate was 1223 mg/day. 95.2% of the individuals fully recovered after management.	Rissardo et al. (2021) [231]
Vigabatrin	Cortical myoclonus with multifocal distribution has been reported.	Neufeld et al. (1995) [232]
Zonisamide	No case was found in the literature. Myoclonus has improved with the use of zonisamide.	Sáenz-Farret et al. (2022) [217]

Table S7. Opioid-Induced Myoclonus.

Subclass	Drug	Reference
Pure agonists	Dextropropoxyphene	Brefel-Courbon et al. (2006) [9]
	Fentanyl	López Pardo et al. (2016) [262]
	Hydrocodone	Lauterbach et al. (1999) [261]
	Methadone	Sarhill et al. (2001) [263]
	Morphine	Jacobsen et al. (1995) [264]
	Norpethidine	Reutens et al. (1989) [265]
	Oxycodone	Lau et al. (2017) [266]
	Pethidine (meperidine)	Hochman et al. (1983) [267]
	Remifentanil	Delvaux et al. (2005) [268]
	Sulfentanil	Bowdle et al. (1994) [269]
	Tramadol	Bae et al. (2020) [270]
Partial agonists	Buprenorphine	Biedlingmaier et al. (2023) [271]
Pure antagonists	Naloxone	Smolen et al. (1986), in mice models [272]
	Naltrexone	Behnoush et al. (2013) [273]

Table S8. Drug-Induced Asterixis.

Class	Drugs and references
Antibiotics	Amphotericin B [309], cefuroxime [58], ceftazidime [310], cefepime [311], chloramphenicol [312], tretinoin [313], trimethoprim-sulfamethoxazole [314]
Anticholinergic	Oxybutynin chloride [309]
Antiemetics	Metoclopramide [142], ondansetron [147], palonosetron [148], promethazine [145]
Antiparkinsonian	Amantadine [173], levodopa [315]
Antipsychotics	Clozapine [316], olanzapine [309], risperidone [309]
Antiseizure medications	Bromide [309], carbamazepine [317], gabapentin [318], lamotrigine [319], phenytoin [320], pregabalin [321], primidone [322], valproate [323]
Benzodiazepines	Clonazepam [309], lorazepam [309], metrizamide [324]
Histamine receptor modulators	Famotidine [309]
Mood stabilizers	Lithium [325]
Opioids	Hydromorphone [309], meperidine [326]
Others	Ammonium chloride [327], ifosfamide [328], iopamidol [329], salicylates [330]
